# α-Arylsulfonyloxyacrylates: attractive *O*-centered electrophiles for synthesis of α-substituted acrylates *via* Pd-catalysed Suzuki reactions†‡

**DOI:** 10.1039/d3ra00401e

**Published:** 2023-03-20

**Authors:** Zhongya Zhang, Li Zhang, Linge Huai, Zhentao Wang, Yewen Fang

**Affiliations:** a Key Laboratory of Degraded and Unused Land Consolidation Engineering, The Ministry of Land and Resources of China, College of Environmental Science and Engineering, Chang'an University No. 126 Yanta Road Xi'an 710054 China; b School of Materials and Chemical Engineering, Ningbo University of Technology No. 201 Fenghua Road Ningbo 315211 China fang@nbut.edu.cn; c Zhejiang Institute of Tianjin University No. 201 Fenghua Road Ningbo 315211 China; d School of Fundamental Science, Zhejiang Pharmaceutical University No. 666 Siming Road Ningbo 315500 China zhangl@zjpu.edu.cn; e College of Chemistry and Material Science, Shandong Agricultural University No. 61 Daizong Road Tai'an 271018 China wzht423@mail.ustc.edu.cn

## Abstract

We herein report α-arylsulfonyloxyacrylates as a kind of useful and attractive *O*-centered electrophiles for Suzuki cross-coupling reactions. A range of α-(hetero)aryl substituted acrylates has been prepared *via* the palladium-catalysed C–C cross-coupling reactions between potassium (hetero)aryltrifluoroborates and α-arylsulfonyloxyacrylates. Moreover, α-arylsulfonyloxyacrylate could also react with B-alkyl-9-BBN to produce α-alkyl substituted acrylates. The synthetic application of this new method was demonstrated by the preparation of the intermediate for synthesis of retinoid X receptors-selective retinoids. These Suzuki reaction-based protocols feature broad substrate scope, generality, and mild reaction conditions.

## Abstract 2

With α-arylsulfonyloxyacrylates as the new *O*-centered electrophiles, α-substituted acrylates could be efficiently accessed *via* Pd-catalysed Suzuki (hetero)arylation and alkylation reactions.

## Graphical abstract

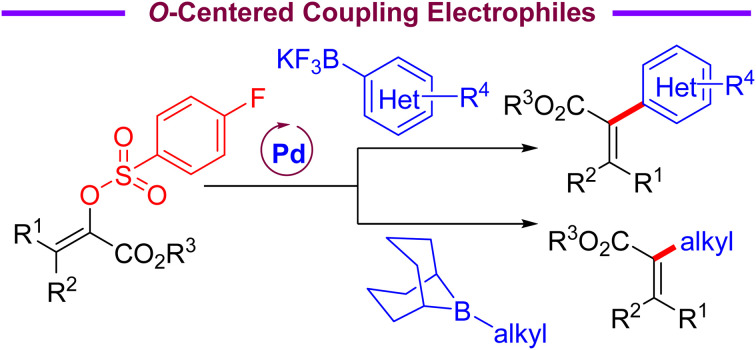

## Introduction

**Fig. 1:**
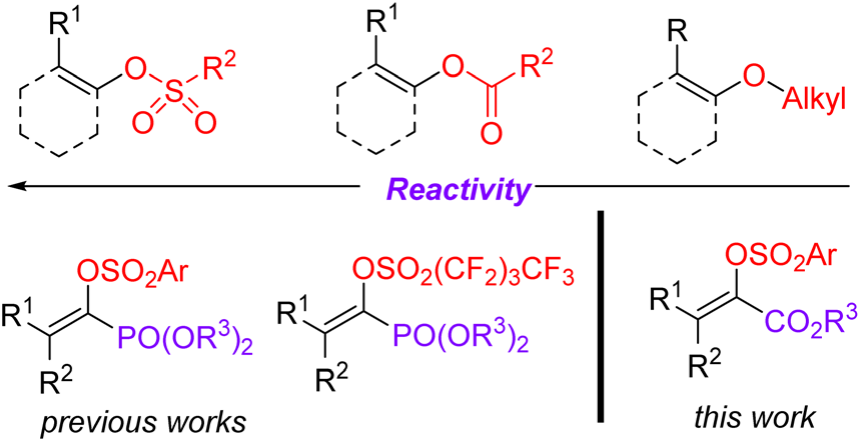
*O*-Centered coupling electrophiles.

The Suzuki cross-coupling reaction is considered to be one of the most robust methods in modern organic synthesis, providing a rapid and straightforward strategy for constructing C–C bond formation.^1^ Taking advantage of the easy availability of coupling partners as well as their stabilities towards air and moisture, the synthetic community would like to choose the Suzuki reaction as their choice for C–C bond formation.^2^ Due to the marvelous progress on the supporting ligands^3^ and the preparation of organoboron derivatives,^4^ protocols based on Suzuki reactions become more reliable and practical. Moreover, the applications of non-noble metal catalysts^5^ and the use of continuous-flow reactors for Suzuki reactions meet the requirement of sustainable development of chemistry.^6^ Of note, the development of reliable electrophiles has also been the subject of Suzuki cross-coupling reactions.^7^ As for the available electrophilic Suzuki coupling partners, the utilization of C–O electrophiles as surrogates for organic halides is especially attractive due to their flexibility and generality as well as practicality. Compared to the many well-documented methods for synthesis of aromatic halides, the site-specific preparation of alkenyl halides with expected configuration is still a big challenge. Consequently, there has been increasing interest on the Suzuki cross-coupling reactions using enol-based compounds as the coupling electrophiles. Among the many enol-derived electrophiles, alkenyl sulfonates are especially attractive due to their good stability and high reactivity (Fig. 1).^8^

**Scheme 1:**
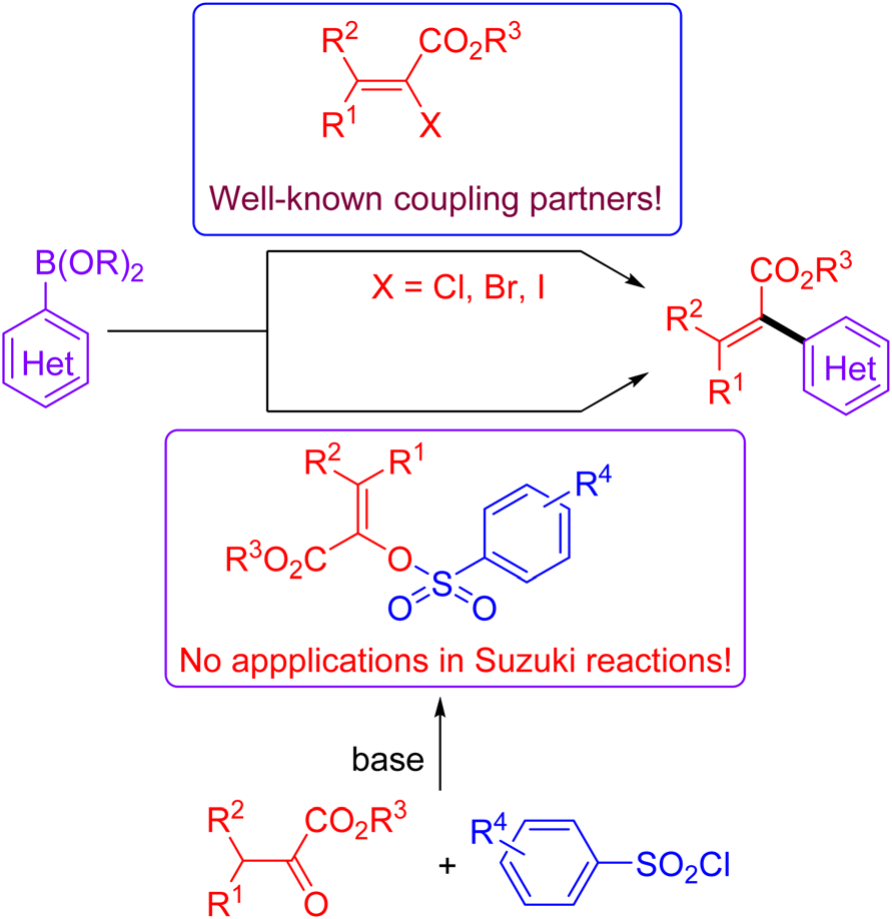
Suzuki reactions for synthesis of α-(hetero)aryl substituted acrylates.

Acrylates and their derivatives are a kind of fundamental monomers and structural motifs. In addition to the wide applications in polymer chemistry, α-substituted acrylates are useful acceptors for both nucleophiles and nucleophilic radicals.^9^ As a result, many efforts have been devoted to their preparation and the further transformations. Among the available strategies, transition-metal-catalysed cross-coupling reactions are undoubtedly indispensable tools. Interestingly, an access to α,β-unsaturated esters has been realized *via* palladium-catalysed reactions using diazo compounds as the coupling partners.^10^ Not surprisingly, the Suzuki reaction-based protocols for the preparation of α-substituted acrylates have been extensively investigated.^11^ In contrast to the applications of α-halo acrylates in Suzuki reactions, there is still no available reports dealing with the preparation of α-substituted acrylates using *O*-centered coupling electrophiles (Scheme 1). Inspired by our recent works on the Suzuki cross-coupling reactions using α-phosphonovinyl arylsulfonates as the electrophilic coupling partners (Fig. 1),^12^ we wonder that α-arylsulfonyloxyacrylates could serve as the *O*-centered electrophile candidate. According to the available reports, α-arylsulfonyloxyacrylates could be easily prepared from inexpensive aryl sulfonyl chloride with pyruvate derivatives in presence of base. Moreover, in addition to environmentally benign character, the α-arylsulfonyloxyacrylates would be more stable than α-haloacrylates coupling partners. We herein report a new protocol for synthesis of α-(hetero)aryl acrylates *via* the Suzuki reactions between arylsulfonyloxyacrylates and potassium (hetero)aryltrifluoroborates enabled by palladium catalysis.^13^ Moreover, α-alkyl acrylates were also prepared *via* the palladium catalysed C–C cross-coupling reactions of α-arylsulfonyloxyacrylates with B-alkyl-9-BBN.^14^

## Results and discussion

We began our studies by investigating the Suzuki coupling reaction of potassium phenyltrifluoroborate 2a with 1a derived from ethyl pyruvate and 4-fluorobenzenesulfonyl chloride. As outlined in Table 1, a simple survey of experimental parameters led us to identify the optimal reaction conditions (5 mol% Pd(OAc)_2_, 10 mol% SPhos, 1.5 equiv. of K_3_PO_4_, 3 : 1 mixed CH_3_Ph/H_2_O, 60 °C, 24 h) (entry 1).^15^ Other Buchwald-type ligands were screened and no better result was observed (entries 2–4). The combination of Pd(OAc)_2_ and *rac*-BINAP did not lead to the expected product formation (entry 5). With tricyclohexylphosphine as the supporting ligand, a mixture of 3a and 3aa was observed (entry 6). Possibly, the side product 3aa would be formed when the β-hydride elimination pathway took place ahead of transmetallation process.^16^ Again, we found that a mixture of 3a and 3aa was achieved using Pd(OAc)_2_, *t*-Bu_3_PH·BF_4_, and K_3_PO_4_ (entry 7). Other inorganic bases such as Cs_2_CO_3_, K_2_CO_3_, and *t*-BuOK gave inferior results (entries 8–10). Moreover, no better results were observed using other palladium catalysts (entries 11 and 12). As for the electrophiles, α-tosyloxyacrylate 1b could serve as the alternative, albeit in low yield (entry 13). However, no desired product could be observed using α-ethylsulfonyloxyacrylate 1c as the coupling partner (entry 14). With phenylboronic acid instead of 2a as the nucleophilic coupling partner, the desired product 3a was contaminated by the formation of 3aa (entry 15). Similar result was achieved using phenylboronic acid pinacol ester as the coupling partner (entry 16). In the absence of base, treatment of 1a with 2a under the palladium catalysed reaction conditions gave 3a in 49% yield (entry 17). Control experiments confirmed that both ligand and catalyst were required for this transformation (entries 18 and 19).

**Table 1:** Reaction optimization^a^^,^^b^

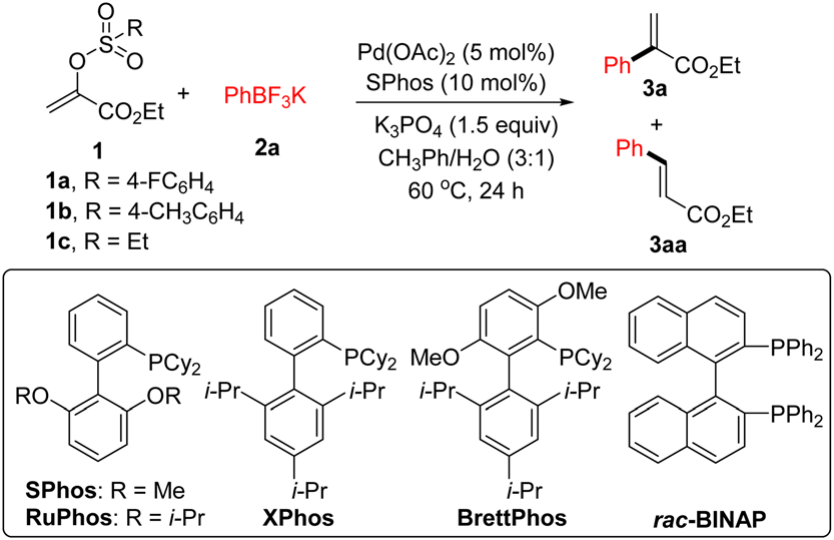		
Entry	Deviation from standard conditions	Yield of 3a (%)
1	None	73
2	RuPhos instead of SPhos	49
3	XPhos instead of SPhos	67
4	BrettPhos instead of SPhos	27
5^c^	*rac*-BINAP instead of SPhos	0
6^c^^,^^d^	Cy_3_PH·BF_4_ instead of SPhos	36 (12)
7^c^^,^^d^	*t*-Bu_3_PH·BF_4_ instead of SPhos	37 (28)
8^c^^,^^d^	Cs_2_CO_3_ instead of K_3_PO_4_	52 (7)
9^c^^,^^d^	K_2_CO_3_ instead of K_3_PO_4_	52 (9)
10	*t*-BuOK instead of K_3_PO_4_	52
11	PdCl_2_ instead of Pd(OAc)_2_	62
12	Pd_2_(dba)_3_ instead of Pd(OAc)_2_	45
13	1b instead of 1a	37
14^c^	1c instead of 1a	0
15^c^^,^^d^	PhB(OH)_2_ instead of 2a	40 (20)
16^c^^,^^d^	PhBpin instead of 2a	31(16)
17	Without base	49
18^c^	Without ligand	0
19^c^	Without catalyst	0

a Standard reaction conditions: a reaction mixture of 1a (0.2 mmol), 2a (0.26 mmol), Pd(OAc)_2_ (5 mol%), SPhos (10 mol%), K_3_PO_4_ (0.3 mmol), and CH_3_Ph/H_2_O (3.0 mL/1.0 mL) was stirred at 60 °C for 24 h.

b Yield of the isolated product 3a.

c NMR yield (500 MHz) was reported by use of *p*-nitroacetophenone as an internal standard.

d NMR yields of the 3aa were reported in the brackets.

After the optimal reaction conditions were established for this Pd-catalysed Suzuki reactions, the scope of aryl trifluoroborates and α-arylsulfonyloxyacrylates was initially tested (Table 2). A range of potassium aryltrifluoroborate was firstly investigated. Generally, both electron-rich and electron-deficient aryl trifluoroborates proved viable coupling partners, giving 3b–3o in 31–83% yields. Notably, steric variance on the phenyl ring did not lead to an obvious influence on the reaction efficiency. The aryl trifluoroborates having *para*- (3b), *meta*- (3c, 3e), or *ortho*- (3d) substituents were all eligible to forge the desired ethyl α-aryl acrylates. Pleasingly, the cross-coupling reactions of substituted aryl trifluoroborates (-ethyl, -*tert*-butyl, and -methoxyl) worked well to give the desired products 3f–3j in 58–83% yields. Noticeably, chloro substituent is well tolerated in this reaction conditions (3k and 3l), which is advantageous for the further decoration. Likewise, potassium 4-fluorophenyltrifluoroborate could be coupled with 1a using this new protocol, obtaining 3m in moderate yield. Moreover, aryl trifluoroborates bearing a naphthalene ring can be transformed to the corresponding products 3n and 3o in 52% and 61% yields, respectively. After the evaluation of scope of aryl trifluoroborates, various alkyl α-phenylacrylates were prepared under the standard cross-coupling reaction conditions. In addition to efficient access to methyl and iso-propyl α-phenylacrylates (3p, 3q), cyclohexyl α-phenylacrylate (3r) could be nicely prepared in 66% yield. Additionally, benzyl α-phenylacrylates (3s, 3t) were also successfully prepared in efficient manner.

**Table 2:** Arylations of α-arylsulfonyloxyacrylates 1^a^

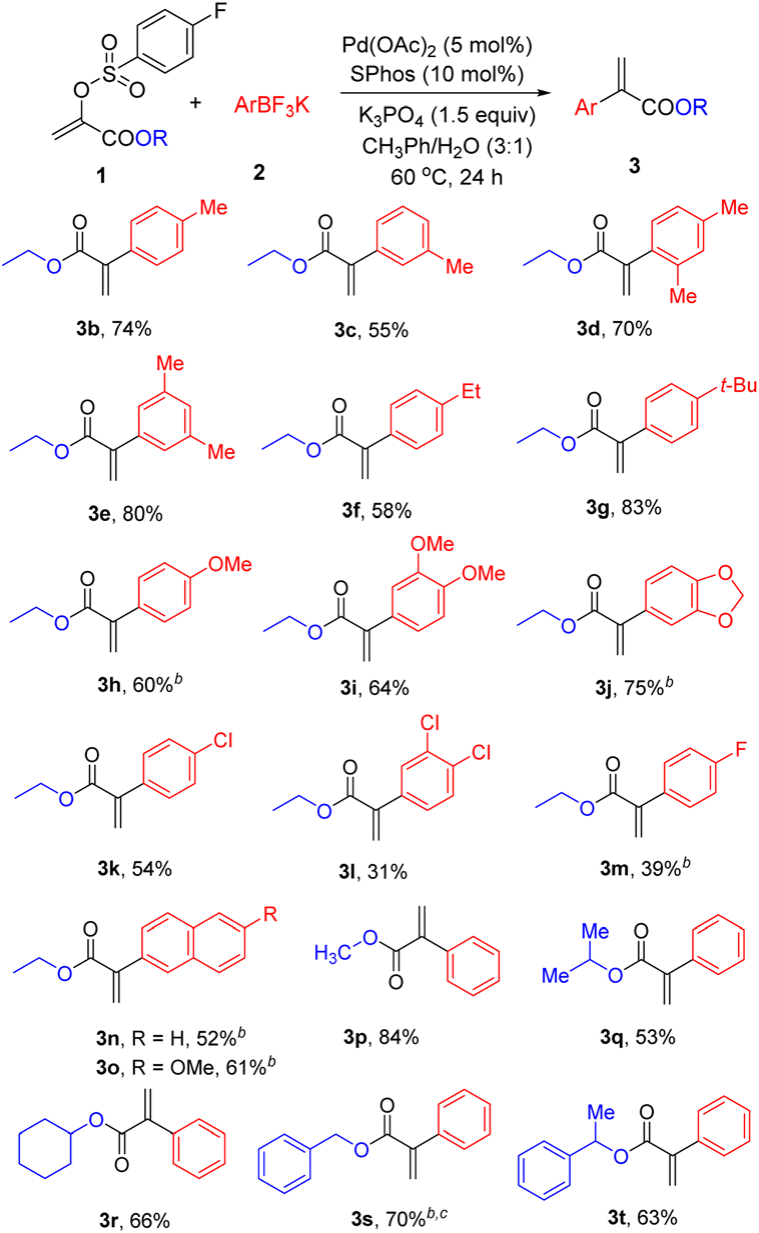

a Reaction conditions: see entry 1, Table 1. Isolated yields.

b A mixture of 1,4-dioxane/H_2_O (3.0 mL/1.0 mL) was used.

c The reaction temperature was 80 °C.

After realizing the synthesis of α-aryl acrylates, we set out to prepare tetrasubstituted α,β-unsaturated esters under the above optimized catalyst system. As shown in Table 3, a range of β,β-disubstituted-α-aryl acrylates could be synthesized *via* the reactions between β,β-disubstituted-α-arylsulfonyloxyacrylates and aryl trifluoroborates enabled by palladium catalysis. Due to the possible steric effect, increasing the reaction temperature to 80 °C was required for preparation of tetrasubstituted olefins using mixed 1,4-dioxane/H_2_O as the solvent. Notably, aryl trifluoroborates bearing a methoxyl group at *ortho*-, *meta*-, or *para*-positions on the phenyl ring, were well-tolerated, giving the corresponding products 5c–5e in 59–65% yields. In addition to aryl trifluoroborates bearing electron-neutral and electron-donating substituents, fluoro and chloro substituents were well both accommodated, furnishing the expected products 5g and 5h in 61% and 51% yields, respectively. In addition to the smooth reaction of potassium (2-naphthalene)trifluoroborate with 4a, a sterically demanding potassium (1-naphthalene)trifluoroborate was compatible in this reaction, furnishing 5j in 61% yield. Gratifyingly, potassium 2-thienyltrifluoroborate could undergo coupling reaction with 4a to afford 5k in 46% yield. After the successful synthesis of various ethyl 3-methyl-2-aryl-2-butenoates 5a–5k, β,β-diethyl substituted α-phenyl acrylate 5l could be expectedly prepared in 70% yield. To our delight, a range of exocyclic olefins 5m–5o could be obtained in 50–55% yields.

**Table 3:** Synthesis of tetrasubstituted olefins 5^a^

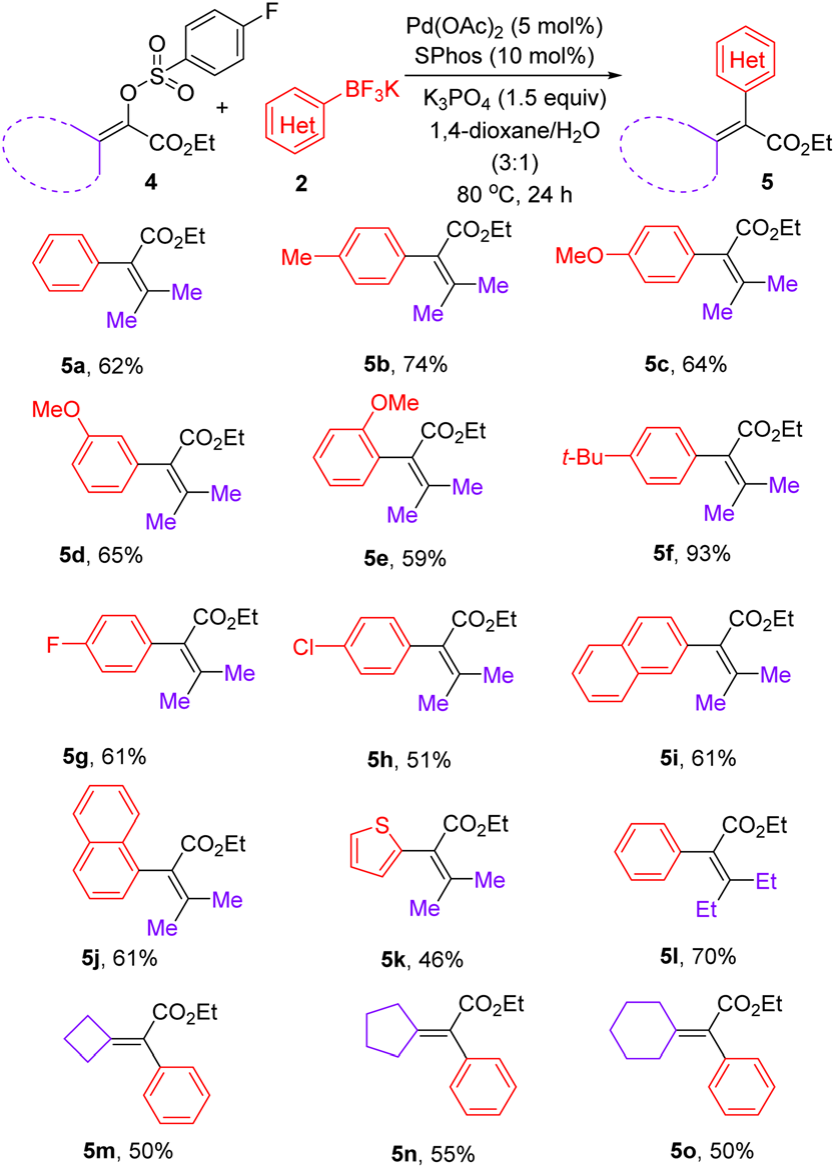

a Reaction conditions: a reaction mixture of 4 (0.2 mmol), 2 (0.3 mmol), Pd(OAc)_2_ (5 mol%), SPhos (10 mol%), K_3_PO_4_ (0.3 mmol), and 1,4-dioxane/H_2_O (3.0 mL/1.0 mL) was stirred at 80 °C for 24 h.

**Scheme 2:**
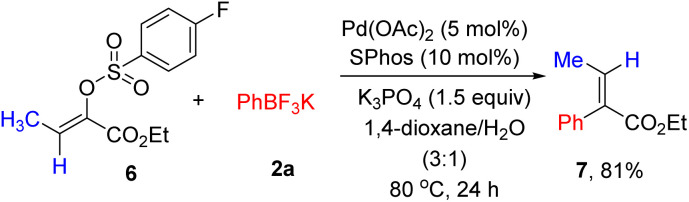
Stereoselective cross-coupling reaction.

Next, the stereoselective cross-coupling of α-arylsulfonyloxyacrylate 6 with potassium phenyltrifluoroborate 2a was briefly investigated. As shown in Scheme 2, full conversion could be achieved and no trace of the alkene geometry erosion was observed, producing (*E*)-ethyl 2-phenyl-2-butenoate 7 in 81% yield. The results listed in Tables 2, 3 and Scheme 2 nicely demonstrated the generality of (hetero)arylation of arylsulfonyloxyacrylates.

**Scheme 3:**
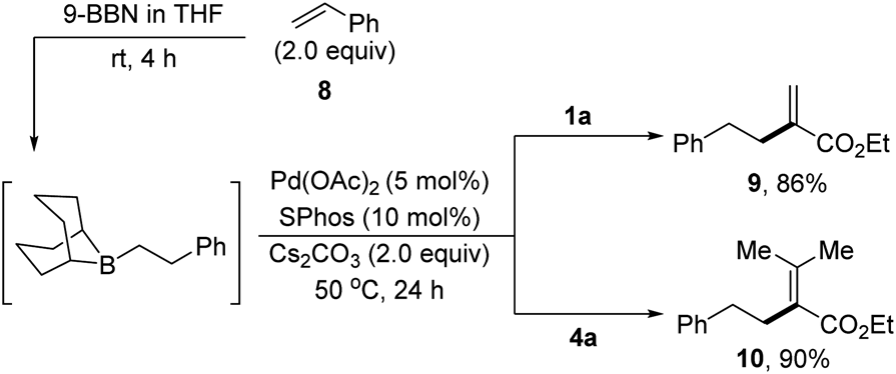
B-alkyl Suzuki cross-coupling reactions.

As an extension, the B-alkyl Suzuki cross coupling reactions have been demonstrated with α-arylsulfonyloxyacrylates as the electrophilic coupling partners. As shown in Scheme 3,^15*e*^ the hydroboration of styrene 8 with 9-BBN (THF, rt) afforded the corresponding B-phenylethyl-9-BBN, which was *in situ* treated with Cs_2_CO_3_ and 1a in the presence of Pd(OAc)_2_ and SPhos in THF at 50 °C for 24 hours, producing the desired cross-coupled product 9 in 86% yield. Likewise, tetrasubstituted alkene 10 could be prepared in 90% yield *via* the reactions of B-phenylethyl-9-BBN with 4a.

**Scheme 4:**
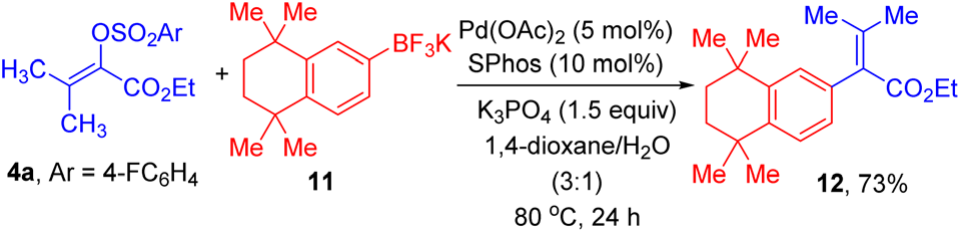
Synthetic demonstration.

Lastly, a demonstration of the synthetic value of this method is given by the preparation of 12, which is an important intermediate for synthesis of retinoid X receptors-selective retinoids.^17^ As displayed in Scheme 4, efficient C–C cross-coupling reaction for formation of 12 could take place between 4a and aryl trifluoroborate 11 under the standard reaction conditions.

**Scheme 5:**
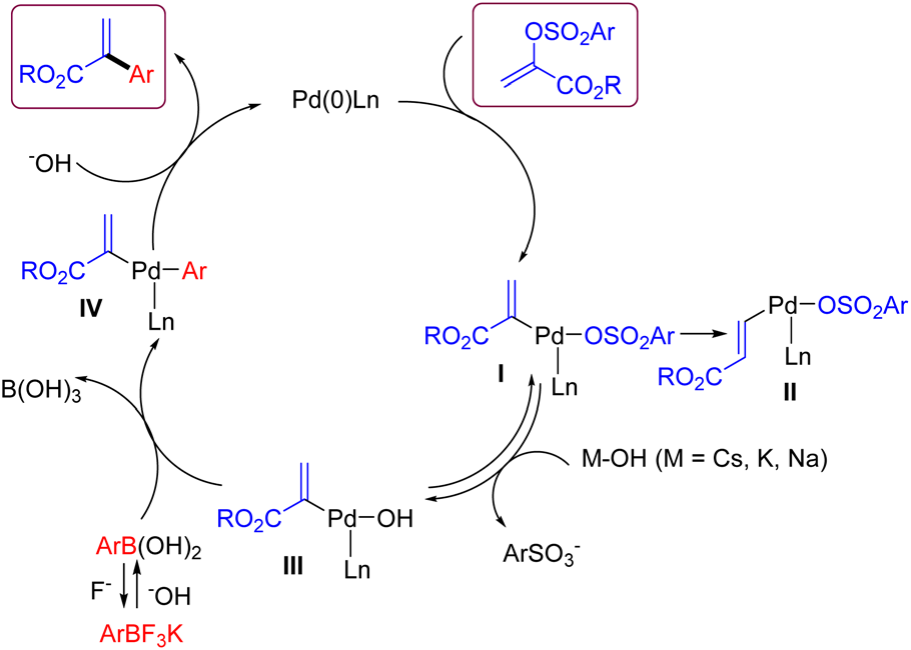
Proposed mechanistic pathway.

According to the literature reports and our results,^18^ a plausible reaction mechanism for this Suzuki cross-coupling reaction between α-arylsulfonyloxyacrylate and aryl trifluoroborate is depicted in Scheme 5. The oxidative addition of α-arylsulfonyloxyacrylate onto a ligated Pd(0) species generates arylpalladium(ii) complex I. At this stage, a side intermediate II for the formation of alkyl cinnamate would be formed *via* a competitive pathway involving β–H elimination of I followed by reinsertion of Pd–H species.^16^ As for the expected pathway, the more reactive organopalladium hydroxide III is formed from the I*via* the displacement of arylsulfonate ion with HO^−^ anion. Then transmetallation with arylboronic acid takes place to give intermediate IV. Finally, reductive elimination of IV affords the coupling product and regenerates the Pd(0) species with the aid of base.

## Conclusions

In summary, we have reported α-arylsulfonyloxyacrylates as the useful *O*-centered electrophiles for the Pd-catalysed Suzuki cross-coupling reactions. Using functionalized potassium (hetero)aryltrifluoroborates as the nucleophilic coupling partners, a list of α-(hetero)aryl substituted acrylates could be modularly prepared *via* the C–C cross-coupling reactions. Moreover, α-alkyl substituted acrylates could be also efficiently accessed *via* palladium-catalyzed reactions of α-arylsulfonyloxyacrylate with B-alkyl-9-BBN. We anticipate that these new developed α-arylsulfonyloxyacrylates could find more application for the synthesis of functionalized α,β-unsaturated esters *via* various C–C cross-coupling reactions enabled by transition-metal catalysis and beyond.

## Author contributions

YF, LZ, and ZW conceived the idea and designed the research. ZZ and LH performed the research. ZZ, LH, and YF analyzed the data. YF and ZZ wrote the original manuscript. YF and LZ reviewed the manuscript and suggested improvements.

## Conflicts of interest

There are no conflicts to declare.

## Supplementary Material

RA-013-D3RA00401E-s001
