# Supplementary material for: α-Arylsulfonyloxyacrylates: attractive O-centered electrophiles for synthesis of α-substituted acrylates via Pd-catalysed Suzuki reactions
Source: RSC Adv. 2023 Mar 20;13(14):9180–5. doi: 10.1039/d3ra00401e (PMC10026373; doi:10.1039/d3ra00401e)
Supplement: RA-013-D3RA00401E-s001 [file RA-013-D3RA00401E-s001.pdf]

Electronic Supplementary Material (ESI) for RSC Advances.
This journal is © The Royal Society of Chemistry 2023

Supporting Information for

α-Arylsulfonyloxyacrylates: attractive *O*-centered electrophiles for synthesis of α-substituted acrylates via Pd-catalysed Suzuki reactions

Zhongya Zhang, Li Zhang, Linge Huai, Zhentao Wang and Yewen Fang

Table of Contents

1 General Information S2

1.1 Solvents, Reagents, and Starting Materials S2

1.2 Instruments S2

2 General Procedure of Synthesis of α-Arylsulfonyloxyacrylates S3

3 General Procedure of Suzuki Reaction S7

3.1 General Procedure of the (Hetero)arylation Reactions of α-Arylsulfonyloxyacrylates .S7

3.2 General Procedure of the Alkylation Reactions of α-Arylsulfonyloxyacrylates..........S17

4 References S19

5 NMR Spectra of New Compounds S20

1 General Information

1.1 Solvents, Reagents, and Starting Materials

All reactions were carried out under an atmosphere of nitrogen in oven-dried glassware. The α-ketoesters are commercially available or prepared following literature known methods.[1] The potassium (hetero)aryltrifluoroborates were reported by our previous work or prepared according to the literature procedures.[2] The employed phosphorus ligands are commercially available. The dried solvents were obtained from commercial sources and used without further purification unless otherwise noted.

1.2 Instruments

NMR spectra were recorded on a BrukerAvance 500 spectrometer (500 MHz) (500 MHz for ^{1}H NMR, 126 MHz for ^{13}C NMR, and 471 MHz for ^{19}F NMR). Chemical shifts were reported in ppm downfield from tetramethylsilane and calibrated using residue undeuterated solvent (Chloroform-*d* at 7.26 ppm ^{1}H NMR; 77.0 ppm ^{13}C NMR). Spectra were reported as follows: chemical shift (δ ppm), multiplicity (s = singlet, d = doublet, t = triplet, q = quartet, m = multiplet, br = broad). Coupling constants are reported in Hertz where available. High resolution mass spectra (HRMS) were recorded on Waters Premier GC-TOF MS, Waters G2-Xs QTOF MS, and JEOL-AccuTOF-GCv4G-GCT MS. Analytical thin layer chromatography was performed on Polygram SIL G/UV254 plates. Visualization was accomplished with short wave UV light, or $KMnO_4$ staining solutions. Flash column chromatography was performed using silica gel (300-400 mesh) with solvents to use.

2 General Procedure of Synthesis of α-Arylsulfonyloxyacrylates

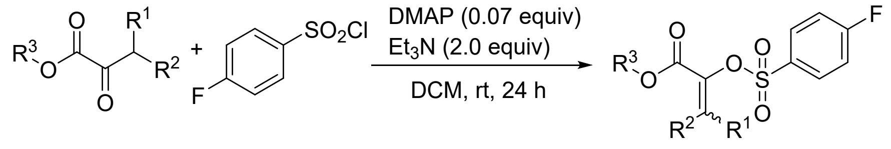

A mixture of α-ketoester (15 mmol, 1.0 equiv), *p*-fluorosulfonyl chloride (3.5 g, 18 mmol), DMAP (134.5 mg, 1.1 mmol) was dissolved in dichloromethane (30 mL). Triethylamine (4.1 mL, 29.5 mmol) was added dropwise and reacted for 24 h. At the end of the reaction, the mixture was then quenched with H_2O (20 mL). The organic phase was separated and the aqueous phase extracted with ethyl acetate (3 x 15 mL). The combined organic phase was dried by $MgSO_4$ and the solvent removed *in vacuo*. Crude products were purified by silica gel column chromatography.

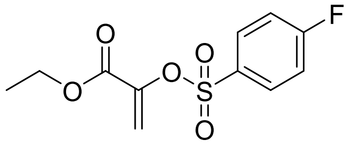

1a

Ethyl 2-(((4-fluorophenyl)sulfonyl)oxy)acrylate (1a). Flash column chromatography to afford product **1a** as a yellow liquid. ^{1}H NMR (500 MHz, $CDCl_3$) δ 7.93 – 7.86 (m, 2H), 7.18 (t, *J* = 8.6 Hz, 2H), 6.08 (d, *J* = 2.5 Hz, 1H), 5.57 (d, *J* = 2.5 Hz, 1H), 4.06 (q, *J* = 7.2 Hz, 2H), 1.12 (t, *J* = 7.2 Hz, 3H). ^{13}C NMR (126 MHz, $CDCl_3$) δ 165.7 (d, *J* = 258.3 Hz), 160.3, 142.8, 131.3 (d, *J* = 2.5 Hz), 131.2 (d, *J* = 10.1 Hz), 116.9, 116.2 (d, *J* = 22.7 Hz), 61.7, 13.5. ^{19}F NMR (471 MHz, $CDCl_3$) δ -102.16. HRMS (ESI) $[M+Na]^+$: calculated for$C_{11}H_{11}FO_5SNa$: 297.0209, found 297.0210.

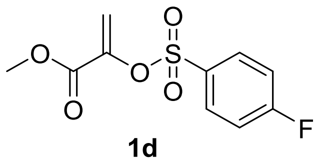

1d

Methyl 2-(((4-fluorophenyl)sulfonyl)oxy)acrylate (1d). Flash column chromatography to afford product **1d** as a yellow liquid. ^{1}H NMR (500 MHz, $CDCl_3$) δ 7.99 – 7.91 (m, 2H), 7.22 (t, *J* = 8.5 Hz, 2H), 6.13 (d, *J* = 2.5 Hz, 1H), 5.64 (d, *J* = 2.6 Hz, 1H), 3.67 (s, 3H). ^{13}C NMR (126 MHz, $CDCl_3$) δ 166.0 (d, *J* = 258.3 Hz), 161.1, 142.7, 131.4 (d, *J* = 2.5 Hz), 131.3 (d, *J* = 10.1 Hz), 117.40, 116.4 (d, *J* = 25.2 Hz), 52.6. ^{19}F NMR (471 MHz, $CDCl_3$) δ -101.82. HRMS (ESI) $[M+Na]^+$: calculated for $C_{10}H_9FO_5SNa$: 283.0052, found 283.0059.

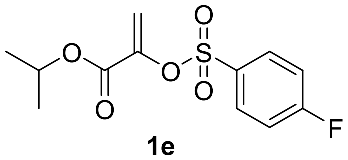

1e

Isopropyl 2-(((4-fluorophenyl)sulfonyl)oxy)acrylate (1e). Flash column chromatography to afford product **1e** as a yellow liquid. ^{1}H NMR (500 MHz, $CDCl_3$) δ 8.06 – 7.85 (m, 2H), 7.38 – 7.09 (m, 2H), 6.15 (s, 1H), 5.68 (d, J = 2.3 Hz, 1H), 4.99 (p, J = 6.3 Hz, 1H), 1.21 (d, J = 6.4 Hz, 6H). ^{13}C NMR (126 MHz, $CDCl_3$) δ 166.1 (d, J = 258.3 Hz), 160.2, 143.4, 131.8 (d, J = 2.5 Hz), 131.5 (d, J = 10.1 Hz), 117.0, 116.5 (d, J = 23.9 Hz), 70.2, 21.5. ^{19}F NMR (471 MHz, $CDCl_3$) δ -102.0. HRMS (ESI) $[M+Na]^+$: calculated for $C_{12}H_{13}FO_5SNa$: 311.0362, found 311.0364.

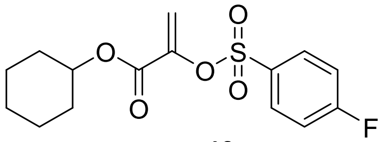

1f

Cyclohexyl2-(((4-fluorophenyl)sulfonyl)oxy)acrylate (1f). Flash column chromatography to afford product **1f** as a yellow liquid. ^{1}H NMR (500 MHz, $CDCl_3$) δ 8.08 – 7.97 (m, 2H), 7.26 – 7.21 (m, 2H), 6.15 (d, J = 2.3 Hz, 1H), 5.66 (d, J = 2.3 Hz, 1H), 4.80 – 4.74 (m, 1H), 1.80 – 1.67 (m, 4H), 1.54 – 1.49 (m, 1H), 1.43 – 1.24 (m, 5H). ^{13}C NMR (126 MHz, $CDCl_3$) δ 166.1 (d, J = 257.0 Hz), 160.1, 143.4, 131.7 (d, J = 2.5 Hz), 131.4 (d, J = 10.1 Hz), 116.9, 116.5 (d, J = 25.2 Hz), 74.8, 31.2, 25.2, 23.4. ^{19}F NMR (471 MHz, $CDCl_3$) δ -102.02. HRMS (ESI) $[M+Na]^+$: calculated for $C_{15}H_{17}FO_5S$ Na: 351.0678, found 351.0685.

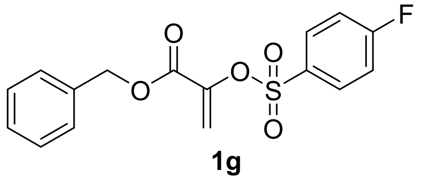

1g

Benzyl 2-(((4-fluorophenyl)sulfonyl)oxy)acrylate (1g). Flash column chromatography to afford product **1g** as a yellow liquid. ^{1}H NMR (500 MHz, $CDCl_3$) δ 7.93 – 7.90 (m, 2H), 7.38 – 7.35 (m, 3H), 7.30 – 7.26 (m, 2H), 7.10 (t, J = 8.5 Hz, 2H), 6.23 (d, J = 2.3 Hz, 1H), 5.76 (d, J = 2.4 Hz, 1H), 5.13 (s, 2H). ^{13}C NMR (126 MHz, $CDCl_3$) δ 165.9 (d, J = 258.3 Hz), 160.6, 142.8, 134.7, 131.4 (d, J = 8.8 Hz), 131.3 (d, J = 3.8 Hz), 128.6, 128.5, 128.4, 117.9, 116.4 (d, J = 25.2 Hz), 67.7. ^{19}F NMR (471 MHz, $CDCl_3$) δ -101.64. HRMS (ESI) $[M+Na]^+$: calculated for $C_{16}H_{13}FO_5S$ Na: 359.0365, found 359.0368.

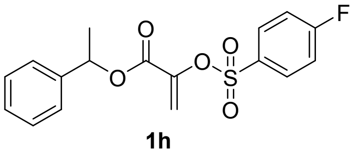

1h

1-Phenylethyl 2-(((4-fluorophenyl)sulfonyl)oxy)acrylate (1h). Flash column chromatography to afford product **1h** as a colorless liquid. ^{1}H NMR (500 MHz, $CDCl_3$) δ 7.94 – 7.85 (m, 2H), 7.41 – 7.26 (m, 5H), 7.19 – 7.06 (m, 2H), 6.22 (d, J = 2.4 Hz, 1H), 5.85 (q, J = 6.6 Hz, 1H), 5.74 (d, J = 2.4 Hz, 1H), 1.53 (d, J = 6.6 Hz, 3H). ^{13}C NMR (126 MHz, $CDCl_3$) δ 165.9 (d, J = 258.3 Hz), 159.9, 143.1, 140.4, 131.4 (d, J = 3.8 Hz), 131.3 (d, J = 10.1 Hz), 128.5, 128.2, 126.1, 117.5, 116.4 (d, J = 22.7 Hz), 74.6, 21.8. ^{19}F NMR (471 MHz, $CDCl_3$) δ -101.73. HRMS (ESI) $[M+Na]^+$: calculated for $C_{17}H_{15}FO_5SNa$: 373.0522, found 373.0526.

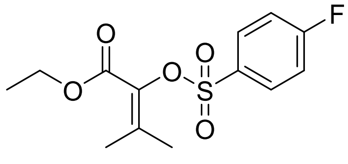

4a

Ethyl 2-(((4-fluorophenyl)sulfonyl)oxy)-3-methylbut-2-enoate (4a). Flash column chromatography to afford product **4a** as a colorless liquid. ^{1}H NMR (500 MHz, $CDCl_3$) δ 8.01 – 7.99 (m, 2H), 7.24 (t, J = 8.5 Hz, 2H), 4.10 (q, J = 7.1 Hz, 2H), 2.18 (s, 3H), 1.82 (s, 3H), 1.20 (t, J = 7.2 Hz, 3H). ^{13}C NMR (126 MHz, $CDCl_3$) δ 165.8 (d, J = 257.0 Hz), 162.0, 144.5, 132.9, 132.8(d, J = 13.8 Hz), 131.1(d, J = 8.8 Hz), 116.3 (d, J = 25.2 Hz), 61.1, 21.3, 20.4, 13.9. ^{19}F NMR (471 MHz, $CDCl_3$) δ -102.73. HRMS (ESI) $[M+Na]^+$: calculated for $C_{13}H_{15}FO_5SNa$: 325.0522, found 325.0524.

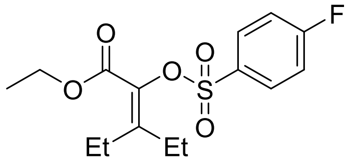

4b

Ethyl 3-ethyl-2-(((4-fluorophenyl)sulfonyl)oxy)pent-2-enoate (4b). Flash column chromatography to afford product **4b** as a colorless liquid. ^{1}H NMR (500 MHz, $CDCl_3$) δ 8.01 – 7.97 (m, 2H), 7.25 – 7.20 (m, 2H), 4.11 (q, J = 7.1 Hz, 2H), 2.50 (q, J = 7.5 Hz, 2H), 2.15 (q, J = 7.6 Hz, 2H), 1.21 (t, J = 7.1 Hz, 3H), 1.09 (t, J = 7.5 Hz, 3H), 0.97 (t, J = 7.6 Hz, 3H). ^{13}C NMR (126 MHz, $CDCl_3$) δ 165.8 (d, J = 258.3 Hz), 162.1, 153.8, 132.7 (d, J = 3.8 Hz), 132.4, 131.1 (d, J = 8.8 Hz), 116.3 (d, J = 25.2 Hz), 61.2, 24.6, 23.9, 13.9, 12.9, 11.6. ^{19}F NMR (471 MHz, $CDCl_3$) δ -102.76. HRMS (ESI) $[M+H]^+$: calculated for $C_{15}H_{19}FO_5S$: 331.1015, found 331.1002.

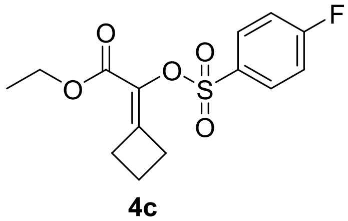

4c

Ethyl 2-cyclobutylidene-2-(((4-fluorophenyl)sulfonyl)oxy)acetate (4c). Flash column chromatography to afford product **4c** as a yellow liquid. ^{1}H NMR (500 MHz, $CDCl_3$) δ 7.96 – 7.93 (m, 2H), 7.26 – 7.14 (m, 2H), 4.01 (q, *J* = 7.1 Hz, 2H), 3.07 – 3.03 (m, 2H), 2.78 – 2.74 (m, 2H), 1.98 (p, *J* = 7.9 Hz, 2H), 1.11 (t, *J* = 7.2 Hz, 3H). ^{13}C NMR (126 MHz, $CDCl_3$) δ 165.6 (d, *J* = 257.0 Hz), 161.2, 156.4, 132.4 (d, *J* = 2.5 Hz), 131.1 (d, *J* = 10.1 Hz), 129.5, 116.0 (d, *J* = 22.7 Hz), 60.8, 31.4, 29.7, 16.8, 13.8. ^{19}F NMR (471 MHz, $CDCl_3$) δ -102.88.HRMS (ESI) $[M+Na]^+$: calculated for $C_{14}H_{15}FO_5SNa$: 337.0522, found 337.0523.

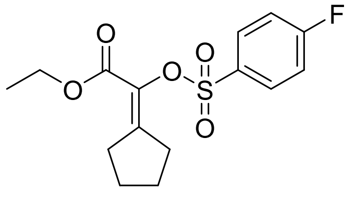

4d

Ethyl 2-cyclopentylidene-2-(((4-fluorophenyl)sulfonyl)oxy)acetate (4d). Flash column chromatography to afford product **4d** as a yellow liquid. ^{1}H NMR (500 MHz, $CDCl_3$) δ 8.01 – 7.98 (m, 2H), 7.23 – 7.18 (m, 2H), 4.05 (t, *J* = 7.1 Hz, 2H), 2.81 – 2.76 (m, 2H), 2.57 – 2.53 (m, 2H), 1.75 (p, *J* = 6.9 Hz, 2H), 1.64 (q, *J* = 7.1 Hz, 2H), 1.15 (t, *J* = 7.1 Hz, 3H). ^{13}C NMR (126 MHz, $CDCl_3$) δ 165.7 (d, *J* = 257.0 Hz), 161.8, 158.2, 133.1 (d, *J* = 3.8 Hz), 133.1 (d, *J* = 10.1 Hz), 130.2, 116.2 (d, *J* = 22.7 Hz), 60.9, 33.4, 32.7, 26.6, 25.4, 13.9. ^{19}F NMR (471 MHz, $CDCl_3$) δ -103.03. HRMS (ESI) $[M+Na]^+$: calculated for $C_{15}H_{17}FO_5SNa$: 351.0678, found 351.0679.

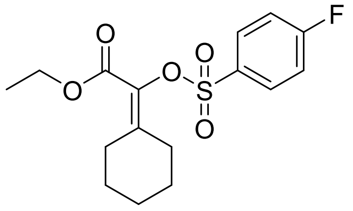

4e

Ethyl 2-cyclohexylidene-2-(((4-fluorophenyl)sulfonyl)oxy)acetate (4e). Flash column chromatography to afford product **4e** as a white solid. ^{1}H NMR (500 MHz, $CDCl_3$) δ 7.99 – 7.92 (m, 2H), 7.24 – 7.18 (m, 2H), 4.07 (q, *J* = 7.1 Hz, 2H), 2.72 – 2.66 (m, 2H), 2.17 (m, *J* = 6.8, 5.1 Hz, 2H), 1.60 (p, *J* = 5.7 Hz, 2H), 1.55 – 1.44 (m, 4H), 1.18 (t, *J* = 7.1 Hz, 3H). ^{13}C NMR (126 MHz, $CDCl_3$) δ 165.8 (d, *J* = 258.3 Hz), 162.2, 150.1, 132.5 (d, *J* = 3.8 Hz), 131.1 (d, *J* = 8.8 Hz), 130.0, 116.3 (d, *J* = 25.2 Hz), 61.1, 30.0, 29.5, 27.5, 27.1, 25.7, 13.8. ^{19}F NMR (471 MHz, $CDCl_3$) δ -102.71. HRMS (ESI) $[M+Na]^+$: calculated for $C_{16}H_{19}FO_5SNa$: 365.0835, found 365.0833.

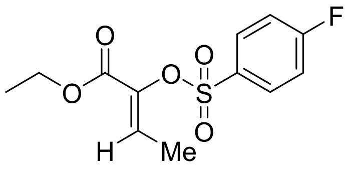

6

Ethyl (*Z*)-2-(((4-fluorophenyl)sulfonyl)oxy)but-2-enoate (6). Flash column chromatography to afford product **6** as a yellow liquid. ^{1}H NMR (500 MHz, $CDCl_3$) δ 8.00 – 7.97 (m, 2H), 7.22 – 7.17 (m, 2H), 6.76 – 6.72 (m, 1H), 4.08 (q, *J* = 7.1 Hz, 2H), 1.75 (d, *J* = 7.3 Hz, 3H), 1.15 (t, *J* = 7.1 Hz, 3H). ^{13}C NMR (126 MHz, $CDCl_3$) δ 165.8 (d, *J* = 258.3 Hz), 161.2, 138.0,

132.4 (d, J = 2.5 Hz), 131.6, 131.1 (d, J = 10.1 Hz), 116.2 (d, J = 25.2 Hz), 61.4, 13.8, 12.2. ^{19}F NMR (471 MHz, $CDCl_3$) δ -102.56. HRMS (ESI) $[M+Na]^+$: calculated for $C_{12}H_{13}FO_5SNa$: 311.0365, found 311.0372.

3 General Procedure of Suzuki Reaction

3.1 General Procedure of the (Hetero)arylation Reactions of α-Arylsulfonyloxyacrylates

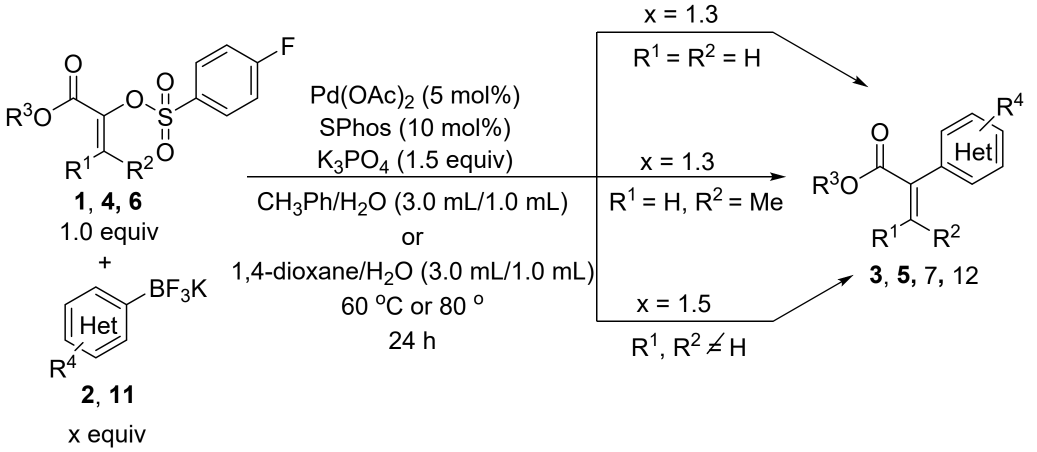

To a reaction tube equipped with a magnetic bar were added α-arylsulfonyloxyacrylate **1, 4** or **6** (0.2 mmol), potassium (hetero)aryltrifluoroborate **2** or **11** (0.26 mmol or 0.3 mmol), SPhos (8.2 mg, 0.02 mmol), K_3PO_4 (63.6 mg, 0.3 mmol), $Pd(OAc)_2$ (2.3 mg, 0.01 mmol). The toluene or 1,4-dioxane (3.0 mL) and H_2O (1.0 mL) were added subsequently. The reaction mixture was stirred at 60 °C or 80 °C for 24 h. The reaction was allowed to cool to room temperature, diluted with water (10 mL) and extracted with ethyl acetate (3 × 10 mL). The organic phase was dried with $MgSO_4$ and concentrated *in vacuo*. The crude product was purified by silica gel column chromatography to afford the product.

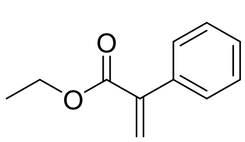

3a

Ethyl 2-phenylacrylate (3a). Flash column chromatography to afford product **3a** as a yellow liquid (25.7 mg, 73% yield). ^{1}H NMR (500 MHz, $CDCl_3$) δ 7.44 – 7.42 (m, 2H), 7.37-7.34 (m, 3H), 6.35 (d, J = 1.3 Hz, 1H), 5.89 (d, J = 1.2 Hz, 1H), 4.30 (q, J = 7.1 Hz, 2H), 1.34 (t, J = 7.1 Hz, 3H). ^{13}C NMR (126 MHz, $CDCl_3$) δ 166.8, 141.6, 136.8, 128.3, 128.0(9), 128.0(5), 126.4, 61.1, 14.2. These data are consistent with the published literature.[3]

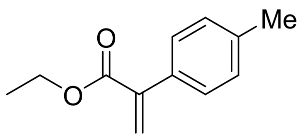

3b

Ethyl 2-(*p*-tolyl)acrylate (3b). Flash column chromatography to afford product **3b** as a colorless liquid (28.2 mg, 74% yield). ^{1}H NMR (500 MHz, $CDCl_3$) δ 7.32-7.31 (m, 2H), 7.17 (d, J = 7.8 Hz, 2H), 6.30 (d, J = 1.3 Hz, 1H), 5.86 (d, J = 1.3 Hz, 1H), 4.29 (q, J = 7.1 Hz, 2H), 2.37 (s, 3H), 1.34 (t, J = 7.1 Hz, 3H). ^{13}C NMR (126 MHz, $CDCl_3$) δ 167.0, 141.4, 138.0, 133.9, 128.8, 128.1, 125.7, 61.0, 21.2, 14.2. These data are consistent with the published literature.[4]

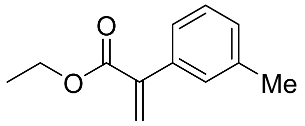

3c

Ethyl 2-(*m*-tolyl)acrylate (3c). Flash column chromatography to afford product **3c** as a colorless liquid (20.9 mg, 55% yield). ^{1}H NMR (500 MHz, $CDCl_3$) δ 7.26 – 7.20 (m, 3H), 7.16 – 7.13 (m, 1H), 6.31 (d, J = 1.3 Hz, 1H), 5.86 (d, J = 1.3 Hz, 1H), 4.29 (q, J = 7.1 Hz, 2H), 2.37 (s, 3H), 1.33 (t, J = 7.1 Hz, 3H). ^{13}C NMR (126 MHz, $CDCl_3$) δ 166.9, 141.7, 137.7, 136.7, 128.9, 128.7, 127.9, 126.2, 125.4, 61.1, 21.4, 14.2.These data are consistent with the published literature.[4]

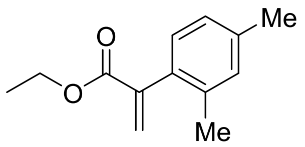

3d

Ethyl 2-(2,4-dimethylphenyl)acrylate (3d). Flash column chromatography to afford product **3d** as a colorless liquid (28.6 mg, 70% yield). ^{1}H NMR (500 MHz, $CDCl_3$) δ 7.04 – 6.99 (m, 3H), 6.47 (d, J = 1.8 Hz, 1H), 5.67 (d, J = 1.8 Hz, 1H), 4.24 (q, J = 7.1 Hz, 2H), 2.33 (s, 3H), 2.17 (s, 3H), 1.28 (t, J = 7.1 Hz, 3H). ^{13}C NMR (126 MHz, $CDCl_3$) δ 166.8, 142.0, 137.8, 135.9, 134.5, 130.7, 129.4, 128.0, 126.3, 60.9, 21.1, 19.8, 14.2. HRMS (ESI) $[M+Na]^+$: calculated for $C_{13}H_{16}O_2Na$: 227.1048, found 227.1049.

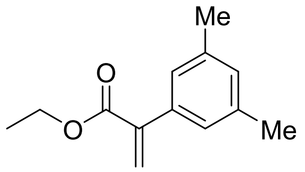

3e

Ethyl 2-(3,5-dimethylphenyl)acrylate (3e). Flash column chromatography to afford product **3e** as a yellow liquid (32.7 mg, 80% yield). ^{1}H NMR (500 MHz, $CDCl_3$) δ 7.01 (d, J = 25.1 Hz, 3H), 6.29 (d, J = 1.4 Hz, 1H), 5.85 (d, J = 1.3 Hz, 1H), 4.29 (q, J = 7.1 Hz, 2H), 2.33 (s, 6H), 1.34 (t, J

= 7.1 Hz, 3H). ^{13}C NMR (126 MHz, $CDCl_3$) δ 167.0, 141.8, 137.6, 136.7, 129.8, 126.1, 125.9, 61.0, 21.3, 14.2. This compound was reported in the published literature.[1b]

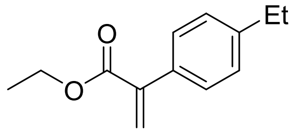

3f

Ethyl 2-(4-ethylphenyl)acrylate (3f). Flash column chromatography to afford product **3f** as a yellow liquid (23.7 mg, 58% yield). ^{1}H NMR (500 MHz, $CDCl_3$) δ 7.38 – 7.34 (m, 2H), 7.20 (d, J = 8.0 Hz, 2H), 6.30 (d, J = 1.3 Hz, 1H), 5.87 (d, J = 1.3 Hz, 1H), 4.30 (q, J = 7.1 Hz, 2H), 2.67 (q, J = 7.7 Hz, 2H), 1.34 (t, J = 7.2 Hz, 3H), 1.25 (t, J = 7.6 Hz, 3H). ^{13}C NMR (126 MHz, $CDCl_3$) δ 167.0, 144.3, 141.4, 134.1, 128.2, 127.6, 125.7, 61.0, 28.6, 15.4, 14.2. This compound was reported in the published literature.[5]

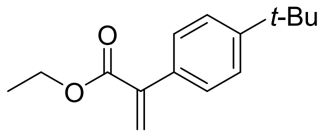

3g

Ethyl 2-(4-(*tert*-butyl)phenyl)acrylate (3g). Flash column chromatography to afford product **3g** as a colorless liquid (38.6 mg, 83% yield). ^{1}H NMR (500 MHz, $CDCl_3$) δ 7.39 (s, 4H), 6.31 (d, J = 1.4 Hz, 1H), 5.89 (d, J = 1.3 Hz, 1H), 4.31 (q, J = 7.2 Hz, 2H), 1.35 (t, J = 7.0 Hz, 3H).1.34 (s, 9H). ^{13}C NMR (126 MHz, $CDCl_3$) δ 167.0, 151.1, 141.3, 133.8, 128.0, 125.7, 125.0, 61.0, 34.6, 31.3, 14.2. This compound was reported in the published literature.[1b]

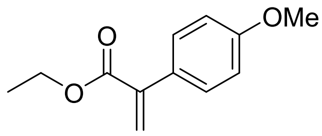

3h

Ethyl 2-(4-methoxyphenyl)acrylate (3h). Flash column chromatography to afford product **3h** as a colorless liquid (24.7 mg, 60% yield). ^{1}H NMR (500 MHz, $CDCl_3$) δ 7.39 – 7.32 (m, 2H), 6.90 – 6.87 (m, 2H), 6.25 (d, J = 1.2 Hz, 1H), 5.82 (d, J = 1.2 Hz, 1H), 4.29 (q, J = 7.1 Hz, 2H), 3.82 (s, 3H), 1.33 (t, J = 7.1 Hz, 3H). ^{13}C NMR (126 MHz, $CDCl_3$) δ 167.1, 159.6, 140.9, 129.5, 129.2, 124.9, 113.5, 61.0, 55.3, 14.2. These data are consistent with the published literature.[6]

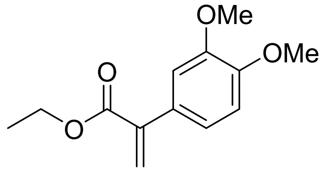

3i

Ethyl 2-(3,4-dimethoxyphenyl)acrylate (3i). Flash column chromatography to afford product **3i** as a yellow liquid (30.2 mg, 64% yield). ^{1}H NMR (500 MHz, $CDCl_3$) δ 7.01-6.99 (m, 2H), 6.85 (d,

J = 6.7 Hz, 1H), 6.26 (d, J = 1.2 Hz, 1H), 5.84 (d, J = 1.3 Hz, 1H), 4.29 (q, J = 7.1 Hz, 2H), 3.89 (s, 6H), 1.34 (t, J = 7.1 Hz, 3H). ^{13}C NMR (126 MHz, $CDCl_3$) δ 167.0, 149.1, 148.4, 141.0, 129.5, 125.2, 120.9, 111.6, 110.7, 61.0, 55.9, 55.8, 14.2. This compound was reported in the published literature.[7]

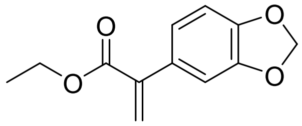

3j

Ethyl 2-(benzo[d][1,3]dioxol-5-yl)acrylate (3j). Flash column chromatography to afford product **3j** as a yellow liquid (33.0 mg, 75% yield). ^{1}H NMR (500 MHz, $CDCl_3$) δ 6.95 – 6.87 (m, 2H), 6.79 (d, J = 8.0 Hz, 1H), 6.25 (s, 1H), 5.97 (s, 2H), 5.81 (s, 1H), 4.28 (q, J = 7.1 Hz, 2H), 1.33 (t, J = 7.1 Hz, 3H). ^{13}C NMR (126 MHz, $CDCl_3$) δ 166.9, 147.6, 147.4, 141.0, 130.8, 125.5, 122.1, 108.9, 107.9, 101.1, 61.1, 14.2. These data are consistent with the published literature.[8]

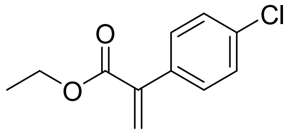

3k

Ethyl 2-(4-chlorophenyl)acrylate (3k). Flash column chromatography to afford product **3k** as a yellow liquid (22.7 mg, 54% yield). ^{1}H NMR (500 MHz, $CDCl_3$) δ 7.39 – 7.30 (m, 4H), 6.37 (d, J = 1.1 Hz, 1H), 5.89 (d, J = 1.1 Hz, 1H), 4.29 (q, J = 7.1 Hz, 2H), 1.33 (t, J = 7.1 Hz, 3H). ^{13}C NMR (126 MHz, $CDCl_3$) δ 166.4, 140.4, 135.2, 134.2, 129.7, 128.3, 126.9, 61.2, 14.2. These data are consistent with the published literature.[4]

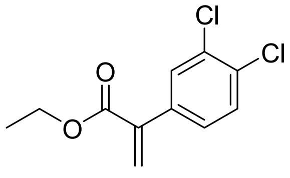

3l

Ethyl 2-(3,4-dichlorophenyl)acrylate (3l). Flash column chromatography to afford product **3l** as a yellow liquid (15.2 mg, 31% yield). ^{1}H NMR (500 MHz, $CDCl_3$) δ 7.54 (d, J = 2.1 Hz, 1H), 7.42 (d, J = 8.4 Hz, 1H), 7.28 – 7.26 (m, 1H), 6.41 (d, J = 0.9 Hz, 1H), 5.92 (d, J = 1.0 Hz, 1H), 4.29 (q, J = 7.1 Hz, 2H), 1.33 (t, J = 7.1 Hz, 3H). ^{13}C NMR (126 MHz, $CDCl_3$) δ 166.0, 139.4, 136.7, 132.3, 132.2, 130.3, 130.0, 128.0 127.7, 61.4, 14.2. These data are consistent with the published literature.[9]

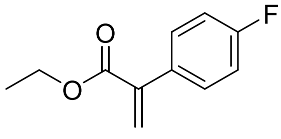

3m

Ethyl 2-(4-fluorophenyl)acrylate (3m). Flash column chromatography to afford product **3m** as a yellow liquid (15.1 mg, 39% yield). ^{1}H NMR (500 MHz, $CDCl_3$) δ 7.41 – 7.37 (m, 2H), 7.06 – 7.02 (m, 2H), 6.35 (d, J = 1.2 Hz, 1H), 5.86 (d, J = 1.2 Hz, 1H), 4.29 (q, J = 7.1 Hz, 2H), 1.34 (d, J = 7.1 Hz, 3H). ^{13}C NMR (126 MHz, $CDCl_3$) δ 166.6, 162.7 (d, J = 252 Hz), 140.5, 132.8 (d, J = 2.5 Hz), 130.1 (d, J = 8.8 Hz), 126.5, 115.1 (d, J = 21.4 Hz), 61.2, 14.2. ^{19}F NMR (471 MHz, $CDCl_3$) δ -113.95. These data are consistent with the published literature.[6]

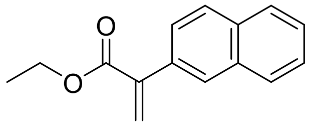

3n

Ethyl 2-(naphthalen-2-yl)acrylate (3n). Flash column chromatography to afford product **3n** as a yellow liquid (23.5 mg, 52% yield). ^{1}H NMR (500 MHz, $CDCl_3$) δ 7.93 (d, J = 1.8 Hz, 1H), 7.88 – 7.80 (m, 3H), 7.59-7.54 (m, 1H), 7.50-7.48 (m, 2H), 6.44 (d, J = 1.2 Hz, 1H), 6.02 (d, J = 1.2 Hz, 1H), 4.34 (q, J = 7.1 Hz, 2H), 1.39 – 1.34 (t, J = 7.5 Hz, 3H). ^{13}C NMR (126 MHz, $CDCl_3$) δ 166.9, 141.6, 134.2, 133.1, 132.9, 128.3, 127.6, 127.5, 127.4, 126.7, 126.3, 126.2, 126.1, 61.2, 14.2. These data are consistent with the published literature.[10]

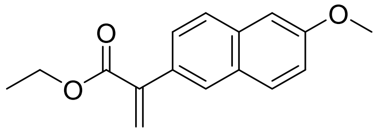

3o

Ethyl 2-(6-methoxynaphthalen-2-yl)acrylate (3o). Flash column chromatography to afford product **3o** as a white solid (31.3 mg, 61% yield). ^{1}H NMR (500 MHz, $CDCl_3$) δ 7.85 (d, J = 1.8 Hz, 1H), 7.75 – 7.69 (m, 2H), 7.53 – 7.50 (m, 1H), 7.17 – 7.10 (m, 2H), 6.38 (s, 1H), 5.98 (s, 1H), 4.33 (q, J = 7.2 Hz, 2H), 3.93 (s, 3H), 1.35 (t, J = 7.1 Hz, 3H). ^{13}C NMR (126 MHz, $CDCl_3$) δ 167.1, 158.0, 141.5, 134.2, 131.9, 129.8, 128.5, 127.3, 126.6, 126.4, 125.9, 119.0, 105.5, 61.1, 55.3, 14.2. These data are consistent with the published literature.[4]

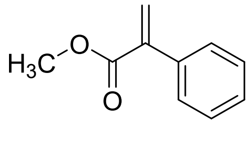

3p

Methyl 2-phenylacrylate (3p). Flash column chromatography to afford product **3p** as a colorless liquid (27.2 mg, 84% yield). ^{1}H NMR (500 MHz, $CDCl_3$) δ 7.44 – 7.40 (m, 2H), 7.39 – 7.34 (m,

3H), 6.37 (s, 1H), 5.90 (s, 1H), 3.83 (s, 3H). ^{13}C NMR (126 MHz, $CDCl_3$) δ 167.3, 141.3, 136.7, 128.3, 128.2, 128.1, 127.0, 52.2. These data are consistent with the published literature.[3]

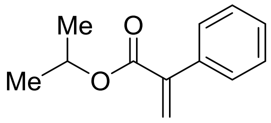

3q

***Iso*-propyl 2-phenylacrylate (3q).** Flash column chromatography to afford product **3q** as a yellow liquid (20.2 mg, 53% yield). ^{1}H NMR (500 MHz, $CDCl_3$) δ 7.48 – 7.42 (m, 2H), 7.41 – 7.32 (m, 3H), 6.33 (s, 1H), 5.89 (s, 1H), 5.23 – 5.13 (m, 1H), 1.34 (d, J = 6.3 Hz, 6H). ^{13}C NMR (126 MHz, $CDCl_3$) δ 166.4, 141.9, 136.8, 128.3, 128.2, 126.0, 68.6, 21.8. These data are consistent with the published literature.[3]

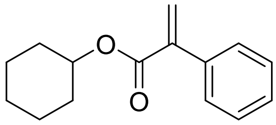

3r

Cyclohexyl 2-phenylacrylate (3r). Flash column chromatography to afford product **3r** as a colorless liquid (30.4 mg, 66% yield). ^{1}H NMR (500 MHz, $CDCl_3$) δ 7.46 – 7.40 (m, 2H), 7.39 – 7.30 (m, 3H), 6.33 (d, J = 1.2 Hz, 1H), 5.87 (t, J = 1.0 Hz, 1H), 4.98 – 4.92 (m, 1H), 1.94 – 1.87 (m, 2H), 1.77 – 1.69 (m, 2H), 1.55 – 1.51 (m, 2H), 1.45 – 1.37 (m, 2H), 1.36 – 1.23 (m, 2H). ^{13}C NMR (126 MHz, $CDCl_3$) δ 166.2, 141.9, 136.9, 128.3, 128.0, 127.9, 126.0, 73.29, 31.5, 25.4, 23.6. These data are consistent with the published literature.[15]

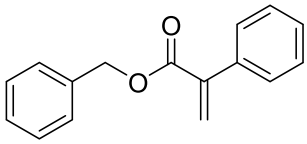

3s

Benzyl 2-phenylacrylate (3s). Flash column chromatography to afford product **3s** as a yellow liquid (33.4 mg, 70% yield). ^{1}H NMR (500 MHz, $CDCl_3$) δ 7.44 – 7.42 (m, 2H), 7.39 – 7.33 (m, 8H), 6.40 (s, 1H), 5.92 (s, 1H), 5.28 (s, 2H). ^{13}C NMR (126 MHz, $CDCl_3$) δ 166.6, 141.2, 136.6, 135.9, 128.7, 128.5, 128.4, 128.3, 128.2, 127.1, 66.8. These data are consistent with the published literature.[3]

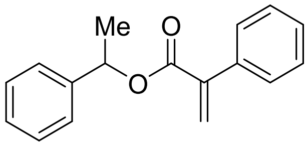

3t

1-Phenylethyl 2-phenylacrylate (3t). Flash column chromatography to afford product **3t** as a colorless liquid (31.7 mg, 63% yield). ^{1}H NMR (500 MHz, $CDCl_3$) δ 7.44 – 7.31 (m, 10H), 6.41 (s, 1H), 6.06 (q, J = 6.6 Hz, 1H), 5.92 (s, 1H), 1.63 (d, J = 6.6 Hz, 3H). ^{13}C NMR (126 MHz, $CDCl_3$)

δ 165.9, 141.6, 141.5, 136.7, 128.5, 128.4, 128.3, 128.1, 128.0, 127.9, 126.6, 126.0, 22.3. HRMS (ESI) $[M+Na]^+$: calculated for $C_{17}H_{16}O_2Na$: 275.1048, found 275.1054.

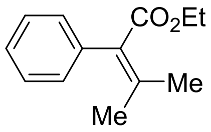

5a

Ethyl 3-methyl-2-phenylbut-2-enoate (5a). Flash column chromatography to afford product **5a** as a yellow liquid (25.3 mg, 62% yield). ^{1}H NMR (500 MHz, $CDCl_3$) δ 7.36 – 7.33 (m, 2H), 7.29 – 7.27 (m, 1H), 7.21 – 7.19 (m, 2H), 4.17 (q, *J* = 7.1 Hz, 2H), 2.12 (s, 3H), 1.70 (s, 3H), 1.22 (t, *J* = 7.1 Hz, 3H). ^{13}C NMR (126 MHz, $CDCl_3$) δ 168.8, 143.9, 138.1, 130.3, 129.4, 128.0, 126.9, 60.4, 23.1, 22.5, 14.2. These data are consistent with the published literature.[12]

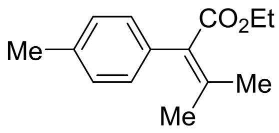

5b

Ethyl 3-methyl-2-(*p*-tolyl)but-2-enoate (5b). Flash column chromatography to afford product **5b** as a colorless liquid (32.3 mg, 74% yield). ^{1}H NMR (500 MHz, $CDCl_3$) δ 7.16 – 7.11 (m, 2H), 7.09 – 7.06 (m, 2H), 4.16 (q, *J* = 7.1 Hz, 2H), 2.35 (s, 3H), 2.09 (s, 3H), 1.70 (s, 3H), 1.22 (t, *J* = 7.1 Hz, 3H). ^{13}C NMR (126 MHz, $CDCl_3$) δ 169.0, 143.2, 136.5, 135.0, 130.2, 129.3, 128.8, 60.4, 22.9, 22.5, 21.2, 14.2. These data are consistent with the published literature.[4]

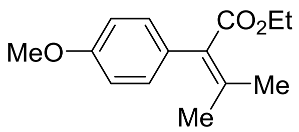

5c

Ethyl 2-(4-methoxyphenyl)-3-methylbut-2-enoate (5c). Flash column chromatography to afford product **5c** as a colorless liquid (29.9 mg, 64% yield). ^{1}H NMR (500 MHz, $CDCl_3$) δ 7.12 – 7.09 (m, 2H), 6.88 – 6.85 (m, 2H), 4.16 (q, *J* = 7.1 Hz, 2H), 3.81 (s, 3H), 2.07 (s, 3H), 1.70 (s, 3H), 1.22 (t, *J* = 7.1 Hz, 3H). ^{13}C NMR (126 MHz, $CDCl_3$) δ 169.1, 158.5, 143.1, 130.5, 130.3, 129.9, 113.5, 60.4, 55.2, 22.9, 22.5, 14.2. HRMS (ESI) $[M+Na]^+$: calculated for $C_{14}H_{18}O_3Na$: 257.1154, found 257.1142.

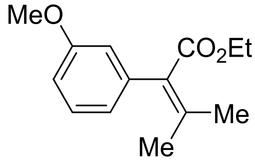

5d

Ethyl 2-(3-methoxyphenyl)-3-methylbut-2-enoate (5d). Flash column chromatography to afford product **5d** as a colorless liquid (30.5 mg, 65% yield). ^{1}H NMR (500 MHz, $CDCl_3$) δ 7.31 – 7.27

(m, 1H), 6.87 – 6.74 (m, 3H), 4.20 (q, J = 7.1 Hz, 2H), 3.83 (s, 3H), 2.13 (s, 3H), 1.73 (s, 3H), 1.25 (t, J = 7.1 Hz, 3H). ^{13}C NMR (126 MHz, $CDCl_3$) δ 168.7, 159.3, 143.9, 139.4, 130.2, 129.0, 121.9, 114.9, 112.5, 60.4, 55.2, 23.1, 22.5, 14.1. These data are consistent with the published literature.[4]

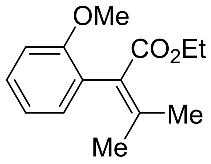

5e

Ethyl 2-(2-methoxyphenyl)-3-methylbut-2-enoate (5e). Flash column chromatography to afford product **5e** as a colorless liquid (27.6 mg, 59% yield). ^{1}H NMR (500 MHz, $CDCl_3$) δ 7.29 – 7.27 (m, 1H), 7.12 – 7.10 (m, 1H), 6.97 – 6.93 (m, 1H), 6.88 (d, J = 1.1 Hz, 1H), 4.18 – 4.13 (q, J = 7.5 Hz, 2H), 3.78 (s, 3H), 2.20 (s, 3H), 1.74 (s, 3H), 1.18 (t, J = 7.1 Hz, 3H). ^{13}C NMR (126 MHz, $CDCl_3$) δ 168.4, 157.1, 146.8, 131.6, 128.5, 127.6, 126.5, 120.2, 110.5, 59.9, 55.4, 23.9, 22.5, 14.2. These data are consistent with the published literature.[4]

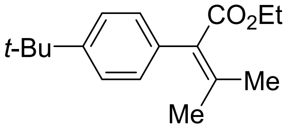

5f

Ethyl 2-(4-(*tert*-butyl)phenyl)-3-methylbut-2-enoate (5f). Flash column chromatography to afford product **5f** as a yellow liquid (48.4 mg, 93% yield). ^{1}H NMR (500 MHz, $CDCl_3$) δ 7.36 – 7.31 (m, 2H), 7.12 (d, J = 8.3 Hz, 2H), 4.17 (q, J = 7.1 Hz, 2H), 2.07 (s, 3H), 1.71 (s, 3H), 1.32 (s, 9H), 1.24 (t, J = 7.1 Hz, 3H). ^{13}C NMR (126 MHz, $CDCl_3$) δ 169.2, 149.7, 142.5, 134.8, 130.3, 128.9, 124.9, 60.4, 34.5, 31.3, 22.9, 22.6, 14.2. HRMS (ESI) $[M+H]^+$: calculated for $C_{17}H_{24}O_2$: 261.1852, found 261.1855.

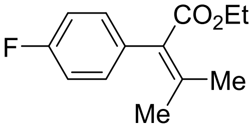

5g

Ethyl 2-(4-fluorophenyl)-3-methylbut-2-enoate (5g). Flash column chromatography to afford product **5g** as a yellow liquid (27.1 mg, 61% yield). ^{1}H NMR (500 MHz, $CDCl_3$) δ 7.16 – 7.11 (m, 2H), 7.03 – 6.99 (m, 2H), 4.15 (q, J = 7.1 Hz, 2H), 2.11 (s, 3H), 1.67 (s, 3H), 1.21 (t, J = 7.1 Hz, 3H). ^{13}C NMR (126 MHz, $CDCl_3$) δ 168.5, 161.8 (d, J = 245.7 Hz), 144.9, 134.1 (d, J = 2.5 Hz), 131.1 (d, J = 7.6 Hz), 129.3, 115.1 (d, J = 21.5 Hz), 60.4, 23.2, 22.5, 14.2. ^{19}F NMR (471 MHz, CDCl3) δ -115.58. HRMS (ESI) $[M+H]^+$: calculated for $C_{13}H_{15}FO_2$: 223.1134, found 223.1137.

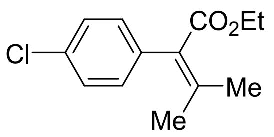

5h

Ethyl 2-(4-chlorophenyl)-3-methylbut-2-enoate (5h). Flash column chromatography to afford product **5h** as a colorless liquid (24.3 mg, 51% yield). ^{1}H NMR (500 MHz, $CDCl_3$) δ 7.33 – 7.29 (m, 2H), 7.14 – 7.10 (m, 2H), 4.16 (q, J = 7.1 Hz, 2H), 2.13 (s, 3H), 1.70 (s, 3H), 1.21 (t, J = 7.1 Hz, 3H). ^{13}C NMR (126 MHz, $CDCl_3$) δ 168.2, 145.5, 132.6, 130.8, 129.1, 128.2, 60.5, 23.3, 22.6, 14.2.These data are consistent with the published literature.[4]

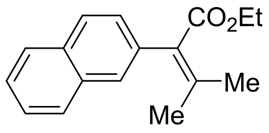

5i

Ethyl 3-methyl-2-(naphthalen-2-yl)but-2-enoate (5i). Flash column chromatography to afford product **5i** as a yellow liquid (30.9 mg, 61% yield). ^{1}H NMR (500 MHz, $CDCl_3$) δ 7.84 – 7.75 (m, 3H), 7.65 (d, J = 1.7 Hz, 1H), 7.51 – 7.42 (m, 2H), 7.32 (d, J = 1.7 Hz, 1H), 4.17 (q, J = 7.1 Hz, 2H), 2.17 (s, 3H), 1.74 (s, 3H), 1.20 (t, J = 7.1 Hz, 3H). ^{13}C NMR (126 MHz, $CDCl_3$) δ 168.7, 144.7, 135.6, 133.2, 132.3, 130.2, 128.2, 127.9, 127.8, 127.6, 127.5, 125.9, 125.8, 60.4, 23.3, 22.6, 14.2. These data are consistent with the published literature.[13]

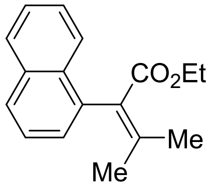

5j

Ethyl 3-methyl-2-(naphthalen-1-yl)but-2-enoate (5j). Flash column chromatography to afford product **5j** as a colorless liquid (31.0 mg, 61% yield). ^{1}H NMR (500 MHz, $CDCl_3$) δ 7.86 – 7.79 (m, 3H), 7.48 – 7.45 (m, 3H), 7.31 – 7.29 (m, 1H), 4.10 – 4.05 (m, 2H), 2.30 (s, 3H), 1.54 (s, 3H), 1.07 (t, J = 7.1 Hz, 3H). ^{13}C NMR (126 MHz, $CDCl_3$) δ 168.3, 147.9, 136.2, 133.6, 132.2, 128.2, 127.9, 127.5, 127.3, 125.9, 125.6, 125.5, 125.4, 60.2, 23.8, 22.4, 14.1. HRMS (ESI) $[M+Na]^+$: calculated for $C_{17}H_{18}O_2Na$: 277.1024, found 277.1203.

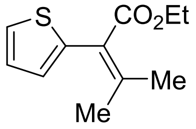

5k

Ethyl 3-methyl-2-(thiophen-2-yl)but-2-enoate (5k). Flash column chromatography to afford product **5k** as a yellow liquid (19.3 mg, 46% yield). ^{1}H NMR (500 MHz, $CDCl_3$) δ 7.29 – 7.27 (m, 1H), 7.00 – 6.98 (m, 1H), 6.87 – 6.83 (m, 1H), 4.21 (q, J = 7.1 Hz, 2H), 2.09 (s, 3H), 1.86 (s, 3H),

1.26 (t, J = 7.1 Hz, 3H). ^{13}C NMR (126 MHz, $CDCl_3$) δ 168.2, 146.0, 138.5, 127.2, 126.5, 125.5, 123.6, 60.7, 23.3, 22.9, 14.1. HRMS (ESI) $[M+Na]^+$: calculated for $C_{11}H_{14}O_2SNa$: 233.0612, found 233.0613.

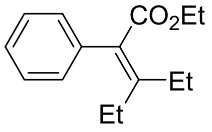

5l

Ethyl 3-ethyl-2-phenylpent-2-enoate (5l). Flash column chromatography to afford product **5l** as a colorless liquid (32.5 mg, 70% yield). ^{1}H NMR (500 MHz, $CDCl_3$) δ 7.36 – 7.32 (m, 2H), 7.31 – 7.26 (m, 1H), 7.23 – 7.19 (m, 2H), 4.15 (q, J = 7.1 Hz, 2H), 2.45 (q, J = 7.5 Hz, 2H), 2.00 (q, J = 7.5 Hz, 2H), 1.22 (t, J = 7.1 Hz, 3H), 1.16 (t, J = 7.5 Hz, 3H), 0.95 (t, J = 7.5 Hz, 3H). ^{13}C NMR (126 MHz, $CDCl_3$) δ 168.8, 153.5, 137.9, 129.9, 129.3, 128.0, 126.9, 60.3, 25.6, 14.1, 13.3, 12.7. HRMS (ESI) $[M+Na]^+$: calculated for $C_{15}H_{20}O_2Na$: 255.1361, found 255.1263.

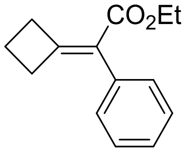

5m

Ethyl 2-cyclobutylidene-2-phenylacetate (5m). Flash column chromatography to afford product **5m** as a yellow liquid (21.6 mg, 50% yield). ^{1}H NMR (500 MHz, $CDCl_3$) δ 7.36 – 7.32 (m, 2H), 7.28-7.23 (m, 3H), 4.22 (q, J = 7.1 Hz, 2H), 3.29 – 3.24 (m, 2H), 2.82 – 2.78 (m, 2H), 2.10-2.04 (m, 2H), 1.59 (s, 3H), 1.29 (t, J = 7.1 Hz, 4H). ^{13}C NMR (126 MHz, $CDCl_3$) δ 166.8, 162.4, 135.7, 129.2, 127.9, 126.9, 125.9, 60.2, 34.4, 32.5, 17.2, 14.4. HRMS (ESI) $[M+Na]^+$: calculated for $C_{14}H_{16}O_2Na$: 239.1048, found 239.1044.

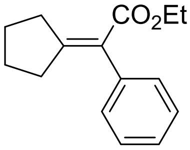

5n

Ethyl 2-cyclopentylidene-2-phenylacetate (5n). Flash column chromatography to afford product **5n** as a yellow liquid (25.3 mg, 55% yield). ^{1}H NMR (500 MHz, $CDCl_3$) δ 7.37 – 7.34 (m, 2H), 7.29 – 7.27 (m, 1H), 7.19 – 7.17 (m, 2H), 4.20 – 4.16 (q, J = 7.0 Hz , 2H), 2.91 – 2.88 (m, 2H), 2.24 – 2.21 (m, 2H), 1.82-1.76 (m, 2H), 1.63-1.58 (m, 3H), 1.24 (t, J = 7.1 Hz, 3H). ^{13}C NMR (126 MHz, $CDCl_3$) δ 167.5, 162.8, 139.1, 129.2, 127.9, 126.7, 125.8, 60.1, 35.2, 34.0, 26.8, 25.8, 14.3. HRMS (ESI) $[M+Na]^+$: calculated for $C_{15}H_{18}O_2Na$: 253.1204, found 253.1205.

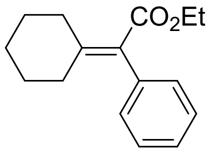

5o

Ethyl 2-cyclohexylidene-2-phenylacetate (5o). Flash column chromatography to afford product **5o** as a light yellow liquid (24.4 mg, 50% yield). ^{1}H NMR (500 MHz, $CDCl_3$) δ 7.36 – 7.33 (m,

2H), 7.29 – 7.27 (m, 1H), 7.24 – 7.20 (m, 2H), 4.20 – 4.16 (q, J = 7.0 Hz, 2H), 2.55 – 2.53 (m, 2H), 2.12 – 2.09 (m, 2H), 1.74 – 1.70 (m, 2H), 1.64 – 1.55 (m, 4H), 1.24 (d, J = 7.1 Hz, 3H). ^{13}C NMR (126 MHz, $CDCl_3$) δ 169.2, 148.9, 137.4, 129.4, 128.1, 127.7, 126.9, 60.4, 32.6, 32.0, 28.2(5), 28.2(2), 26.3, 14.1. These data are consistent with the published literature.[14]

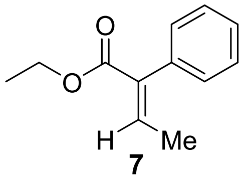

7

Ethyl (*E*)-2-phenylbut-2-enoate (7). Flash column chromatography to afford product **7** as a light yellow liquid (30.8 mg, 81% yield). ^{1}H NMR (500 MHz, $CDCl_3$) δ 7.39 – 7.35 (m, 2H), 7.33 – 7.29 (m, 1H), 7.20 – 7.14 (m, 3H), 4.21 (q, J = 7.1 Hz, 2H), 1.75 (d, J = 7.3 Hz, 3H), 1.26 (t, J = 7.1 Hz, 3H). ^{13}C NMR (126 MHz, $CDCl_3$) δ 167.1, 139.6, 135.1, 134.9, 129.8, 127.9, 127.3, 60.7, 15.4, 14.2. These data are consistent with the published literature.[15]

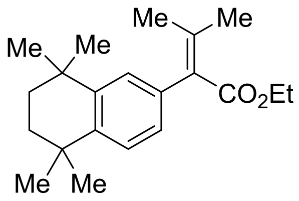

12

Ethyl 3-methyl-2-(5,5,8,8-tetramethyl-5,6,7,8-tetrahydronaphthalen-2-yl)but-2-enoate (12). Flash column chromatography to afford product **12** as a colorless liquid (45.9 mg, 73% yield). ^{1}H NMR (500 MHz,$CDCl_3$) δ 7.23 (d, J = 8.1 Hz, 1H), 7.12 (d, J = 1.9 Hz, 1H), 6.98 – 6.94 (m, 1H), 4.19 (q, J = 7.1 Hz, 2H), 2.04 (s, 3H), 1.72 (s, 3H), 1.68 (s, 4H), 1.28 (s, 6H), 1.26 (s, 6H), 1.25 (t, J = 7.2 Hz, 3H). ^{13}C NMR (126 MHz, $CDCl_3$) δ 169.4, 144.2, 143.3, 141.4, 134.4, 130.8, 127.6, 126.4, 125.9, 60.4, 35.1(2), 35.1(0), 34.2, 34.0, 31.8, 22.7, 22.6, 14.2. These data are consistent with the published literature.[16]

3.2 General Procedure of the Alkylation Reactions of α-Arylsulfonyloxyacrylates

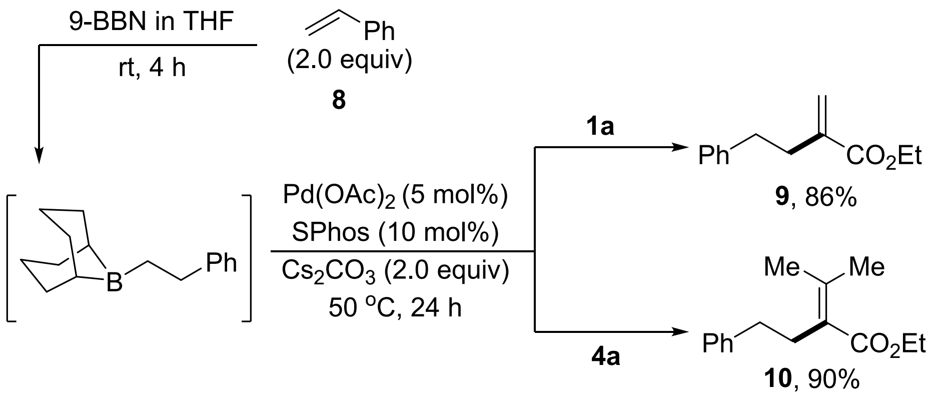

One Schlenk tube equipped with a magnetic stir bar was charged with styrene **8** (208 mg, 2 mmol, 1.0 equiv) and a solution of 9-BBN in THF (4 mL, 0.5 M, 1.0 equiv), stirred for 4 h at room temperature to afford a solution of *B*-phenylethyl-9-BBN in THF. Another Schlenk tube equipped with a magnetic stir bar was charged with **1a** (109.6 mg, 0.4 mmol, 1.0 equiv) or **4a** (120.8 mg, 0.4 mmol, 1.0 equiv), $Pd(OAc)_2$ (4.5 mg, 0.02 mmol), SPhos (16.4 mg, 0.04 mmol), Cs_2CO_3 (260.8 mg, 0.8 mmol, 2.0 equiv) and the solution of *B*-phenylethyl-9-BBN in THF (1.6 mL, prepared above). The mixture was stirred at 50 °C for 24 h. After completed, the reaction mixture was cooled down to room temperature, diluted with ethyl acetate (2 mL), and pushed through a plug of silica gel with ethyl acetate. The filtrate was concentrated under reduced pressure. The residue was chromatographed on silica gel column to give the target product **9** (70.2 mg, 86% yield) or **10** (83.6 mg, 90% yield).

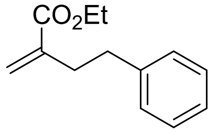

9

Ethyl 2-methylene-4-phenylbutanoate (9). Flash column chromatography to afford product **9** as a yellow liquid (35.1 mg, 86% yield). ^{1}H NMR (500 MHz, $CDCl_3$) δ 7.34 – 7.30 (m, 2H), 7.25 – 7.21 (m, 3H), 6.19 (d, *J* = 1.4 Hz, 1H), 5.53 (q, *J* = 1.3 Hz, 1H), 4.26 (q, *J* = 7.2 Hz, 2H), 2.83 (d, *J* = 8.4 Hz, 2H), 2.70 – 2.64 (m, 2H), 1.35 (t, *J* = 7.1 Hz, 3H). ^{13}C NMR (126 MHz, $CDCl_3$) δ 167.1, 141.4, 140.1, 128.5, 128.3, 125.9, 125.1, 60.6, 34.9, 33.9, 14.2. These data are consistent with the published literature.[17]

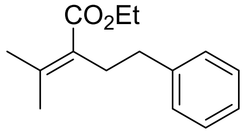

10

Ethyl 3-methyl-2-phenethylbut-2-enoate (10). Flash column chromatography to afford product **10** as a colorless liquid (41.8 mg, 90% yield). ^{1}H NMR (500 MHz, $CDCl_3$) δ 7.31 – 7.27 (m, 2H), 7.22 – 7.19 (m, 3H), 4.21 (q, *J* = 7.1 Hz, 2H), 2.73 – 2.69 (m, 2H), 2.63 – 2.57 (m, 2H), 2.00 (s, 3H), 1.73 (s, 3H), 1.32 (d, *J* = 7.1 Hz, 3H). ^{13}C NMR (126 MHz, $CDCl_3$) δ 169.5, 143.1, 141.8,

128.5, 128.2, 127.0, 125.8, 59.9, 35.3, 32.2, 22.9, 21.7, 14.3. HRMS (ESI) $[M+Na]^+$: calculated for $C_{15}H_{20}O_2Na$: 255.1361, found 255.1360.

4 References

1. (*a*) J. Zhu, Y. Yuan, S. Wang and Z.-J. Yao, Synthesis of 2,3-Dialkylated Tartaric Acid Esters via Visible Light Photoredox-Catalyzed Reductive Dimerization of α-Ketoesters, *ACS Omega*, 2017, **2**, 4665-4677; (*b*) C. Feng, D. Feng and T.-P. Loh, Rhodium(III)-catalyzed C–H allylation of electron-deficient alkenes with allyl acetates, *Chem. Commun.*, 2015, **51**, 342-345; (*c*) X. del Corte, A. López-Francés, E. Martínez de Marigorta, F. Palacios and J. Vicario, Stereo- and Regioselective [3+3] Annulation Reaction Catalyzed by Ytterbium: Synthesis of Bicyclic 1,4-Dihydropyridines, *Adv. Synth. Catal.*, 2021, **363**, 4761-4769.
2. (*a*) L. Zhang, Y. Fang, X. Jin, H. Xu, R. Li, H. Wu, B. Chen, Y. Zhu, Y. Yang and Z. Tian, Pd-Catalysed Suzuki coupling of α-bromoethenylphosphonates with organotrifluoroborates: a general protocol for the synthesis of terminal α-substituted vinylphosphonates, *Org. Biomol. Chem.*, 2017, **15**, 8985-8989; (*b*) A. J. J. Lennox and G. C. Lloyd-Jones, Preparation of Organotrifluoroborate Salts: Precipitation-Driven Equilibrium under Non-Etching Conditions, *Angew. Chem. Int. Ed.*, 2012, **51**, 9385-9388.
3. C. Peng, G. Yan, Y. Wang, Y. Jiang, Y. Zhang and J. Wang, Palladium-Catalyzed Coupling Reaction of α-Diazocarbonyl Compounds with Aromatic Boronic Acids or Halides, *Synthesis*, 2010, 4154-4168.
4. J. Barluenga, M. Tomás-Gamasa, F. Aznar and C. Valdés, Synthesis of 2-Arylacrylates from Pyruvate by Tosylhydrazide-Promoted Pd-Catalyzed Coupling with Aryl Halides, *Chem. Eur. J.*, 2010, **16**, 12801-12803.
5. S. Watanabe, T. Fujita, M. Sakamoto, H. Endo and T. Kitazume, Fluorination of aromatic α-hydroxyesters with N,N-diethyl-1,1,2,3,3,3-hexafluoropropaneamine, *J. Fluorine Chem.*, 1990, **47**, 187-192.
6. F. Berthiol, H. Doucet and M. Santelli, Synthesis of Polysubstituted Alkenes by Heck Vinylation or Suzuki Cross-Coupling Reactions in the Presence of a Tetraphosphane-Palladium Catalyst, *Eur. J. Org. Chem.*, 2003, 1091-1096.
7. D. Yi, Q. Fu, S.-Y. Chen, M. Gao, L. Yang, Z.-J. Zhang, W. Liang, Q. Zhang, J.-X. Ji and W. Wei, Copper-catalyzed direct hydroxyphosphorylation of electron-deficient alkenes with H-phosphine oxides and dioxygen, *Tetrahedron Lett.*, 2017, **58**, 2058-2061.
8. R. J. Sullivan, G. P. R. Freure and S. G. Newman, Overcoming Scope Limitations in Cross-Coupling of Diazo Nucleophiles by Manipulating Catalyst Speciation and Using Flow Diazo Generation, *ACS Catal.*, 2019, **9**, 5623–5630.
9. Q. Zhao, J. Wang, T. Besset, X. Pannecoucke, J.-P. Bouillon and T. Poisson, Palladium-catalyzed synthesis of 3-trifluoromethylated 1,3-dienes from acrylate derivatives and BTP, *Tetrahedron*, 2018, **74**, 6033-6040.
10. A. Gualandi, D. Mazzarella, A. Ortega-Martínez, L. Mengozzi, F. Calcinelli, E. Matteucci, F. Monti, N. Armaroli, L. Sambri and P. G. Cozzi, Photocatalytic

Radical Alkylation of Electrophilic Olefins by Benzylic and Alkylic Zinc-Sulfinates, *ACS Catal.*, 2017, **7**, 5357–5362.

11. D. Yang, L. Liu, D.-L. Wang, Y. Lu, X.-L. Zhao and Y. Liu, Novel multi-dentate phosphines for Pd-catalyzed alkoxycarbonylation of alkynes promoted by H_2O additive, *J. Catal.*, 2019, **371**, 236-244.
12. T. Tsuda, T. Yoshida and T. Saegusa, Palladium(0)-Catalyzed Coupling Reaction of Lithium (α-Carbalkoxyviny1)cuprates with Organic Halides, *J. Org. Chem.*, 1988, **53**, 607-610.
13. A. Mital, R. Lad, A. Thakur, V. S. Negi and U. Ramachandran, Synthesis of novel 2-substituted 1,4-naphthoquinones using Heck reaction in 'green' reaction media, *ARKIVOC*, 2006, 99-106.
14. L. Zhou, Y. Liu, Y. Zhang and J. Wang, Pd-catalyzed coupling of β-hydroxy α-diazocarbonyl compounds with aryl iodides: a migratory insertion/β-hydroxy elimination sequence, *Chem. Commun.*, 2011, **47**, 3622-3624.
15. M. V. Popescu, A. Mekereeya, J. V. Alegre-Requena, R. S. Paton and M. D. Smith, Visible-Light-Mediated Heterocycle Functionalization via Geometrically Interrupted [2+2] Cycloaddition, *Angew. Chem. Int. Ed.*, 2020, **59**, 23020-23024.
16. M. I. Dawson, L. Jong, P. D. Hobbs, J. F. Cameron, W.-R. Chao, M. Pfahl, M.-O. Lee, B. Shroot and M. Pfahl, Conformational Effects on Retinoid Receptor Selectivity. 2. Effects of Retinoid Bridging Group on Retinoid X Receptor Activity and Selectivity, *J. Med. Chem.*, 1995, **38**, 3368–3383.
17. J. Zhang, Y. Li, R. Xu and Y. Chen, Donor–Acceptor Complex Enables Alkoxyl Radical Generation for Metal-Free $C(sp^3)$–$C(sp^3)$ Cleavage and Allylation/Alkenylation, *Angew. Chem. Int. Ed.*, 2017, **56,** 12619-12623.

5 NMR Spectra of New Compounds

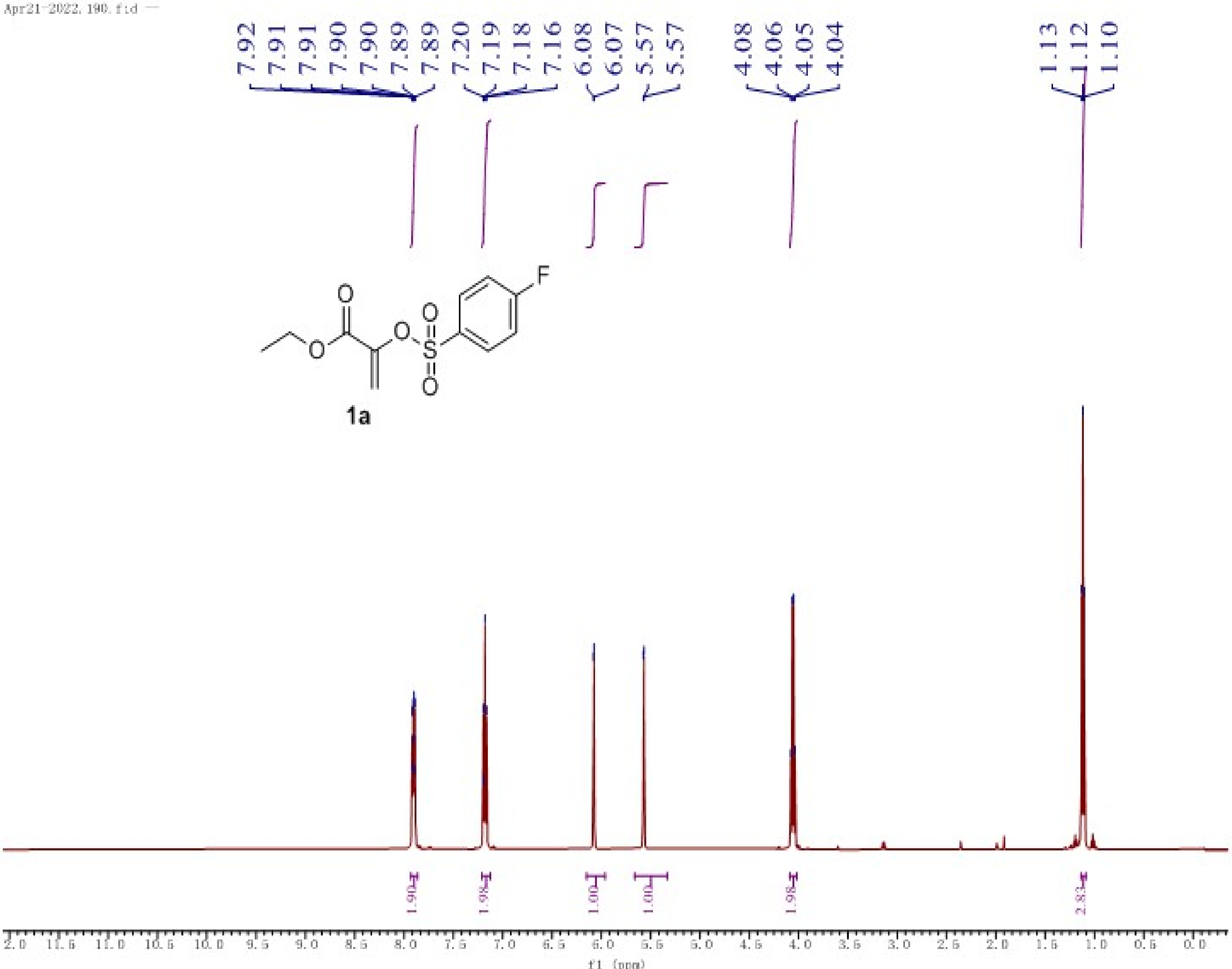
Apr21-2022.190.fid
7.92
7.91
7.91
7.90
7.90
7.89
7.89
7.20
7.19
7.18
7.16
6.08
6.07
5.57
5.57
4.08
4.06
4.05
4.04
1.13
1.12
1.10
F
O
O
O
S
O
O
1a
1.90
1.98
1.00
1.00
1.98
2.83
2.0
11.5
11.0
10.5
10.0
9.5
9.0
8.5
8.0
7.5
7.0
6.5
6.0
5.5
5.0
4.5
4.0
3.5
3.0
2.5
2.0
1.5
1.0
0.5
0.0
f1 (ppm)

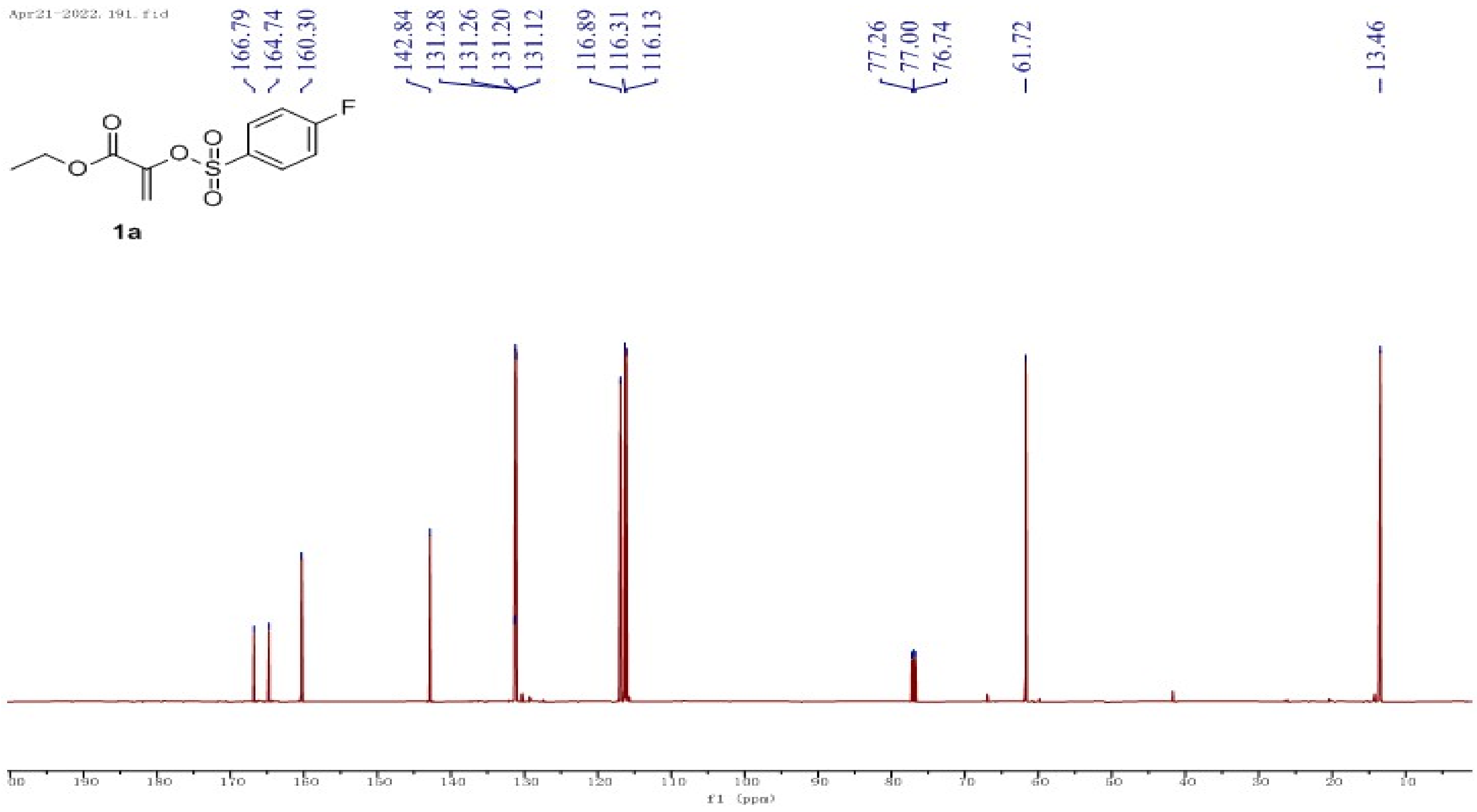
Apr21-2022.191.fid
166.79
164.74
160.30
142.84
131.28
131.26
131.20
131.12
116.89
116.31
116.13
77.26
77.00
76.74
61.72
13.46
F
1a
f1 (ppm)

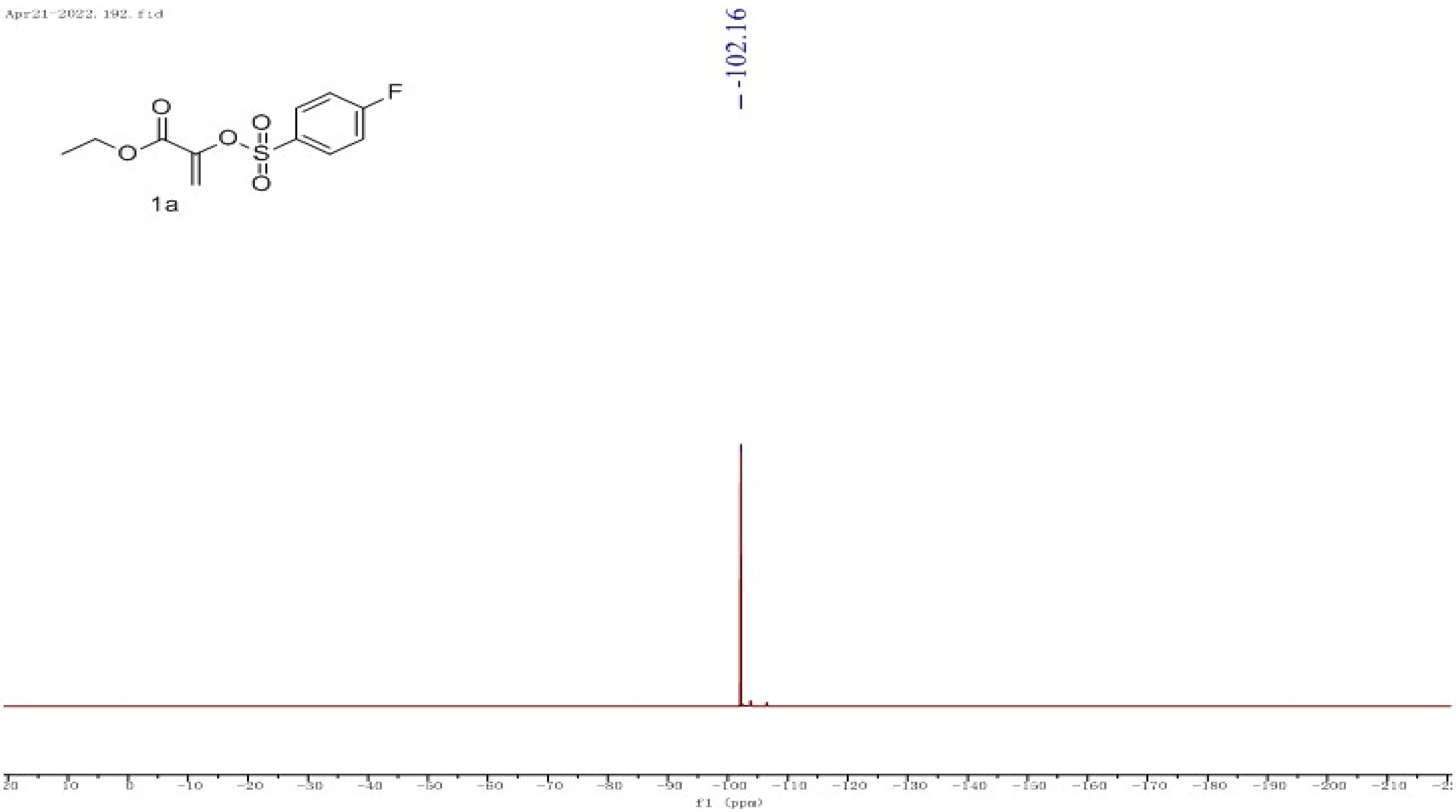

Apr21-2022.192.fid
-102.16
1a
f1 (ppm)

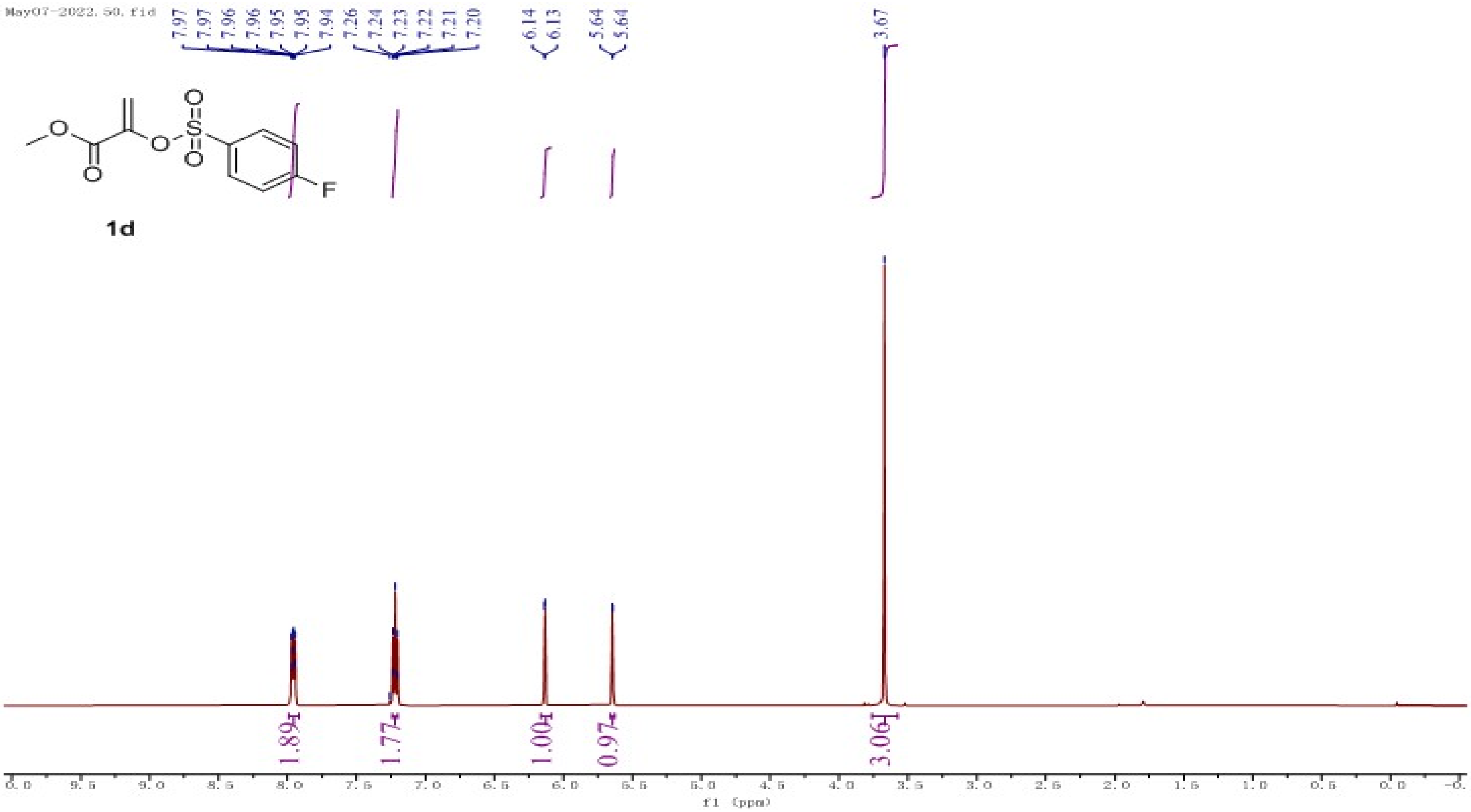
May07-2022.50.fid
7.97
7.97
7.96
7.96
7.95
7.95
7.94
7.26
7.24
7.23
7.22
7.21
7.20
6.14
6.13
5.64
5.64
3.67
1d
1.89
1.77
1.00
0.97
3.06
f1 (ppm)

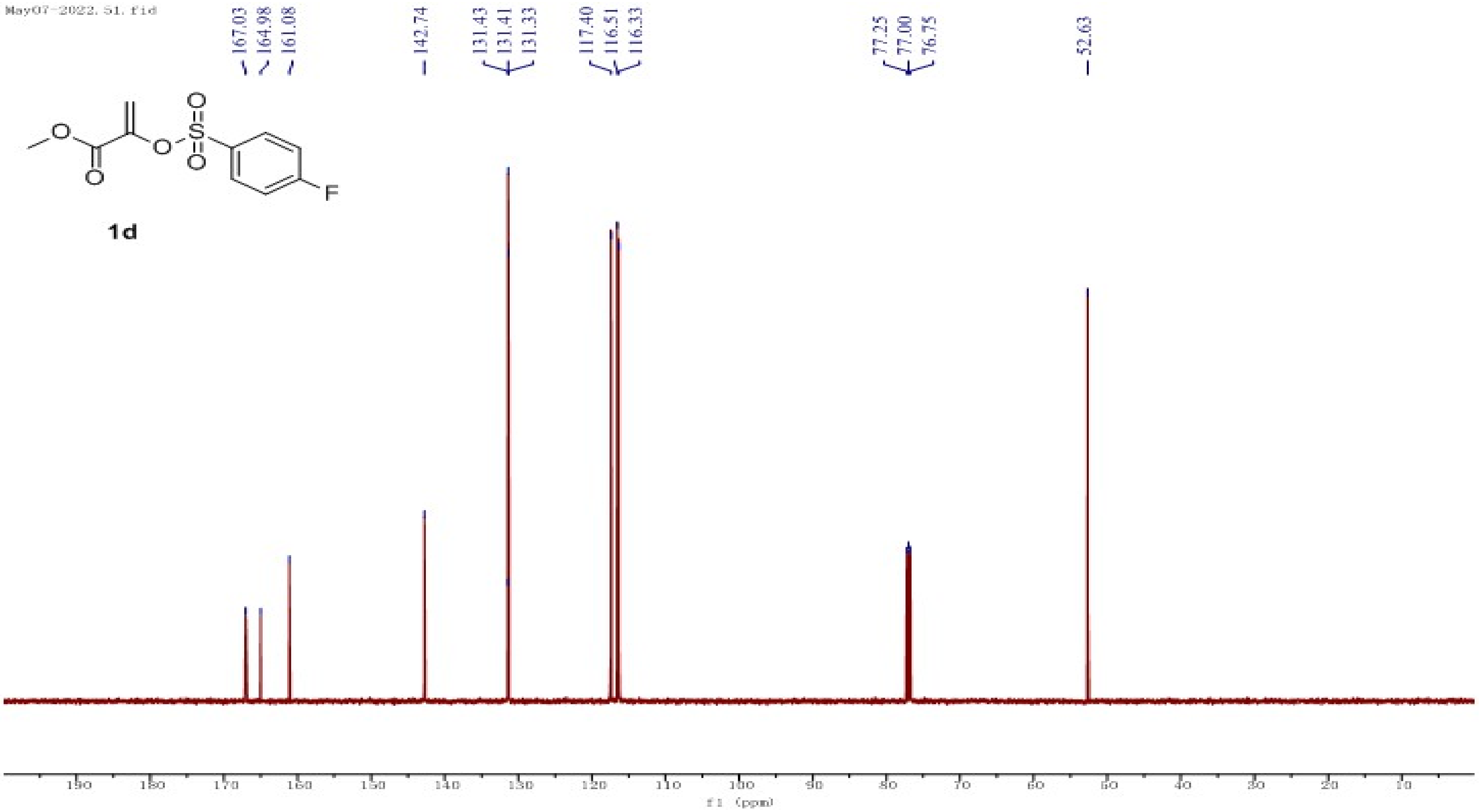
May07-2022.51.fid
167.03
164.98
161.08
142.74
131.43
131.41
131.33
117.40
116.51
116.33
77.25
77.00
76.75
52.63
F
1d
190
180
170
160
150
140
130
120
110
100
90
80
70
60
50
40
30
20
10
f1 (ppm)

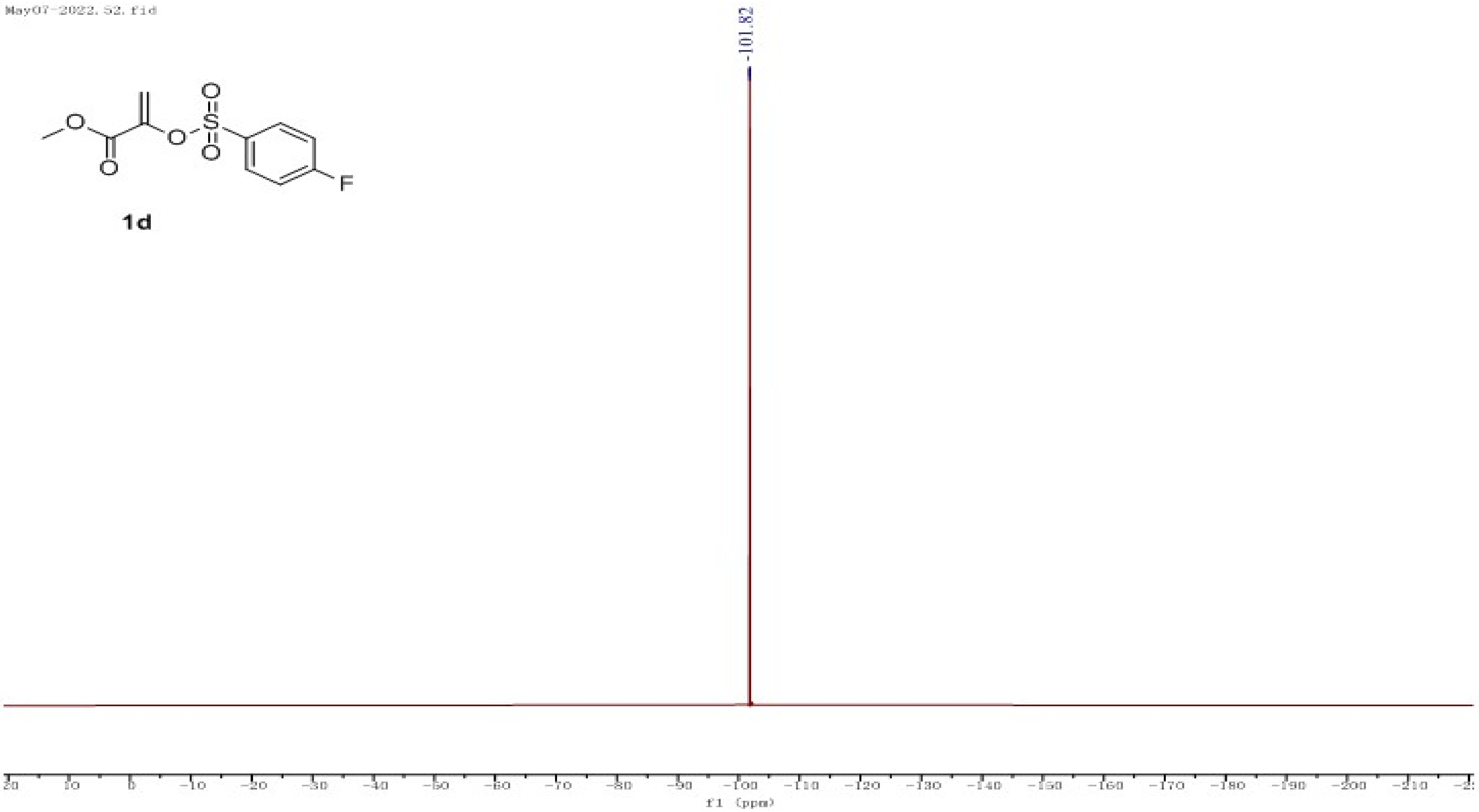
May07-2022.52.fid
-101.82
F
20 10 0 -10 -20 -30 -40 -50 -60 -70 -80 -90 -100 -110 -120 -130 -140 -150 -160 -170 -180 -190 -200 -210
f1 (ppm)

1d

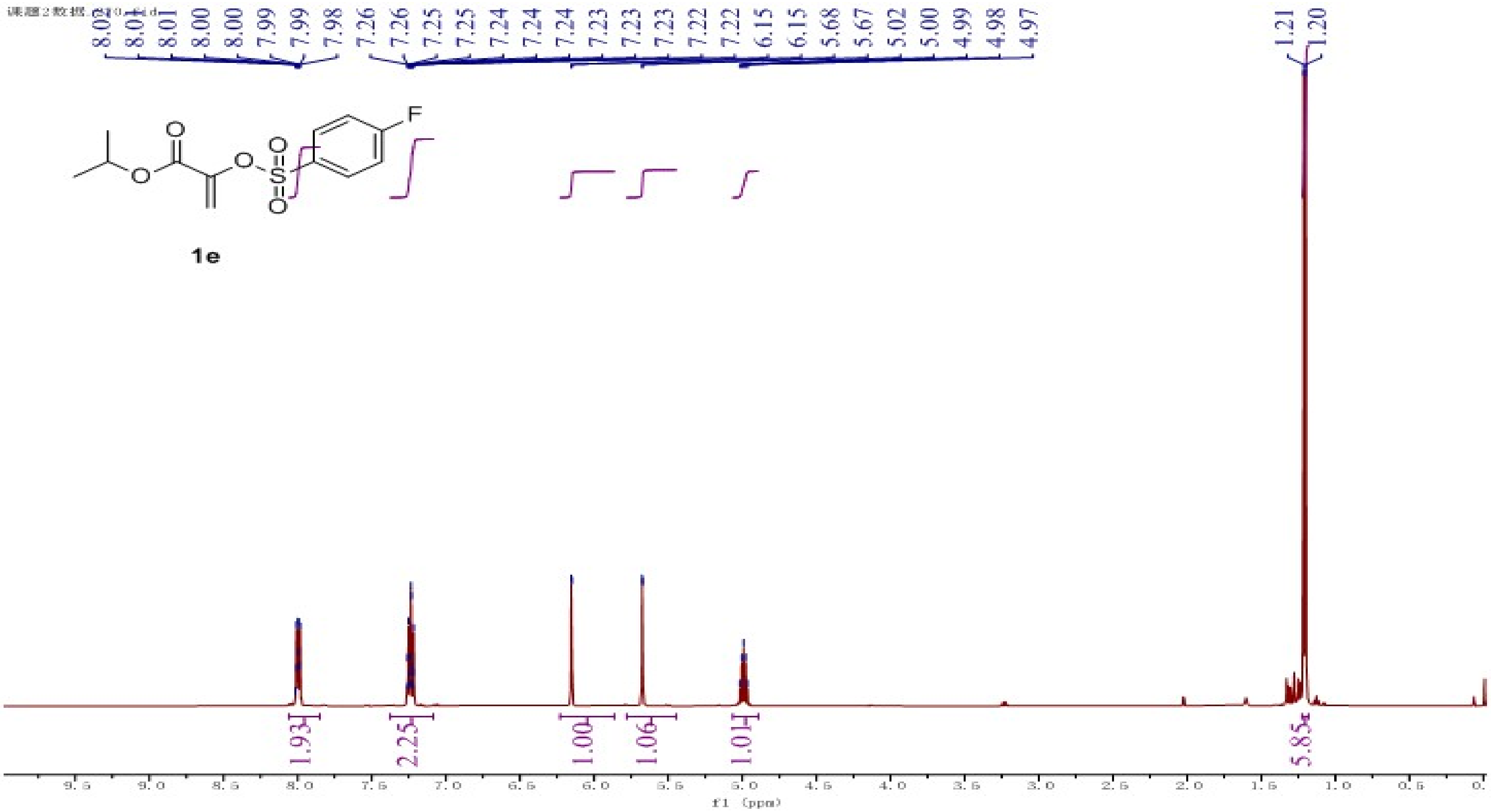
8.02 8.01 8.01 8.00 8.00 7.99 7.99 7.98 7.26 7.26 7.25 7.25 7.24 7.24 7.24 7.23 7.23 7.23 7.22 7.22 6.15 6.15 5.68 5.67 5.02 5.00 4.99 4.98 4.97
1.21 1.20
F
O
S
1e
1.93
2.25
1.00
1.06
1.01
5.85
9.5 9.0 8.5 8.0 7.5 7.0 6.5 6.0 5.5 5.0 4.5 4.0 3.5 3.0 2.5 2.0 1.5 1.0 0.5
f1 (ppm)

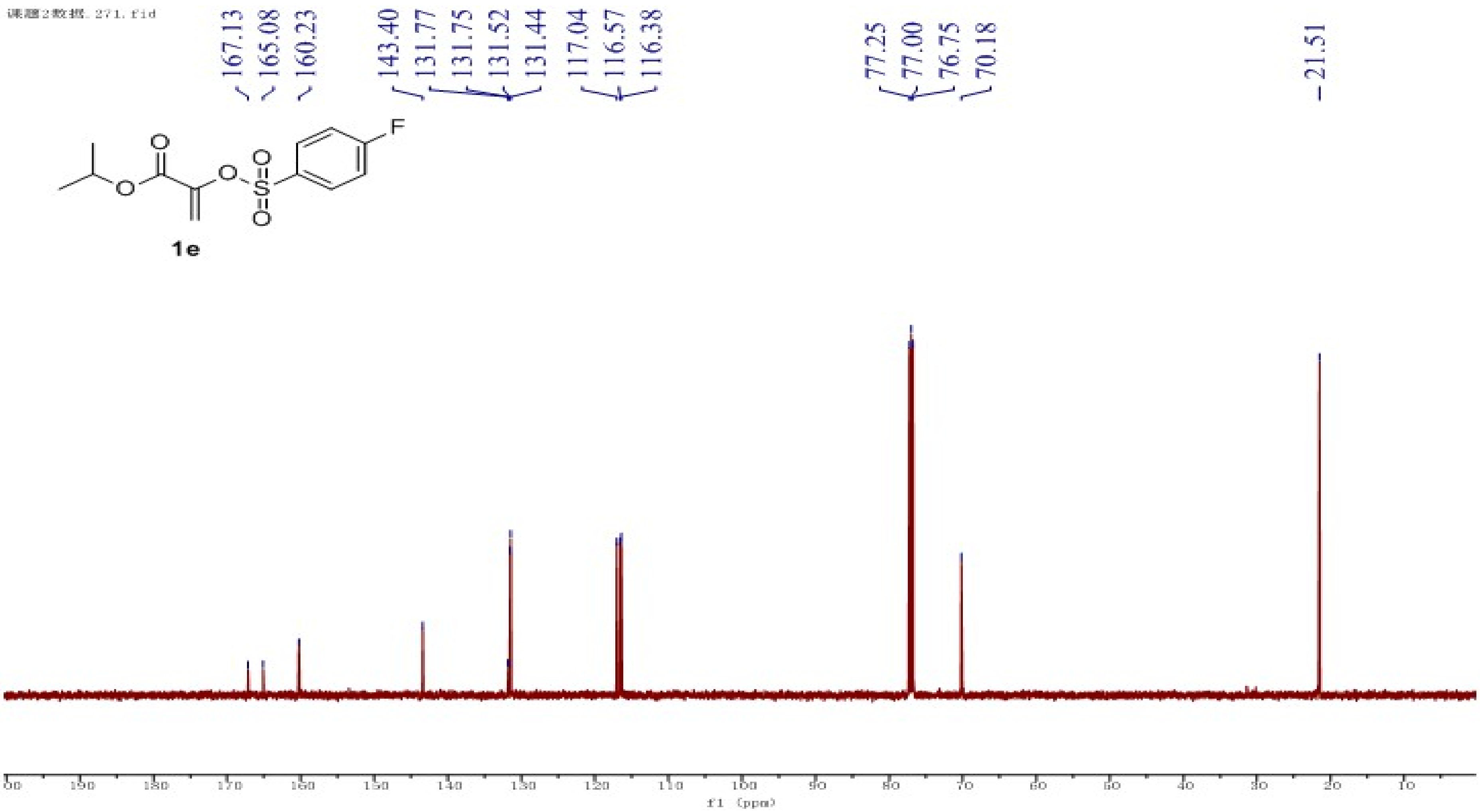

课题2数据.271.fid
167.13
165.08
160.23
143.40
131.77
131.75
131.52
131.44
117.04
116.57
116.38
77.25
77.00
76.75
70.18
21.51
F
O
O
O
S
O
O
1e
f1 (ppm)

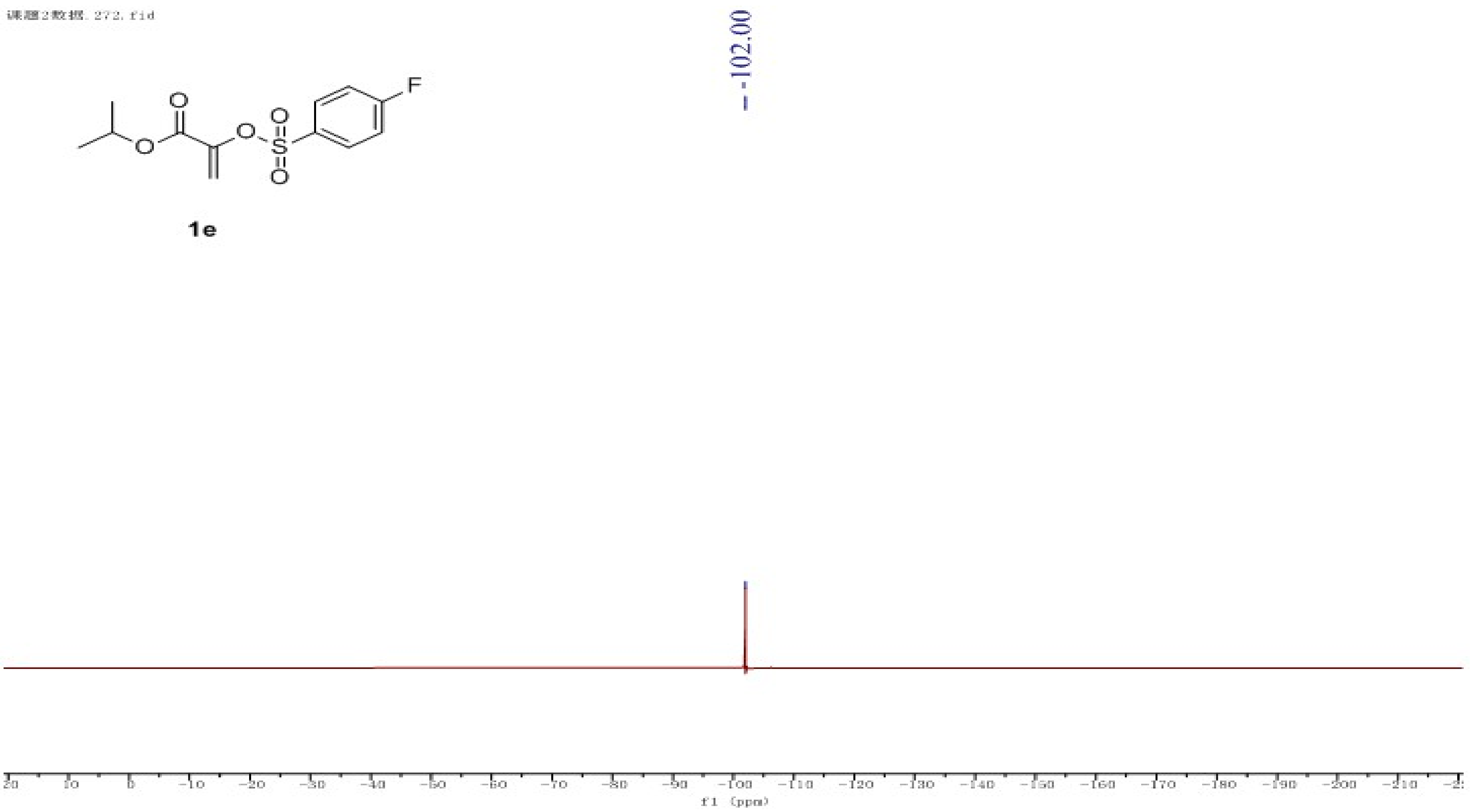
课题2数据.272.fid
-102.00
1e
20 10 0 -10 -20 -30 -40 -50 -60 -70 -80 -90 -100 -110 -120 -130 -140 -150 -160 -170 -180 -190 -200 -210
f1 (ppm)

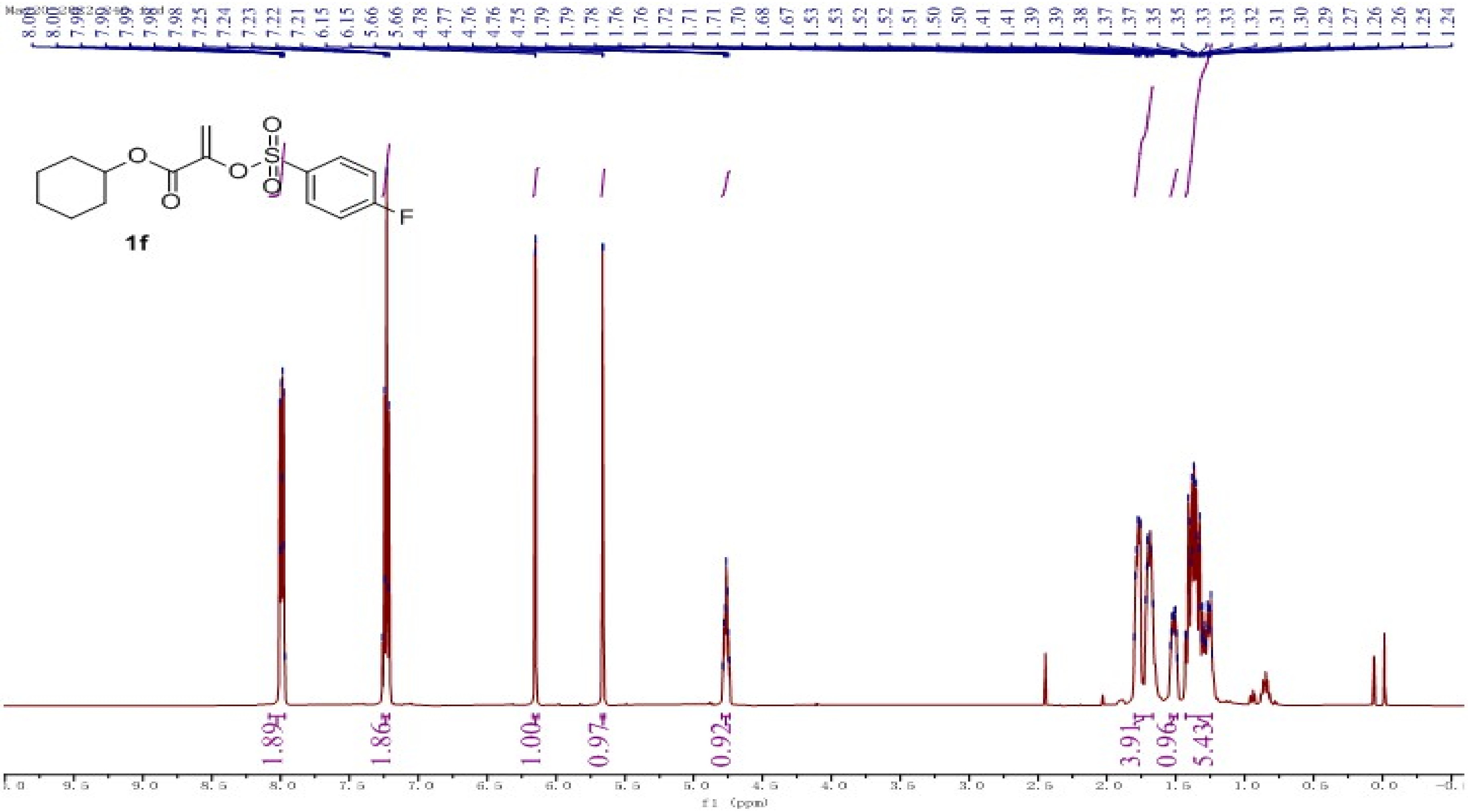

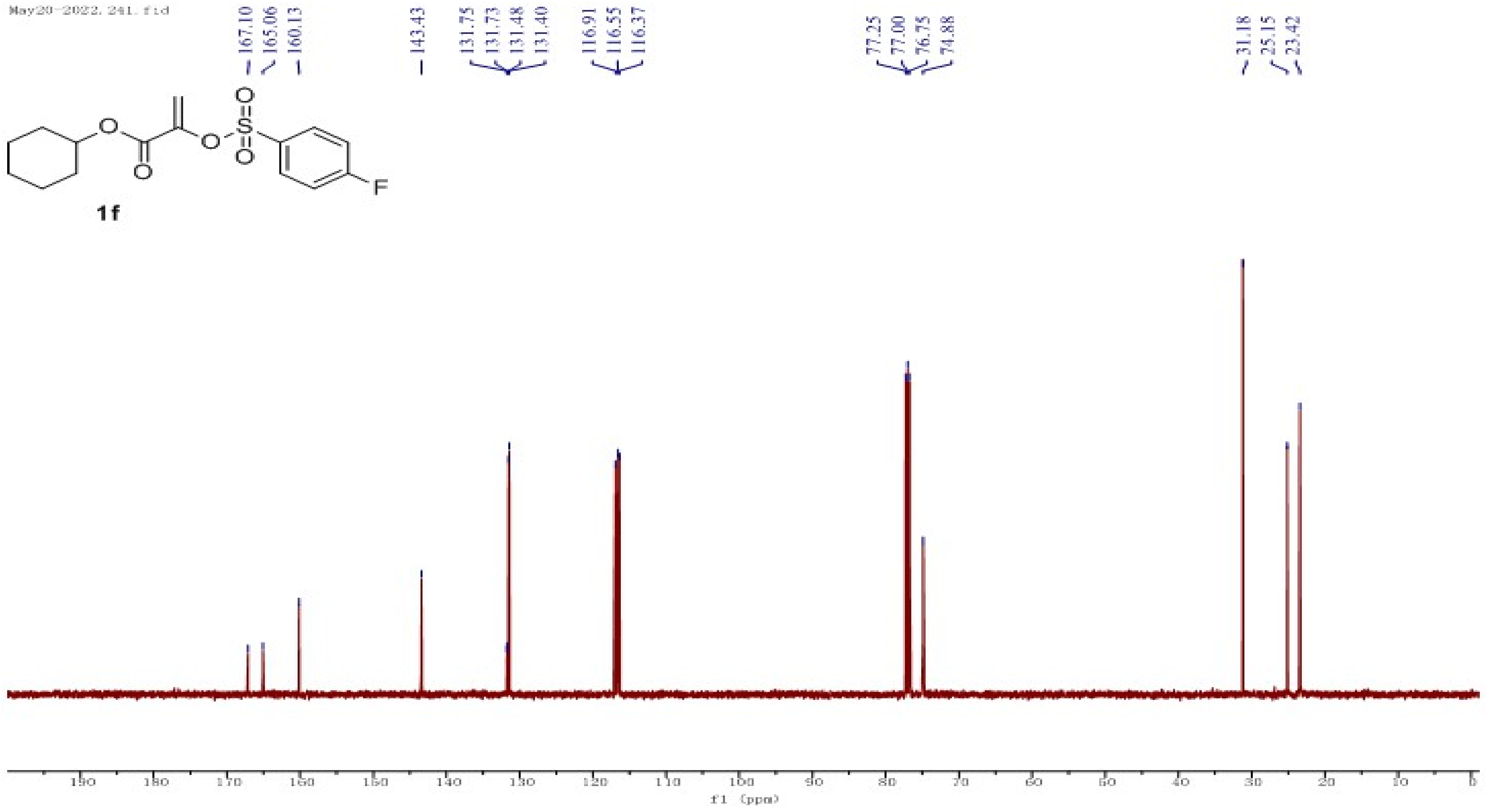
May20-2022.241.fid
167.10
165.06
160.13
143.43
131.75
131.73
131.48
131.40
116.91
116.55
116.37
77.25
77.00
76.75
74.88
31.18
25.15
23.42
O
O
O
S
O
O
F
1f
190
180
170
160
150
140
130
120
110
100
90
80
70
60
50
40
30
20
10
0
f1 (ppm)

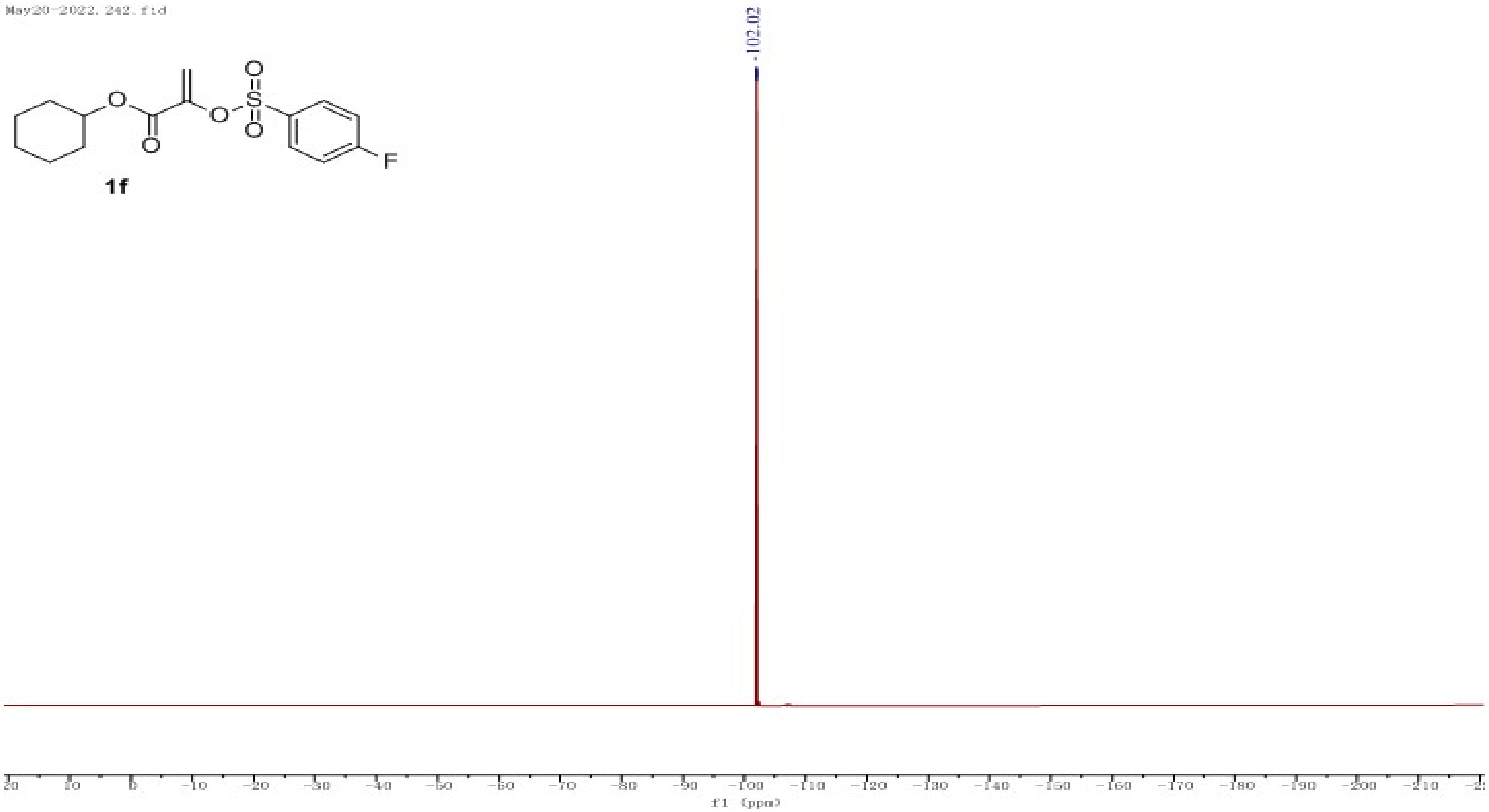
May20-2022.242.fid
-102.02
1f
f1 (ppm)

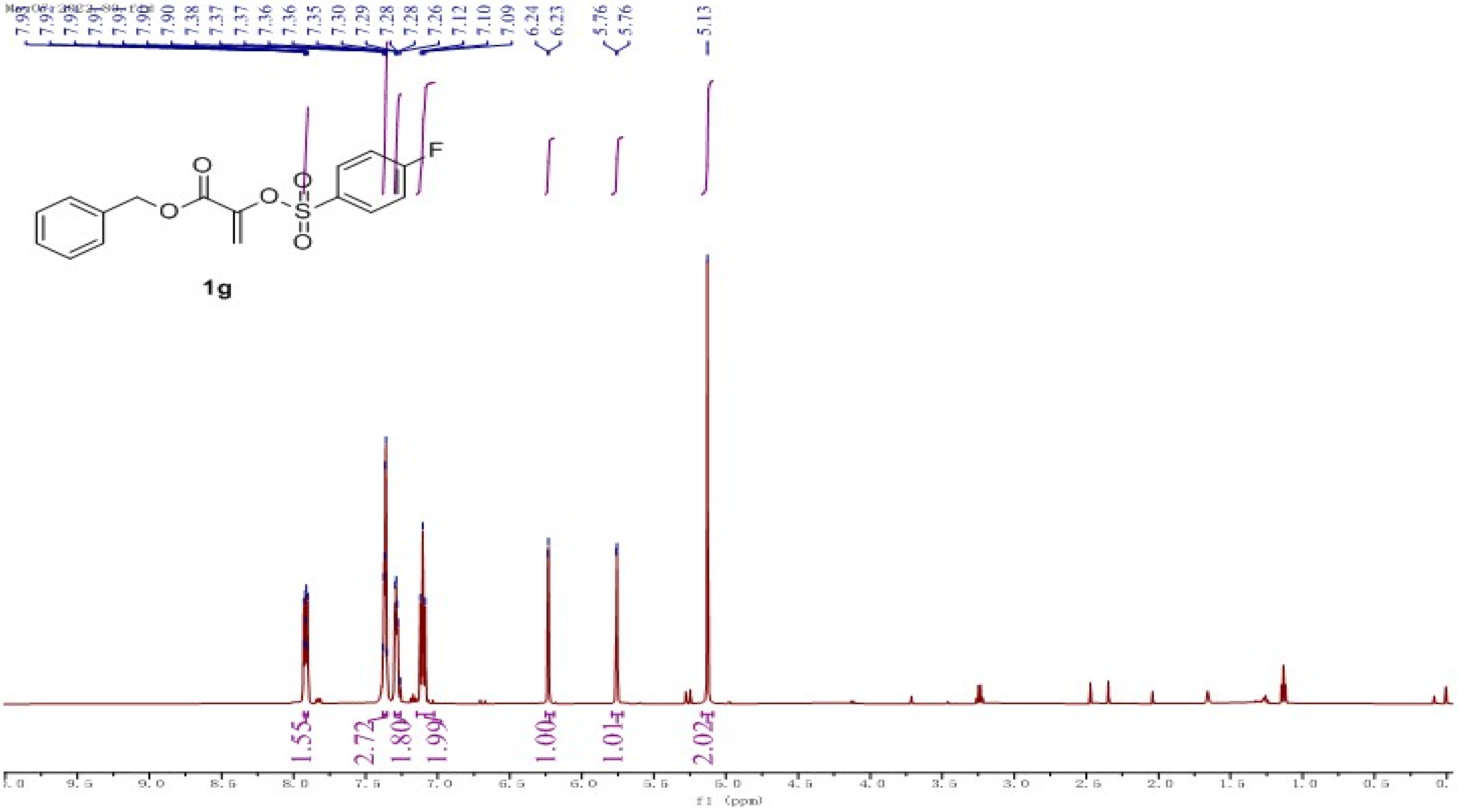

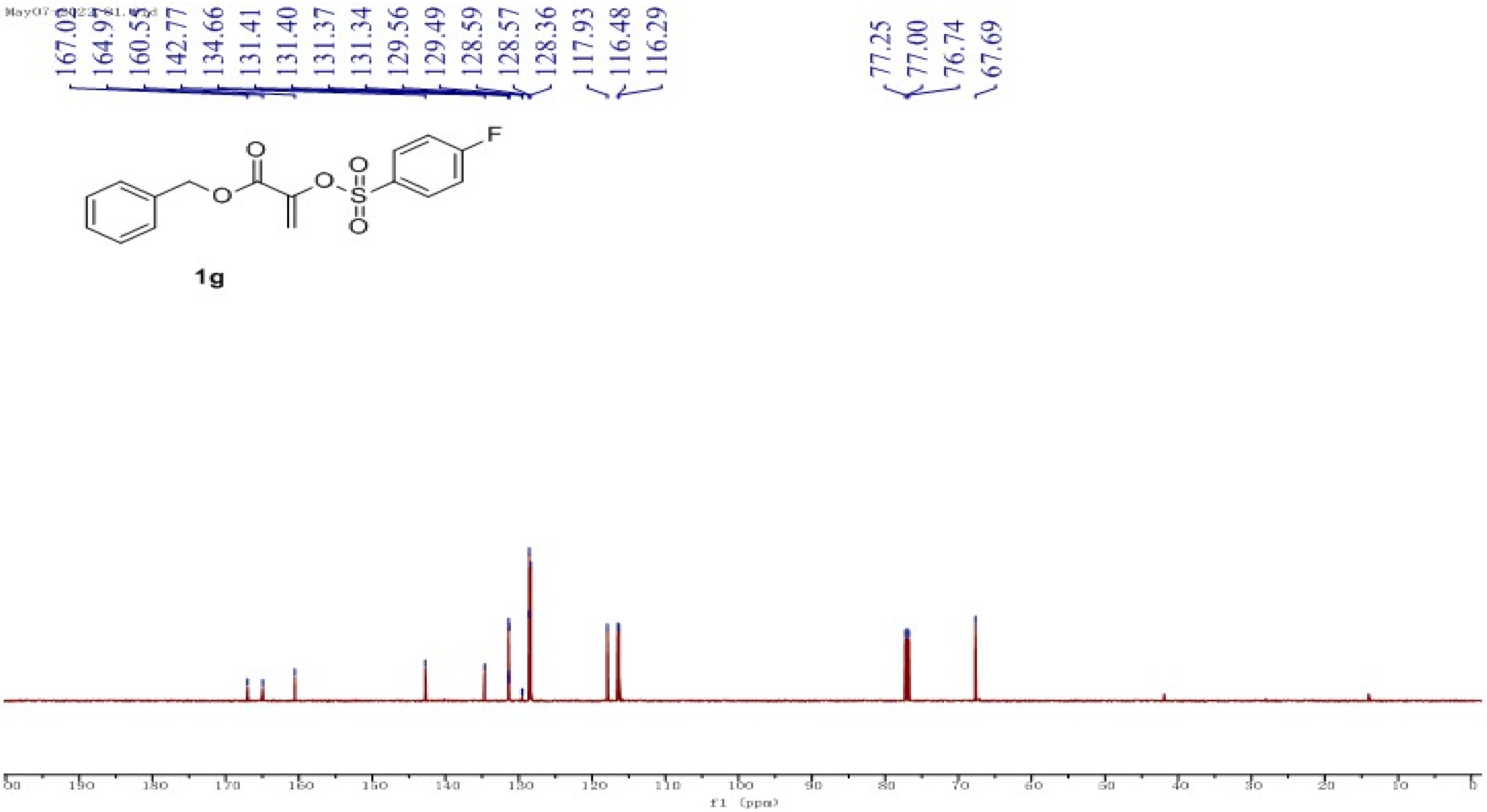
167.02
164.97
160.53
142.77
134.66
131.41
131.40
131.37
131.34
129.56
129.49
128.59
128.57
128.36
117.93
116.48
116.29
77.25
77.00
76.74
67.69
F
O
S
1g
190
180
170
160
150
140
130
120
110
100
90
80
70
60
50
40
30
20
10
0
f1 (ppm)

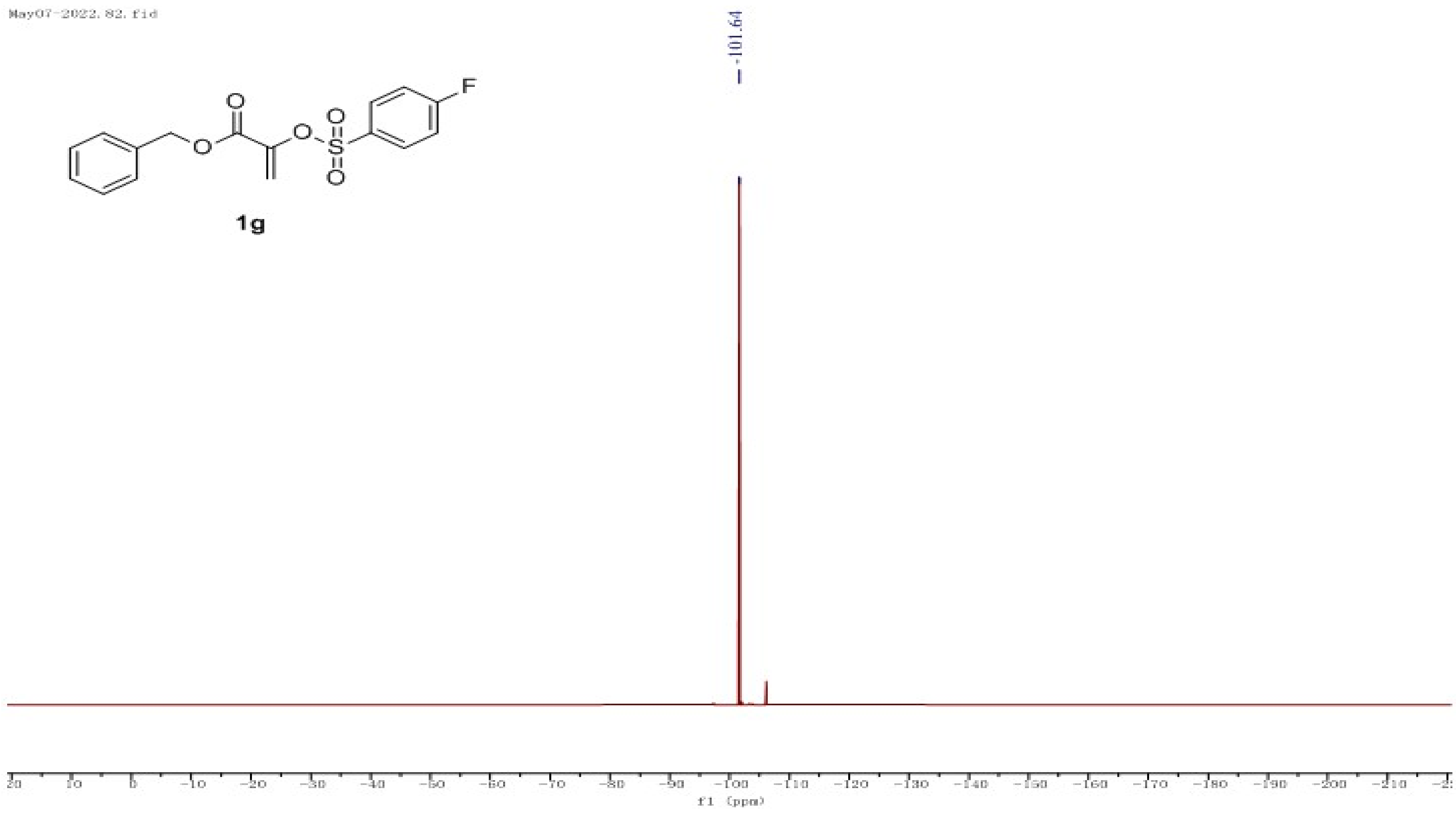
May07-2022.82.fid
-101.64
1g
20 10 0 -10 -20 -30 -40 -50 -60 -70 -80 -90 -100 -110 -120 -130 -140 -150 -160 -170 -180 -190 -200 -210
f1 (ppm)

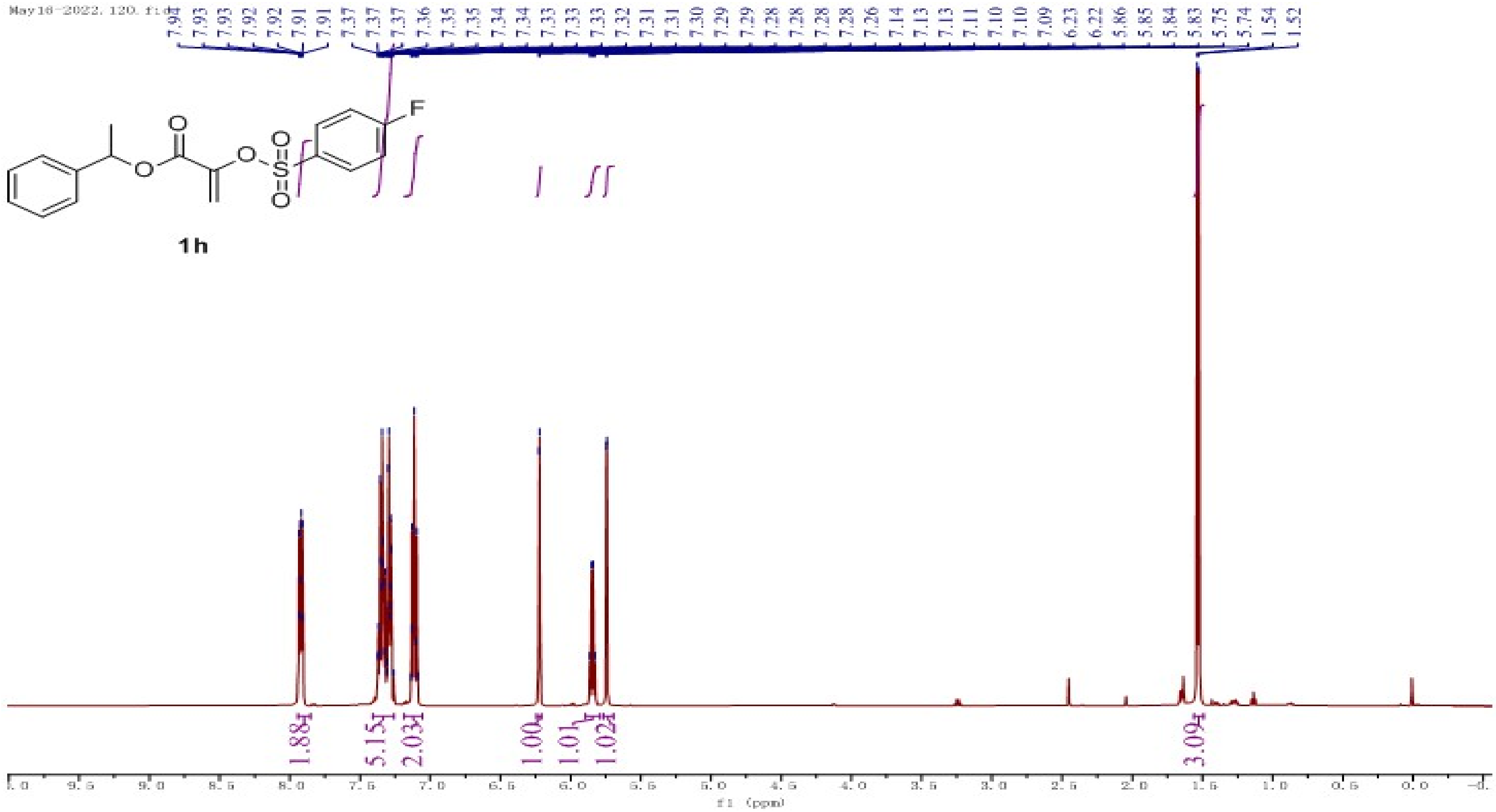
May16-2022.120.fid
7.94 7.93 7.93 7.92 7.92 7.91 7.91 7.37 7.37 7.37 7.36 7.35 7.35 7.34 7.34 7.33 7.33 7.33 7.32 7.31 7.31 7.30 7.29 7.29 7.28 7.28 7.28 7.28 7.26 7.14 7.13 7.13 7.11 7.10 7.10 7.09 6.23 6.22 5.86 5.85 5.84 5.83 5.75 5.74 1.54 1.52
F
O
O
O
S
O
O
1.88
5.15
2.03
1.00
1.01
1.02
3.09
f1 (ppm)

1h

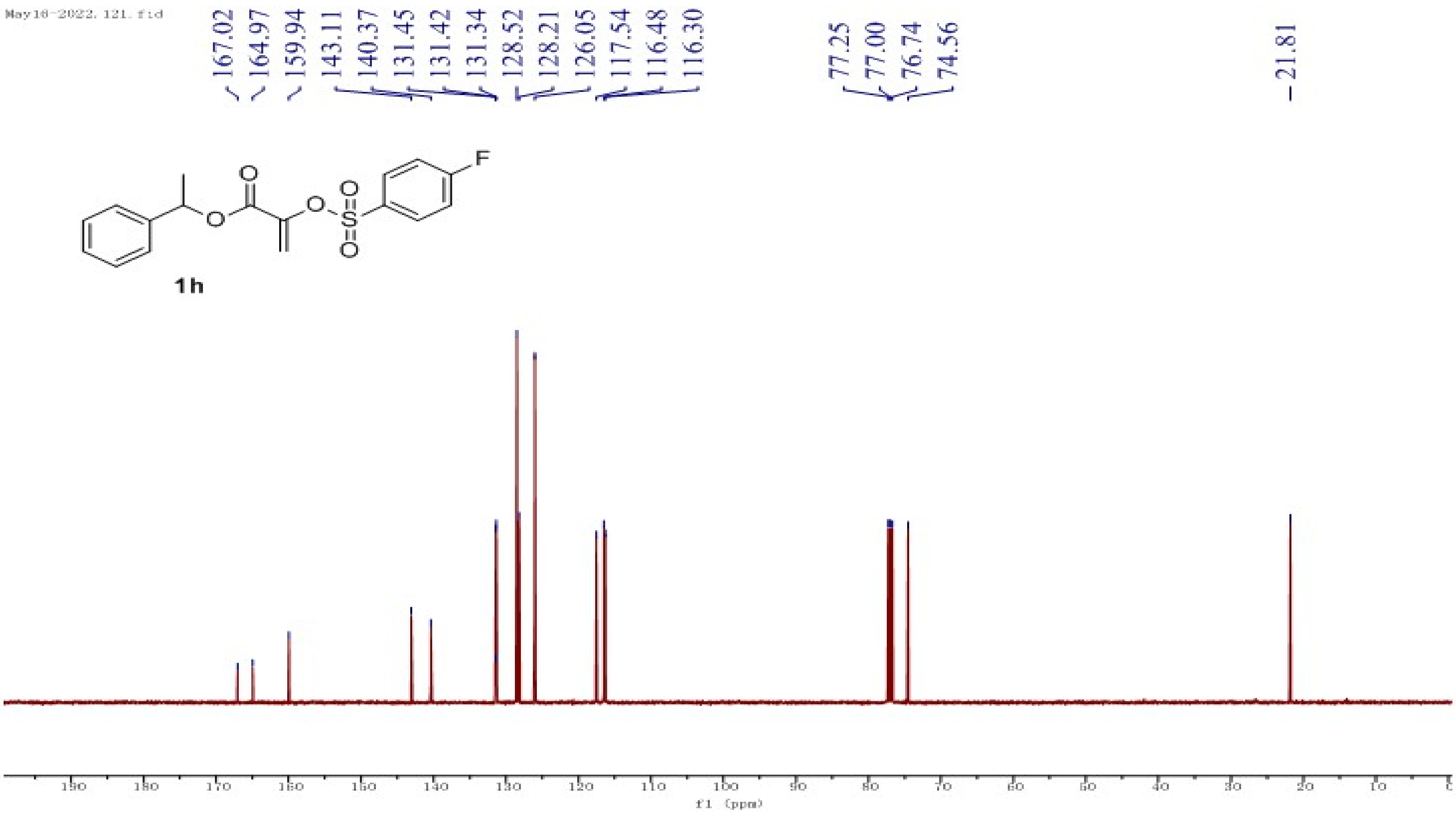
May16-2022.121.fid
167.02
164.97
159.94
143.11
140.37
131.45
131.42
131.34
128.52
128.21
126.05
117.54
116.48
116.30
77.25
77.00
76.74
74.56
21.81
1h
f1 (ppm)

May16-2022.122.fid

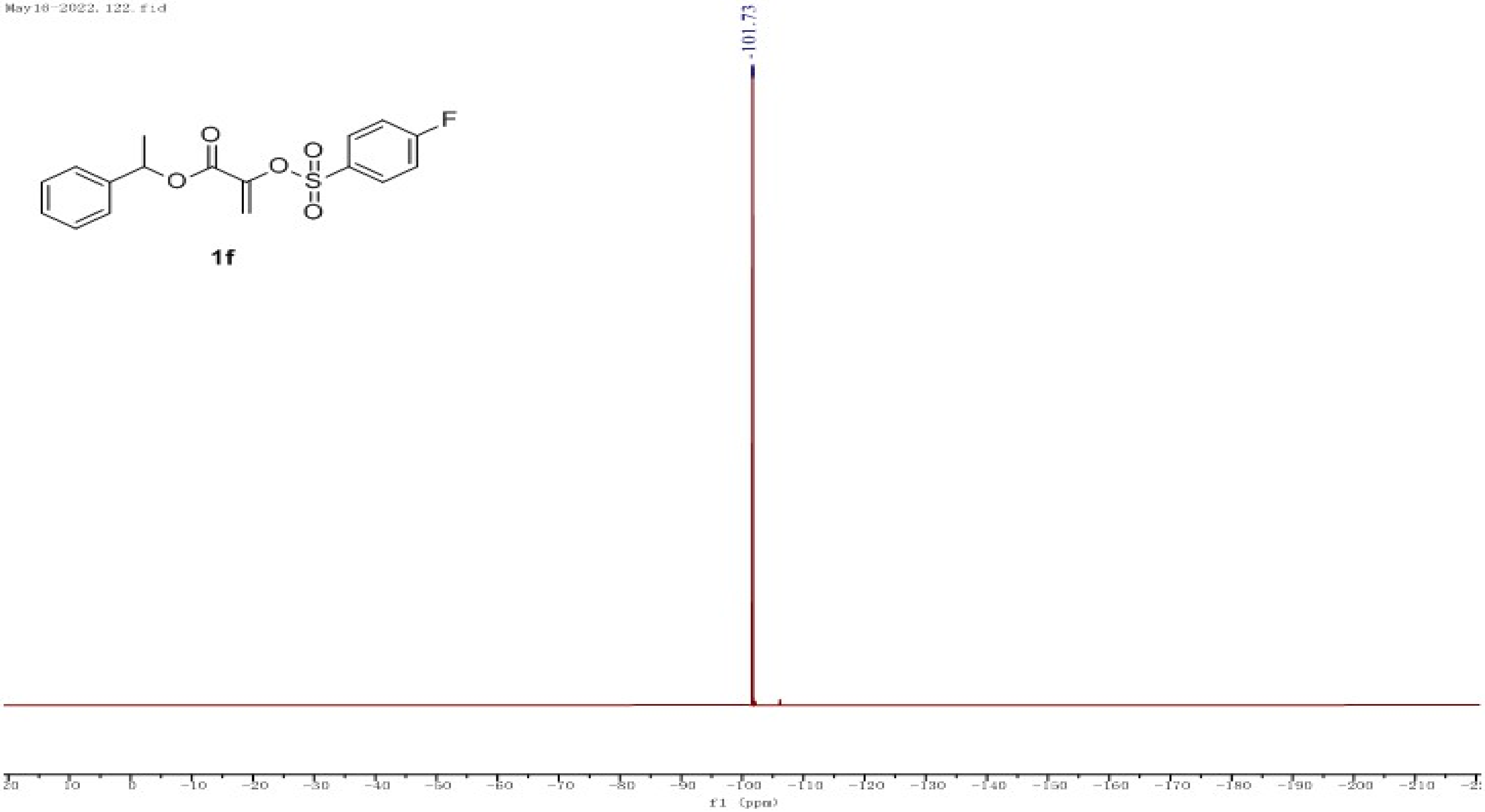

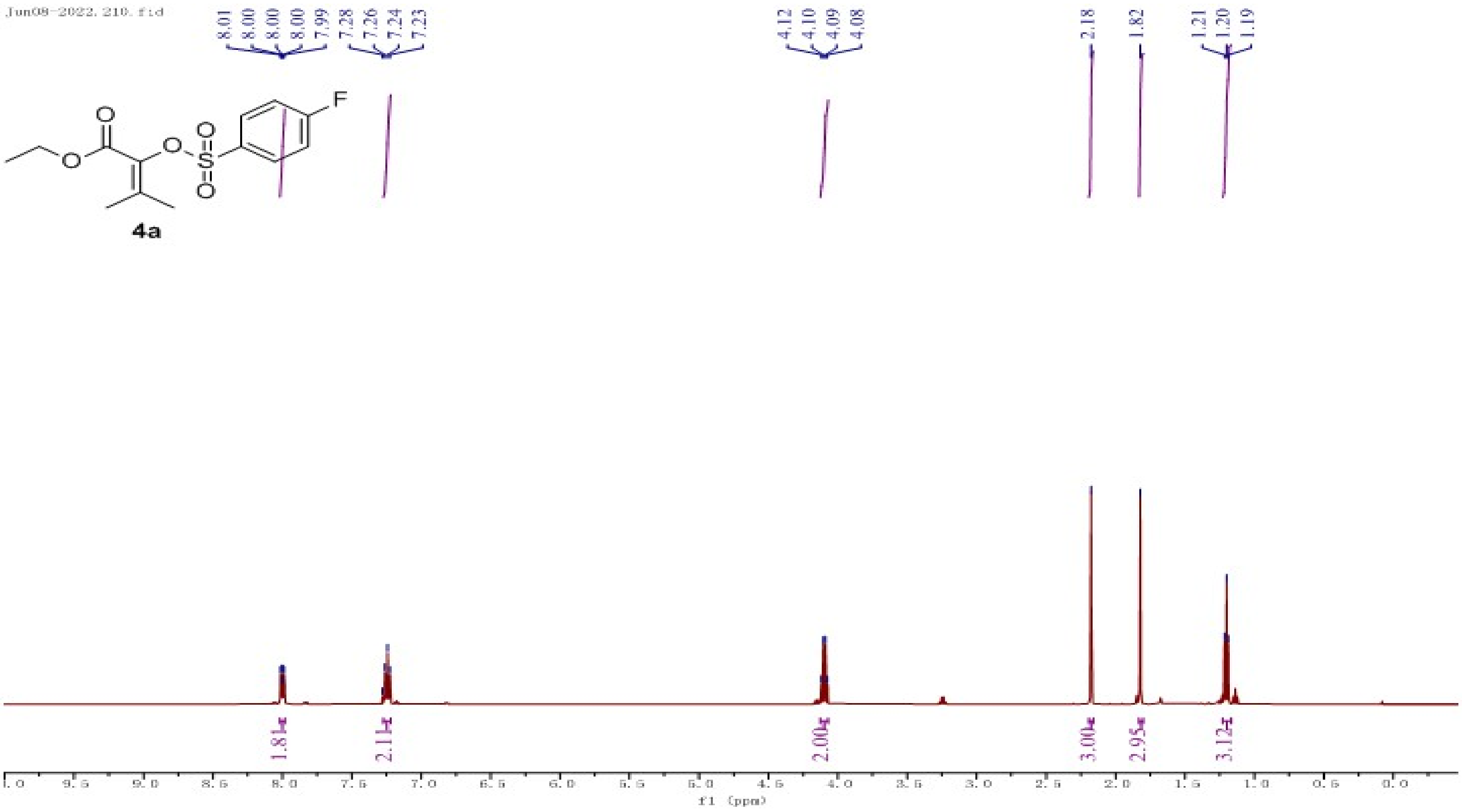
Jun08-2022.210.fid
8.01
8.00
8.00
8.00
7.99
7.28
7.26
7.24
7.23
4.12
4.10
4.09
4.08
2.18
1.82
1.21
1.20
1.19
F
4a
1.81
2.11
2.00
3.00
2.95
3.12
9.5
9.0
8.5
8.0
7.5
7.0
6.5
6.0
5.5
5.0
4.5
4.0
3.5
3.0
2.5
2.0
1.5
1.0
0.5
0.0
f1 (ppm)

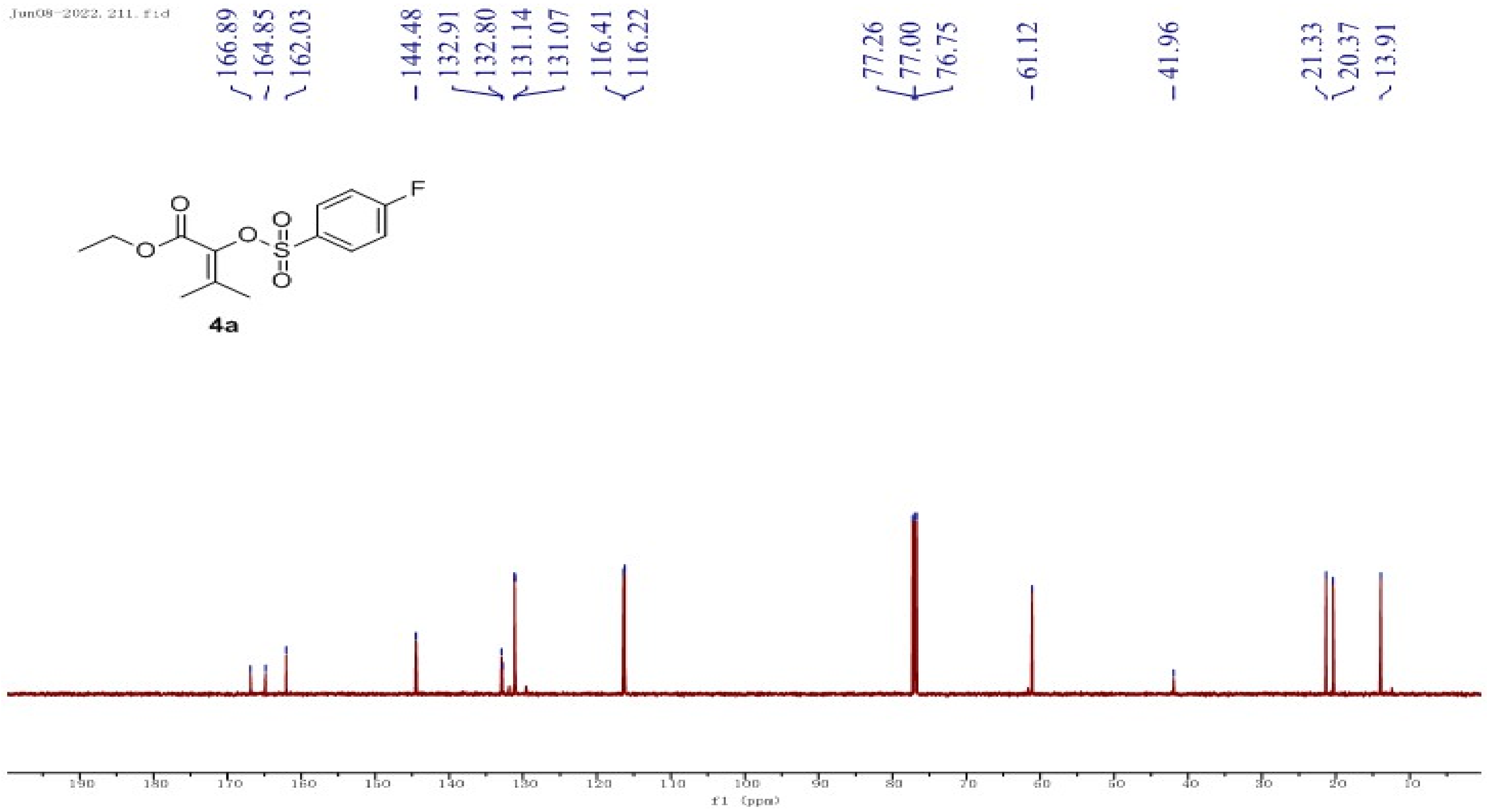

Jun08-2022.211.fid
166.89
164.85
162.03
144.48
132.91
132.80
131.14
131.07
116.41
116.22
77.26
77.00
76.75
61.12
41.96
21.33
20.37
13.91
F
O
S
4a
190
180
170
160
150
140
130
120
110
100
90
80
70
60
50
40
30
20
10
f1 (ppm)

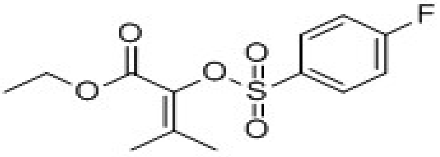

4a

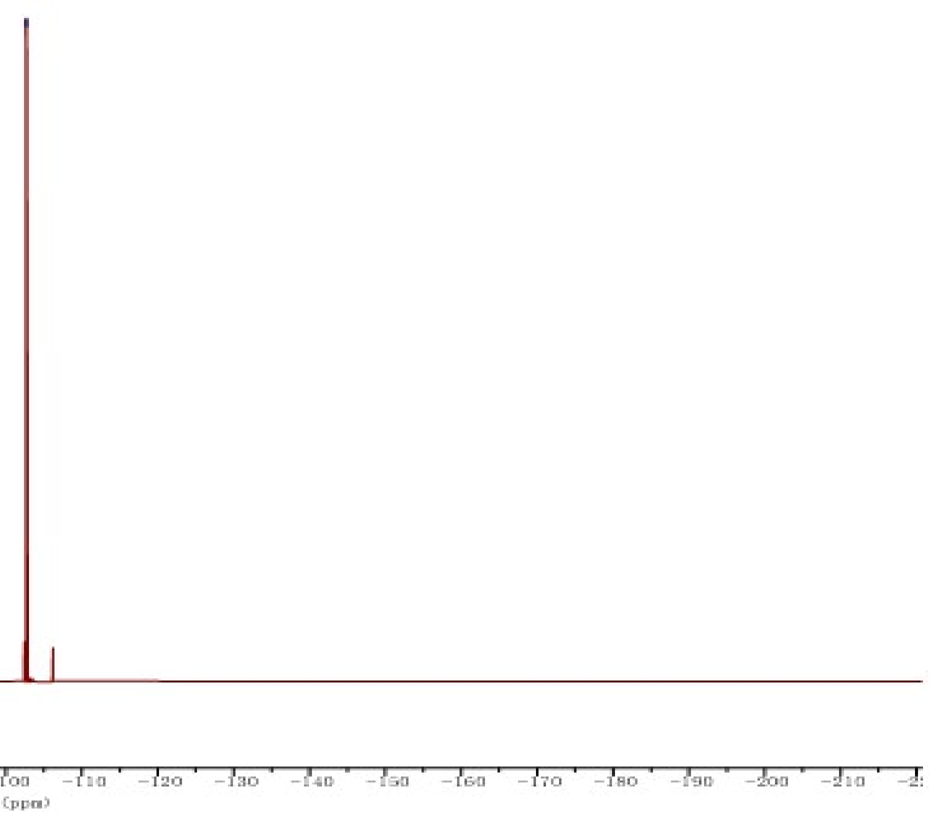
Jun08-2022.212.fid
-102.73
20
10
0
-10
-20
-30
-40
-50
-60
-70
-80
-90
-100
-110
-120
-130
-140
-150
-160
-170
-180
-190
-200
-210
f1 (ppm)

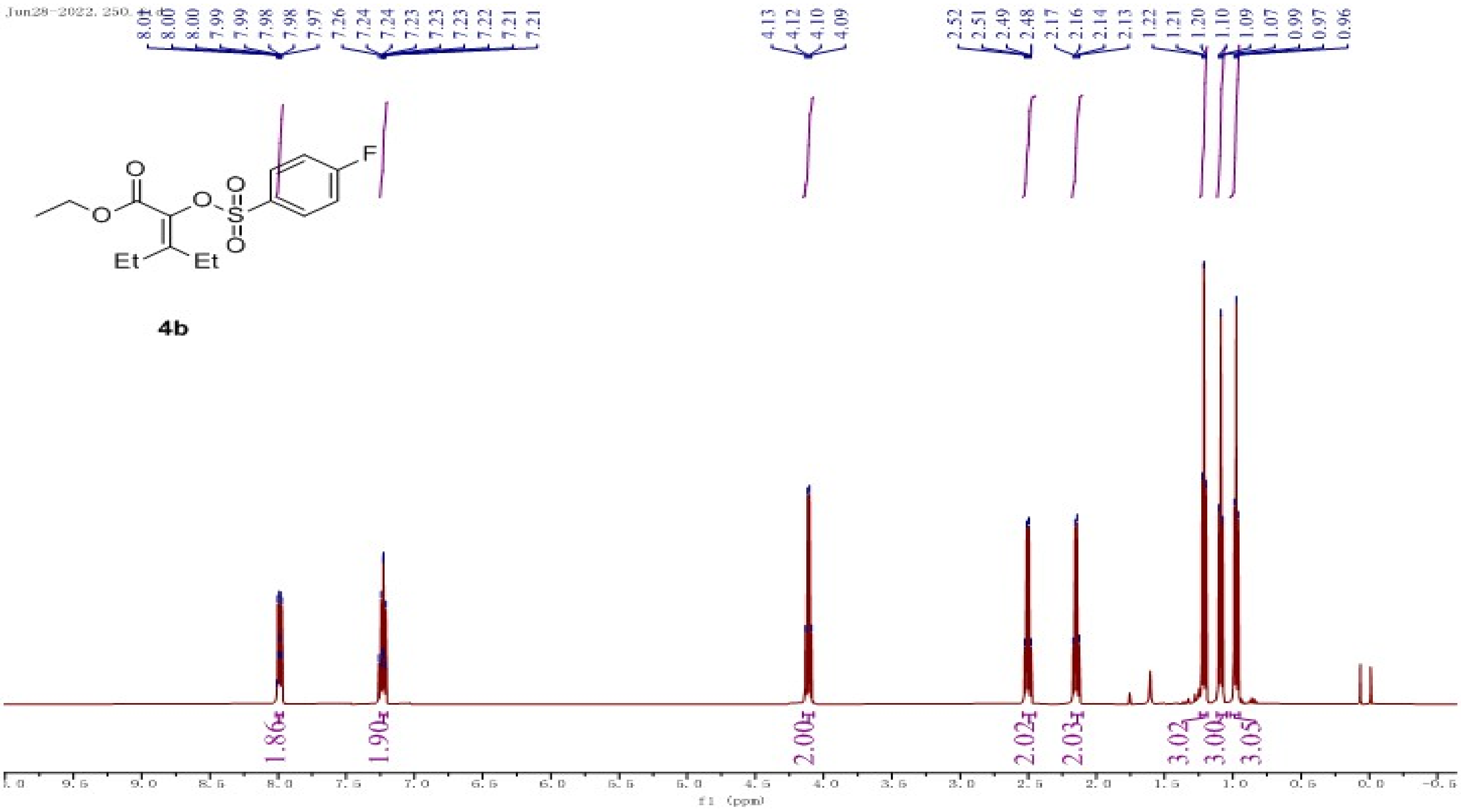
Jun28-2022.250.1.fid
8.01 8.00 8.00 7.99 7.99 7.98 7.98 7.97 7.26 7.24 7.24 7.23 7.23 7.23 7.22 7.21 7.21
4.13 4.12 4.10 4.09
2.52 2.51 2.49 2.48 2.17 2.16 2.14 2.13 1.22 1.21 1.20 1.10 1.09 1.07 0.99 0.97 0.96
F
O
O
O
S
O
O
Et
Et
1.86
1.90
2.00
2.02
2.03
3.02
3.00
3.05
f1 (ppm)

4b

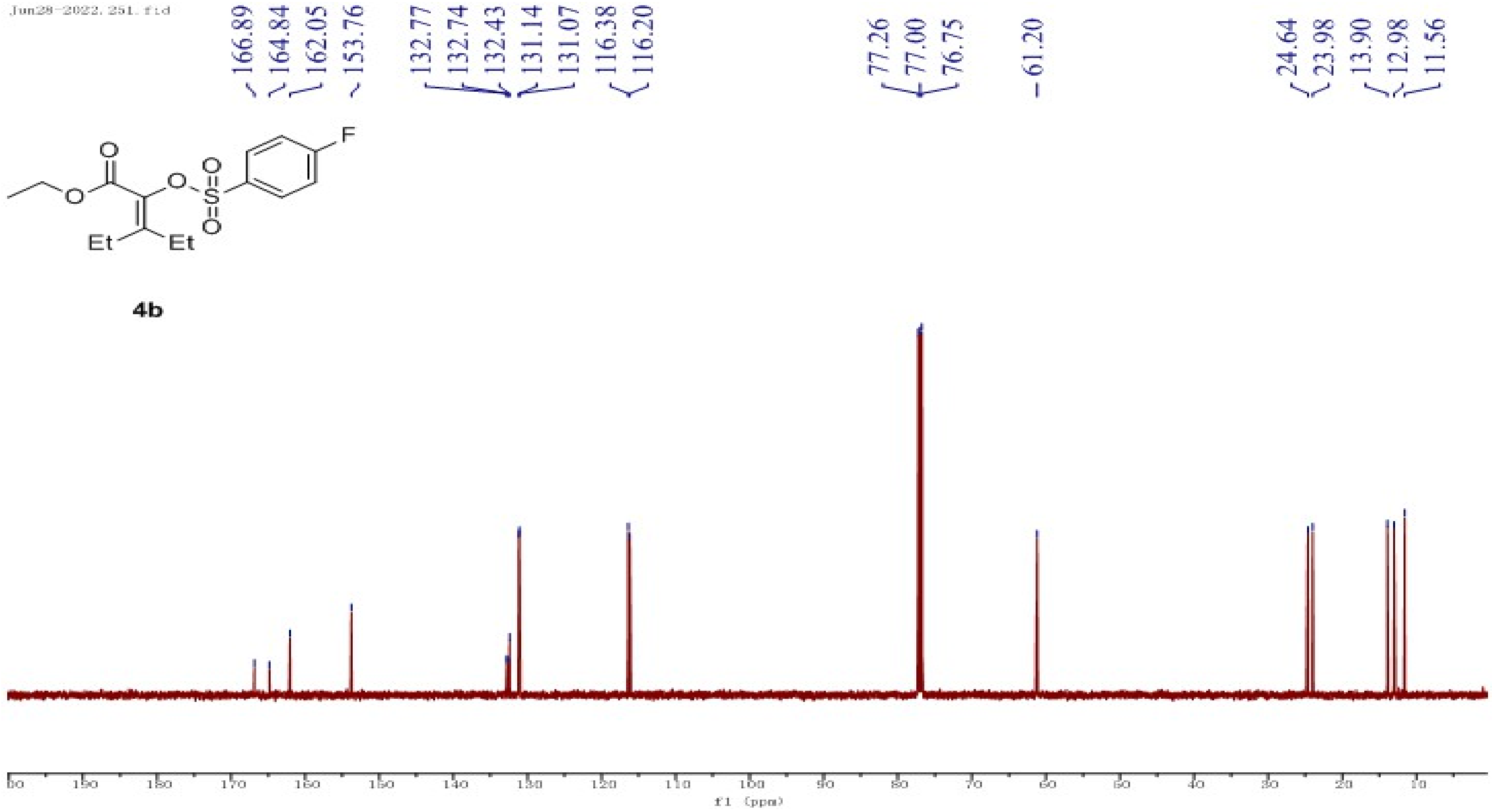
Jun28-2022.251.fid
166.89
164.84
162.05
153.76
132.77
132.74
132.43
131.14
131.07
116.38
116.20
77.26
77.00
76.75
61.20
24.64
23.98
13.90
12.98
11.56
F
O
O
O
S
O
O
Et
Et
4b
f1 (ppm)

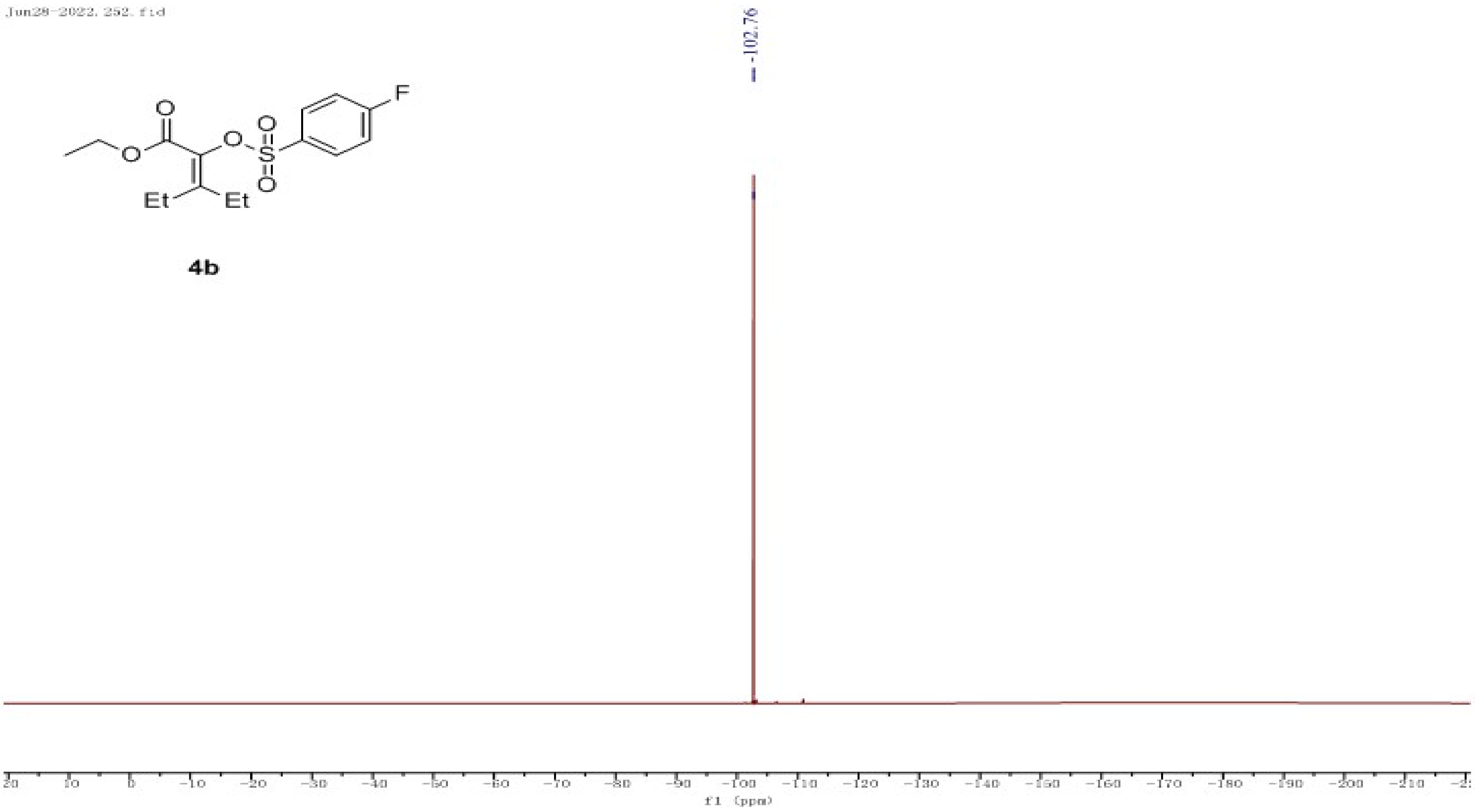
Jun28-2022.252.fid
-102.76
F
O
O
O
S
O
O
Et
Et
4b
20
10
0
-10
-20
-30
-40
-50
-60
-70
-80
-90
-100
-110
-120
-130
-140
-150
-160
-170
-180
-190
-200
-210
f1 (ppm)

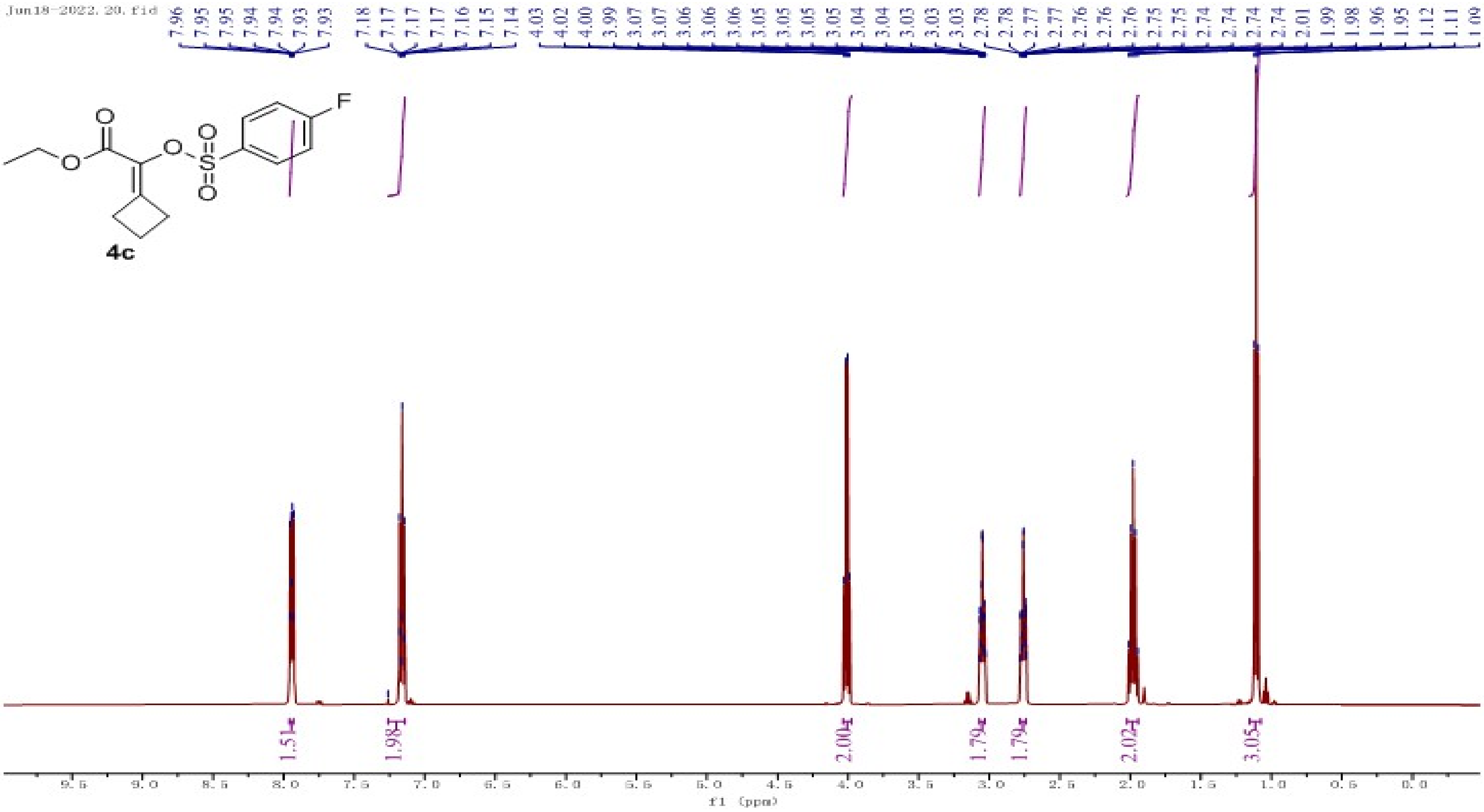
Jun18-2022.20.fid
7.96
7.95
7.95
7.94
7.94
7.93
7.93
7.18
7.17
7.17
7.17
7.16
7.15
7.14
4.03
4.02
4.00
3.99
3.07
3.07
3.06
3.06
3.06
3.05
3.05
3.05
3.05
3.04
3.04
3.03
3.03
3.03
2.78
2.78
2.77
2.77
2.76
2.76
2.76
2.75
2.75
2.74
2.74
2.74
2.74
2.01
1.99
1.98
1.96
1.95
1.12
1.11
1.09
F
4c
1.51
1.98
2.00
1.79
1.79
2.02
3.05
9.5
9.0
8.5
8.0
7.5
7.0
6.5
6.0
5.5
5.0
4.5
4.0
3.5
3.0
2.5
2.0
1.5
1.0
0.5
0.0
f1 (ppm)

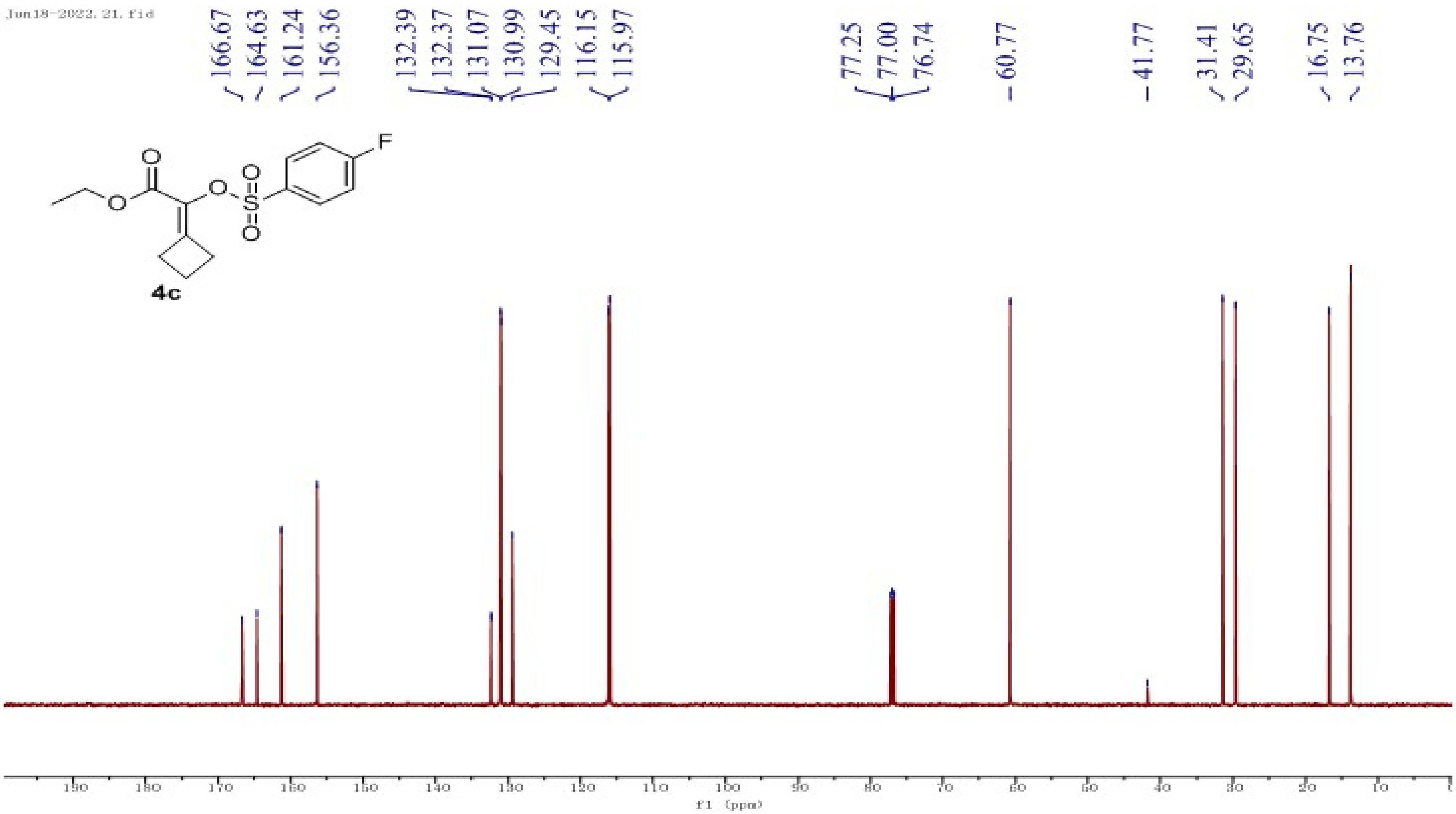

Jun18-2022.21.fid
166.67
164.63
161.24
156.36
132.39
132.37
131.07
130.99
129.45
116.15
115.97
77.25
77.00
76.74
60.77
41.77
31.41
29.65
16.75
13.76
F
O
S
4c
f1 (ppm)

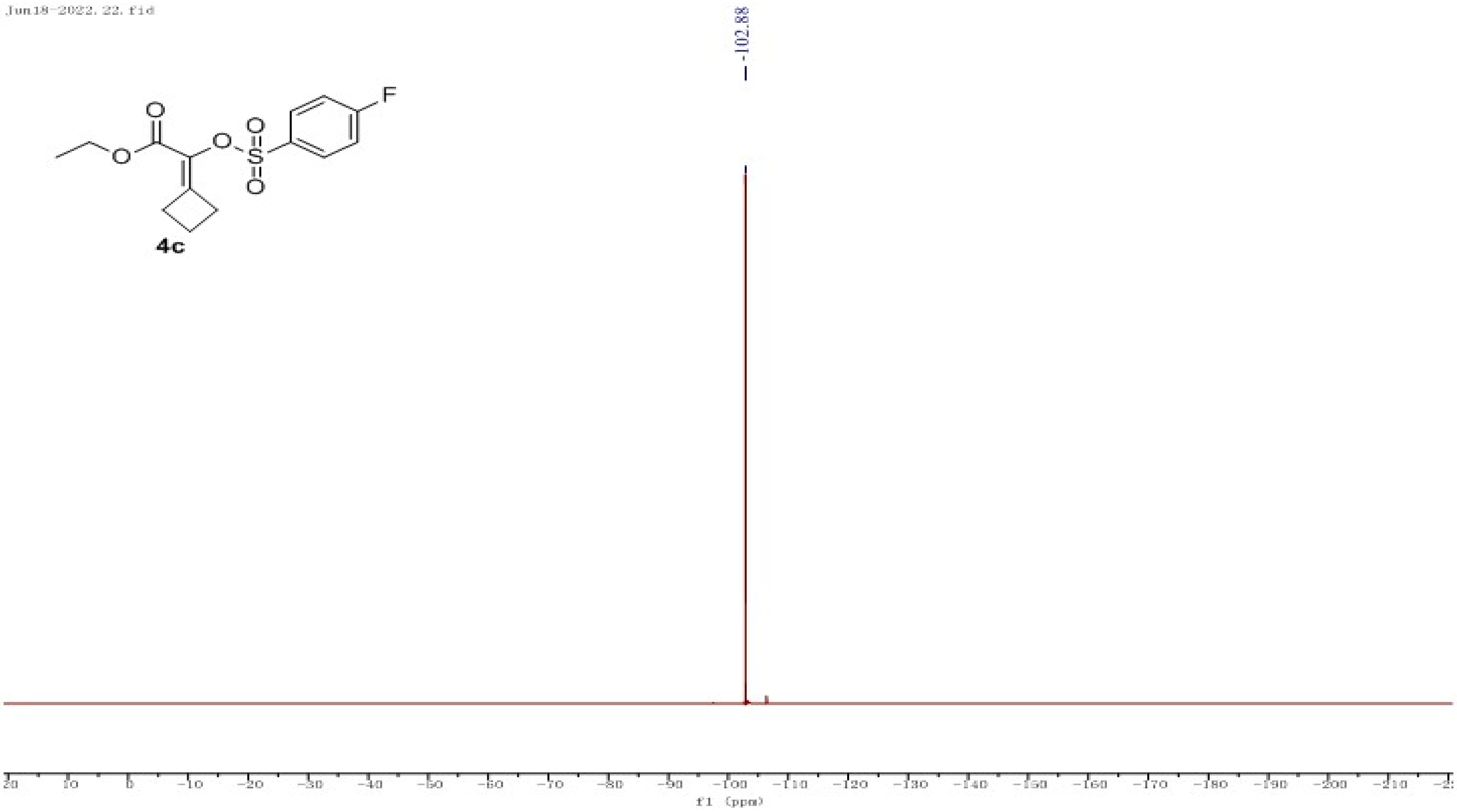
Jun18-2022.22.fid
-102.88
F
O
S
4c
20 10 0 -10 -20 -30 -40 -50 -60 -70 -80 -90 -100 -110 -120 -130 -140 -150 -160 -170 -180 -190 -200 -210
f1 (ppm)

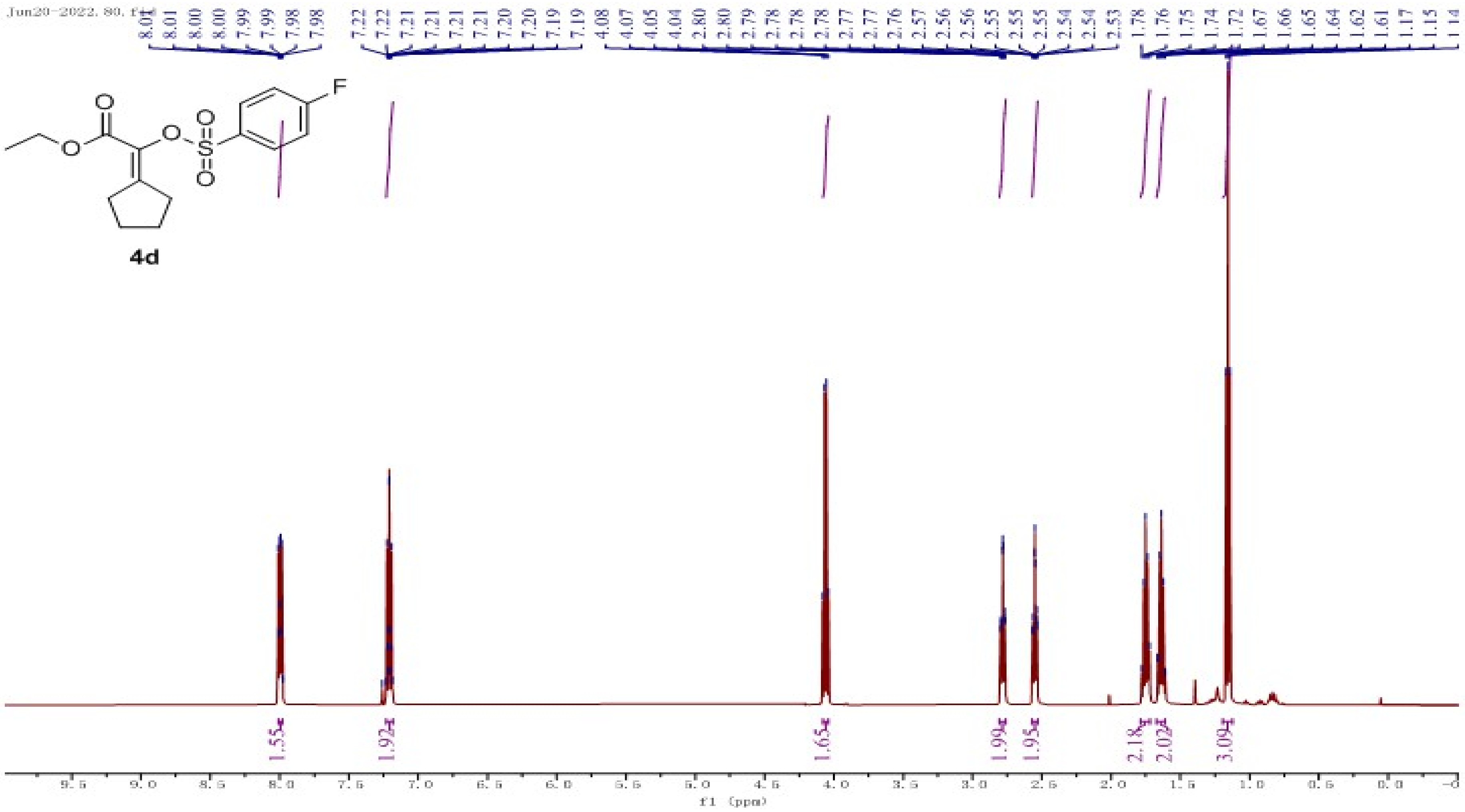
Jun20-2022.80.fid
8.01 8.01 8.00 8.00 7.99 7.99 7.98 7.98
7.22 7.22 7.21 7.21 7.21 7.21 7.20 7.20 7.19 7.19
4.08 4.07 4.05 4.04
2.80 2.80 2.79 2.78 2.78 2.78 2.77 2.77 2.76
2.57 2.56 2.56 2.55 2.55 2.55 2.54 2.54 2.53
1.78 1.76 1.75 1.74 1.72 1.67 1.66 1.65 1.64 1.62 1.61
1.17 1.15 1.14
4d
1.55
1.92
1.65
1.99
1.95
2.18
2.02
3.09
f1 (ppm)

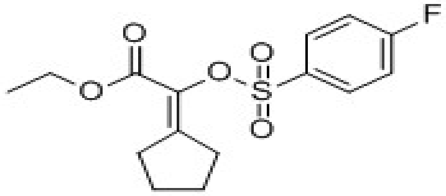

4d

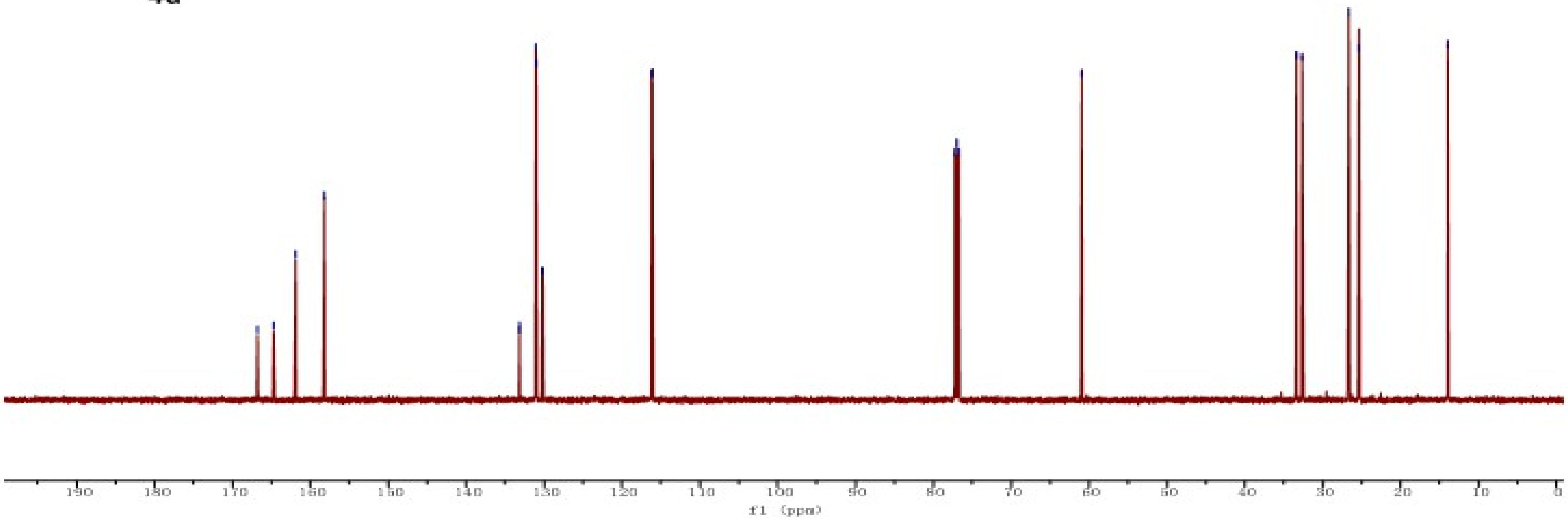

Jun20-2022.81.fid
166.76
164.72
161.83
158.21
133.16
133.13
131.06
130.98
130.18
116.25
116.07
77.26
77.00
76.75
60.97
33.36
32.65
26.64
25.35
13.94
190
180
170
160
150
140
130
120
110
100
90
80
70
60
50
40
30
20
10
0
f1 (ppm)

Jun20-2022.82.fid

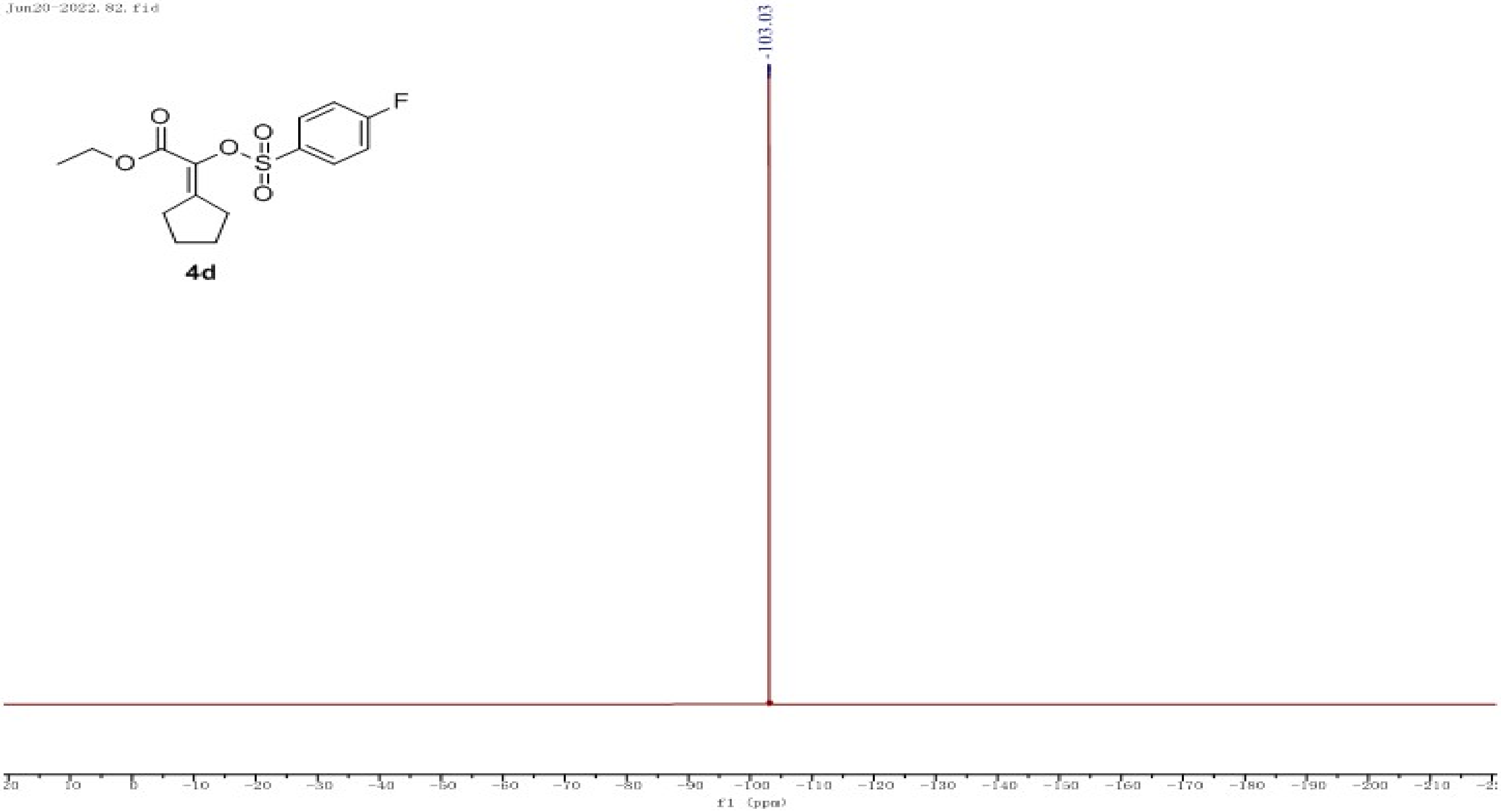

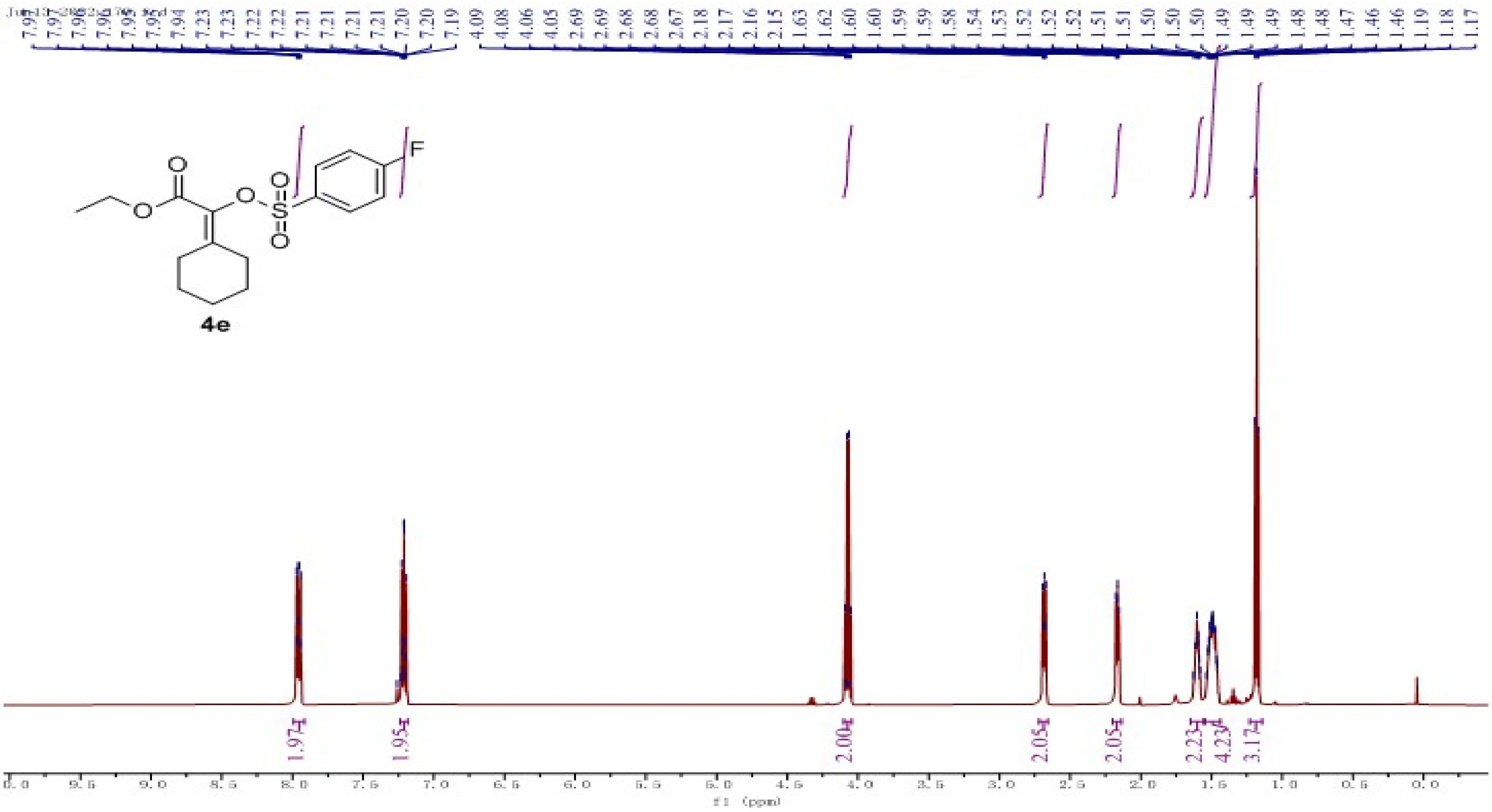
7.97
7.97
7.96
7.96
7.95
7.95
7.94
7.23
7.23
7.22
7.22
7.21
7.21
7.21
7.21
7.20
7.20
7.19
4.09
4.08
4.06
4.05
2.69
2.69
2.68
2.68
2.67
2.18
2.17
2.16
2.15
1.63
1.62
1.60
1.60
1.59
1.59
1.58
1.54
1.53
1.52
1.52
1.52
1.51
1.51
1.50
1.50
1.50
1.49
1.49
1.49
1.48
1.48
1.47
1.46
1.46
1.19
1.19
1.18
1.17
F
O
S
4e
1.97
1.95
2.00
2.05
2.05
2.23
4.23
3.17
10.0
9.5
9.0
8.5
8.0
7.5
7.0
6.5
6.0
5.5
5.0
4.5
4.0
3.5
3.0
2.5
2.0
1.5
1.0
0.5
0.0
f1 (ppm)

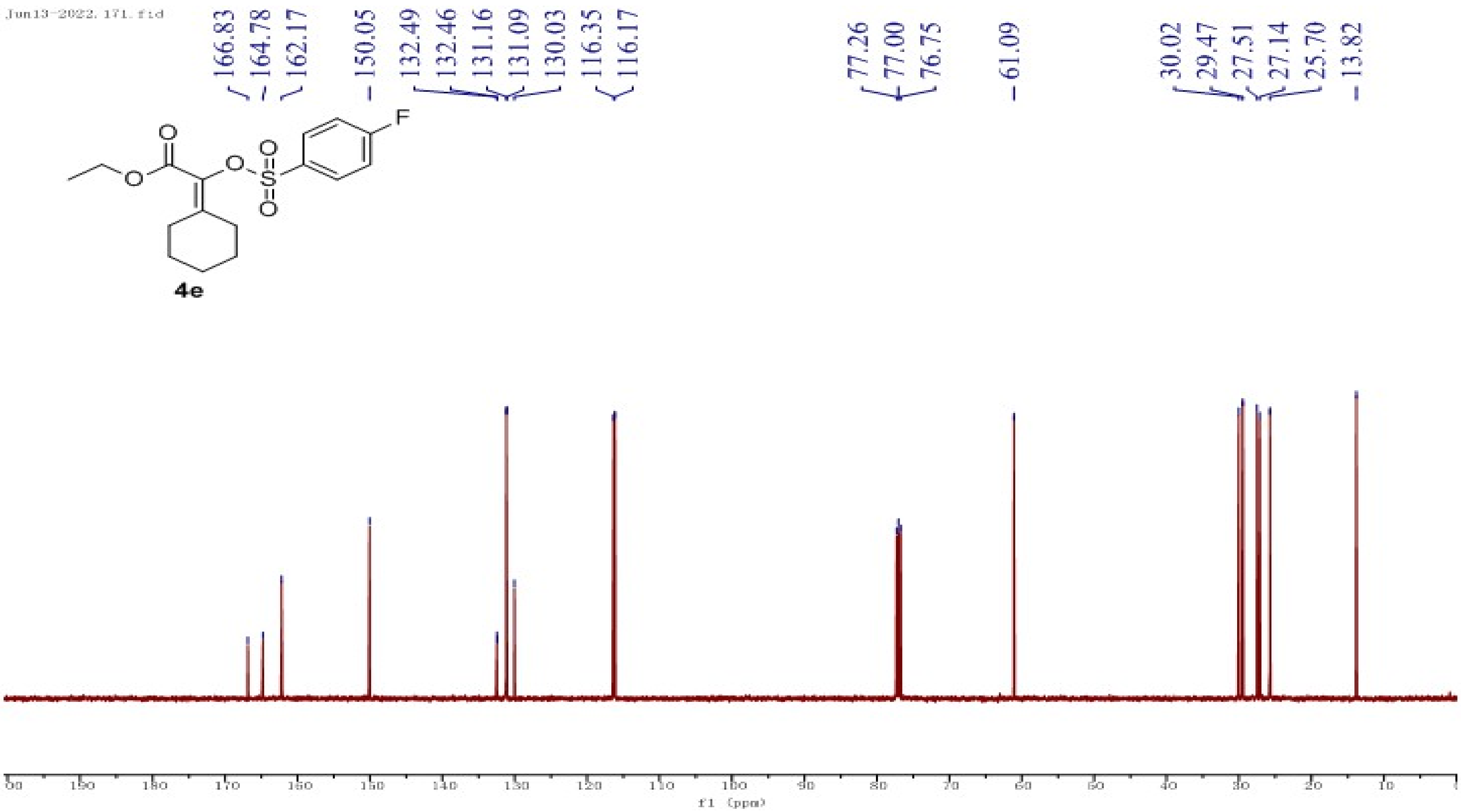
Jun13-2022.171.fid
166.83
164.78
162.17
150.05
132.49
132.46
131.16
131.09
130.03
116.35
116.17
77.26
77.00
76.75
61.09
30.02
29.47
27.51
27.14
25.70
13.82
F
O
S
4e
f1 (ppm)

Jun13-2022.172.fid

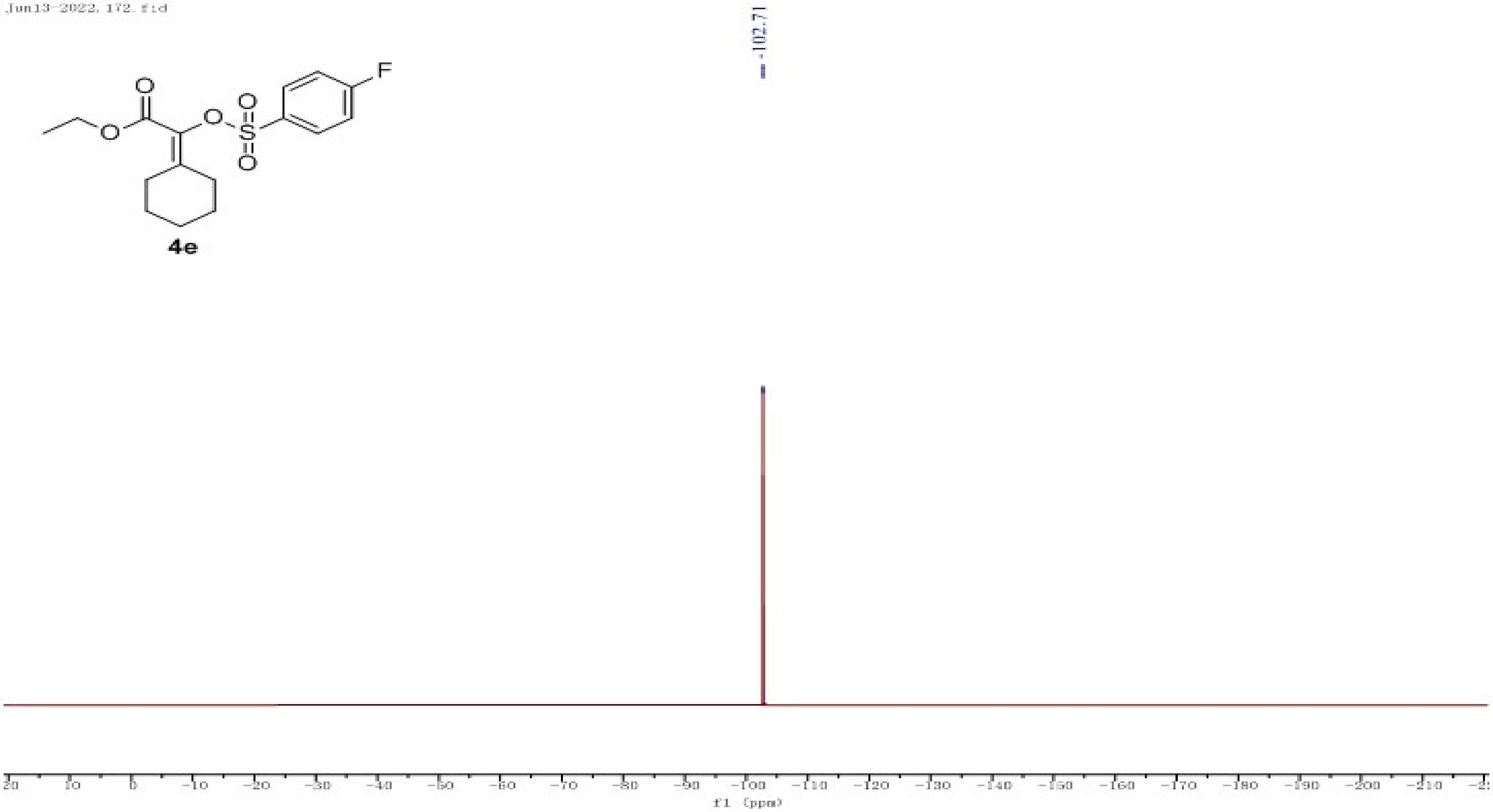

4e

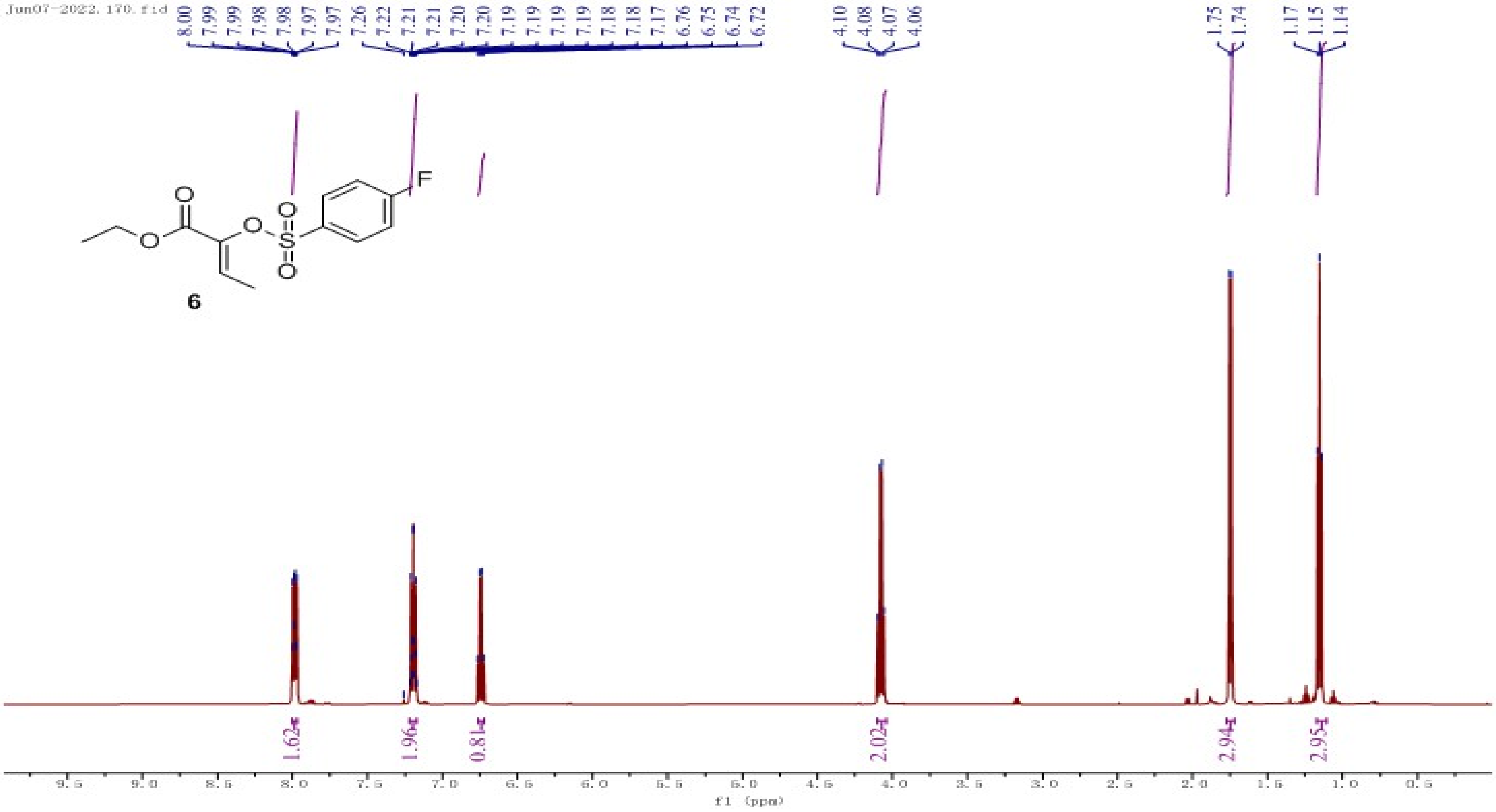
Jun07-2022.170.fid
8.00 7.99 7.99 7.98 7.98 7.97 7.97 7.26 7.22 7.21 7.21 7.20 7.20 7.19 7.19 7.19 7.19 7.18 7.18 7.17 6.76 6.75 6.74 6.72
4.10 4.08 4.07 4.06
1.75 1.74
1.17 1.15 1.14
6
1.62
1.96
0.81
2.02
2.94
2.95
9.5 9.0 8.5 8.0 7.5 7.0 6.5 6.0 5.5 5.0 4.5 4.0 3.5 3.0 2.5 2.0 1.5 1.0 0.5
f1 (ppm)

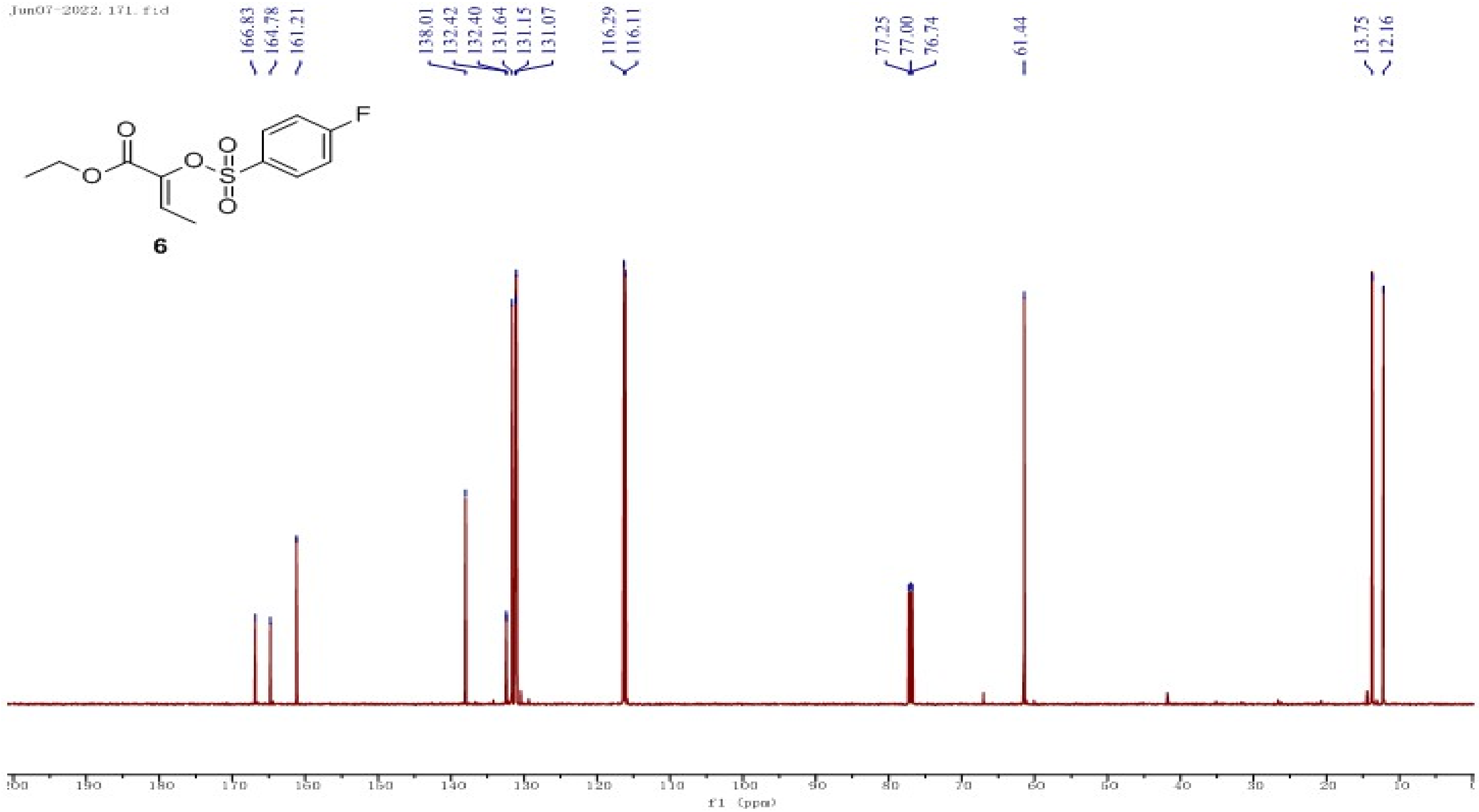
Jun07-2022.171.fid
166.83
164.78
161.21
138.01
132.42
132.40
131.64
131.15
131.07
116.29
116.11
77.25
77.00
76.74
61.44
13.75
12.16
F
6
f1 (ppm)

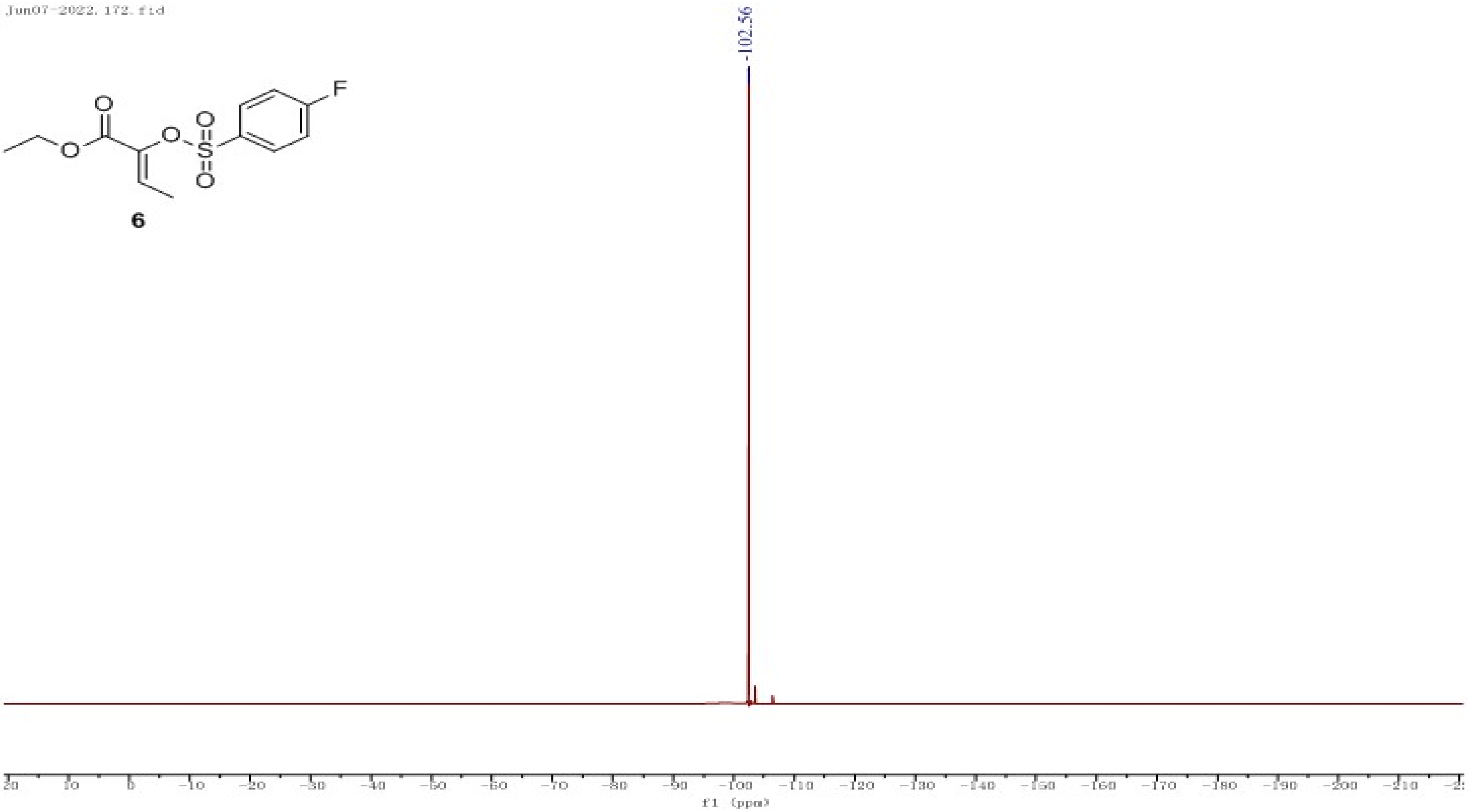
Jun07-2022.172.fid
-102.56
6
f1 (ppm)

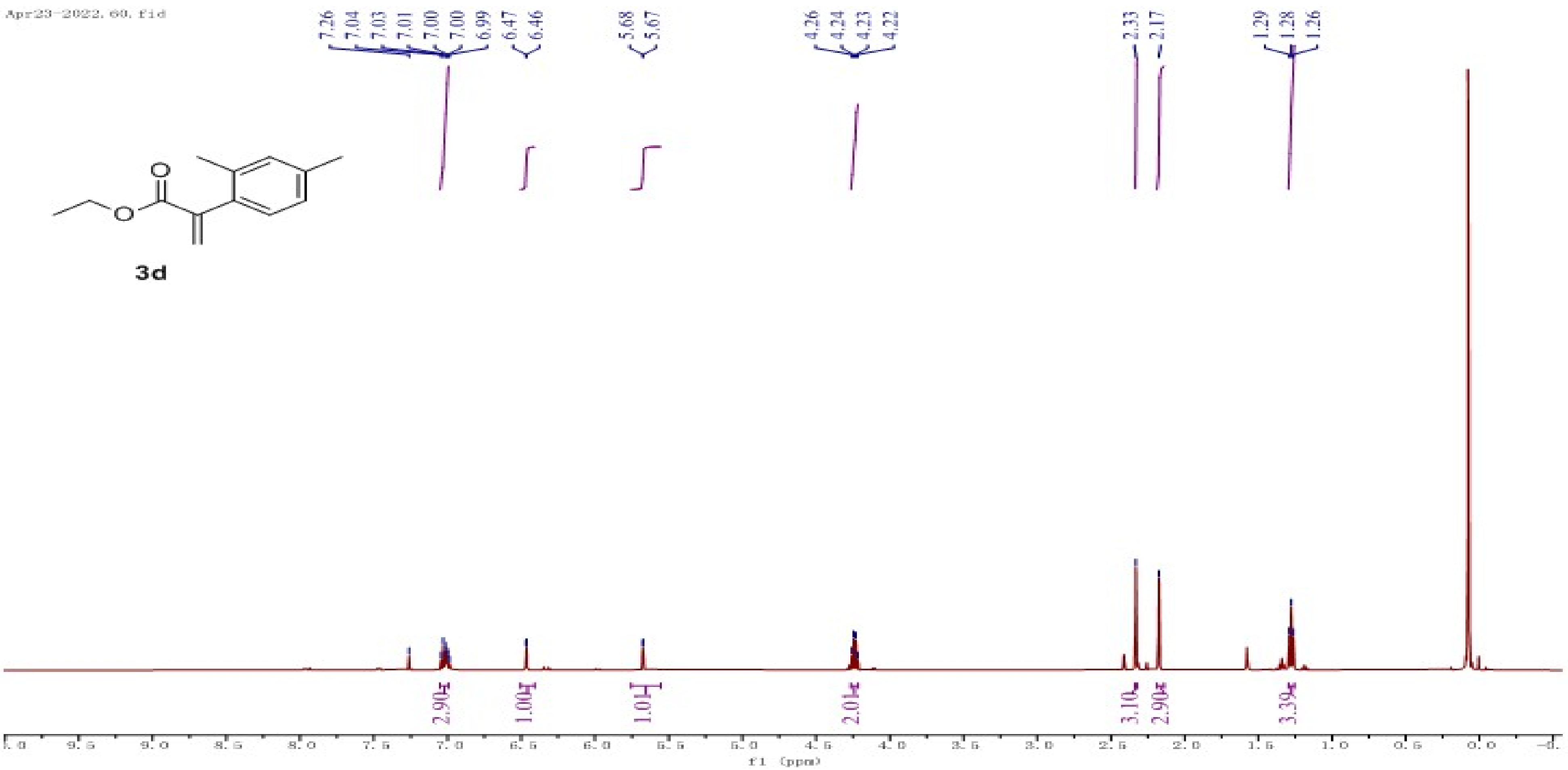
Apr23-2022.60.fid
7.26
7.04
7.03
7.01
7.00
7.00
6.99
6.47
6.46
5.68
5.67
4.26
4.24
4.23
4.22
2.33
2.17
1.29
1.28
1.26
3d
2.90
1.00
1.01
2.01
3.10
2.90
3.39
f1 (ppm)

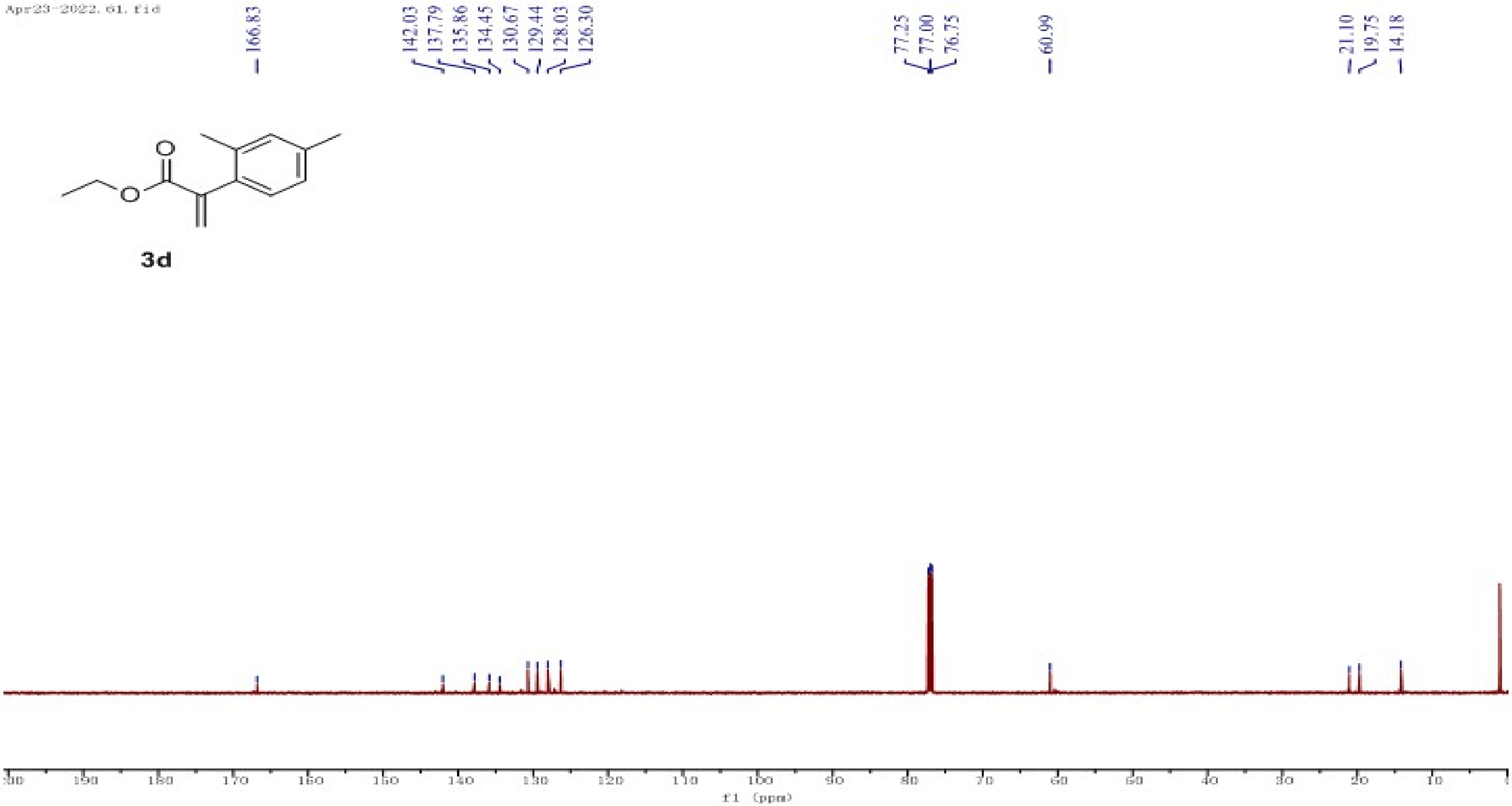
Apr23-2022.61.fid
166.83
142.03
137.79
135.86
134.45
130.67
129.44
128.03
126.30
77.25
77.00
76.75
60.99
21.10
19.75
14.18
3d
f1 (ppm)

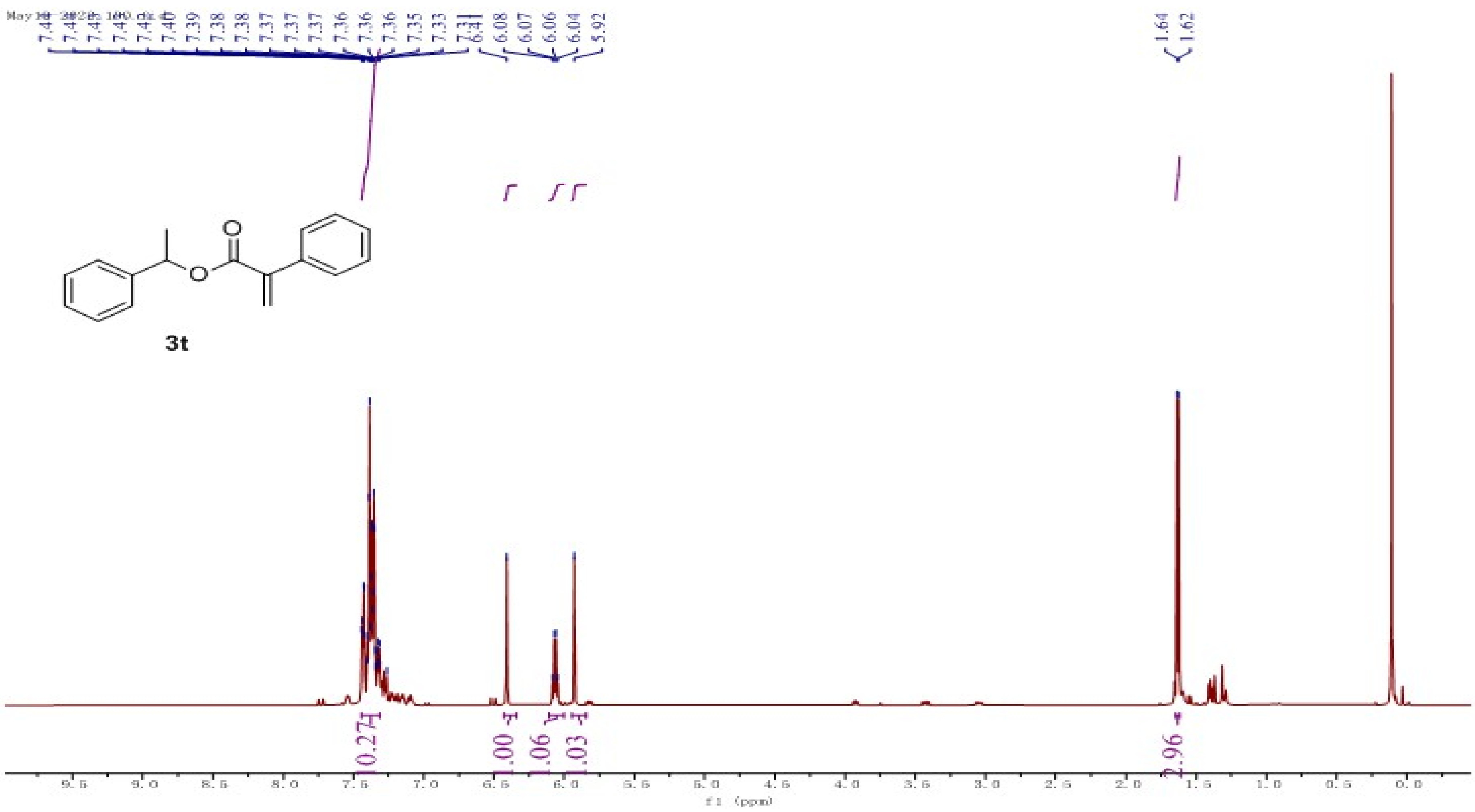

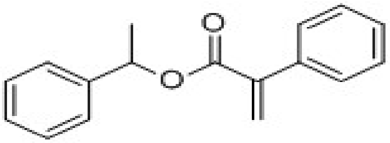

3t

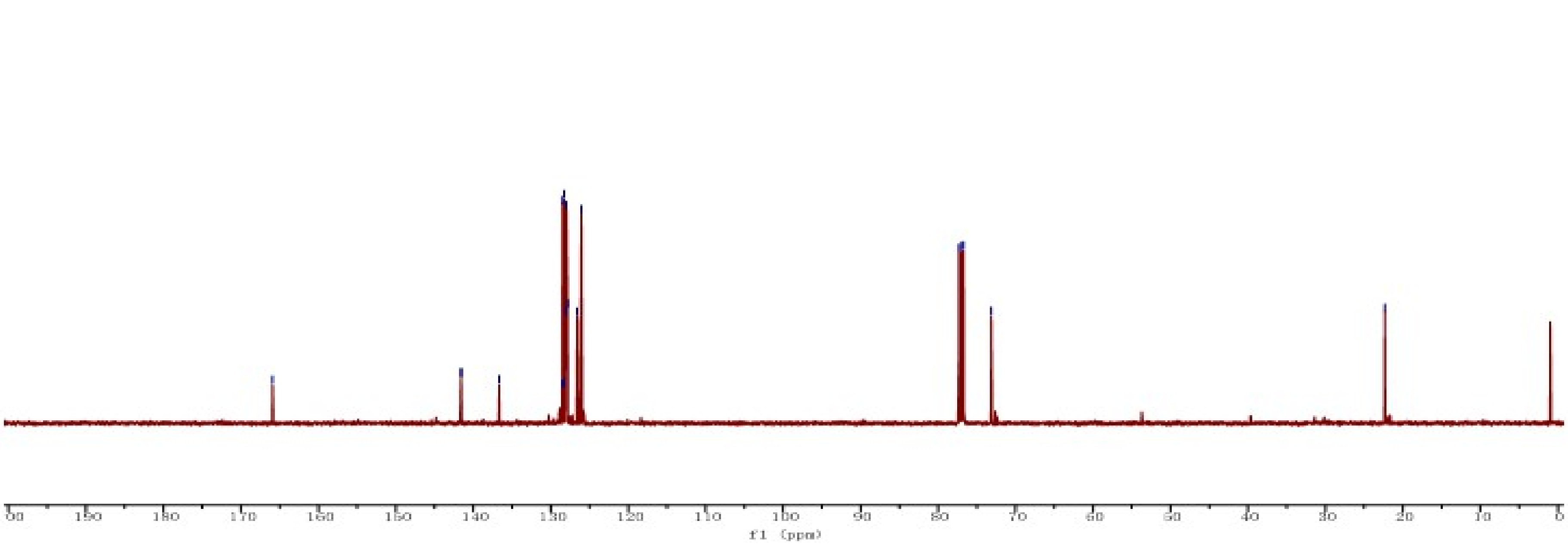
May19-2022.131.fid
165.94
141.58
141.56
136.67
128.50
128.48
128.29
128.27
128.08
128.01
127.85
126.59
126.03
77.25
77.00
76.74
73.13
22.34
f1 (ppm)

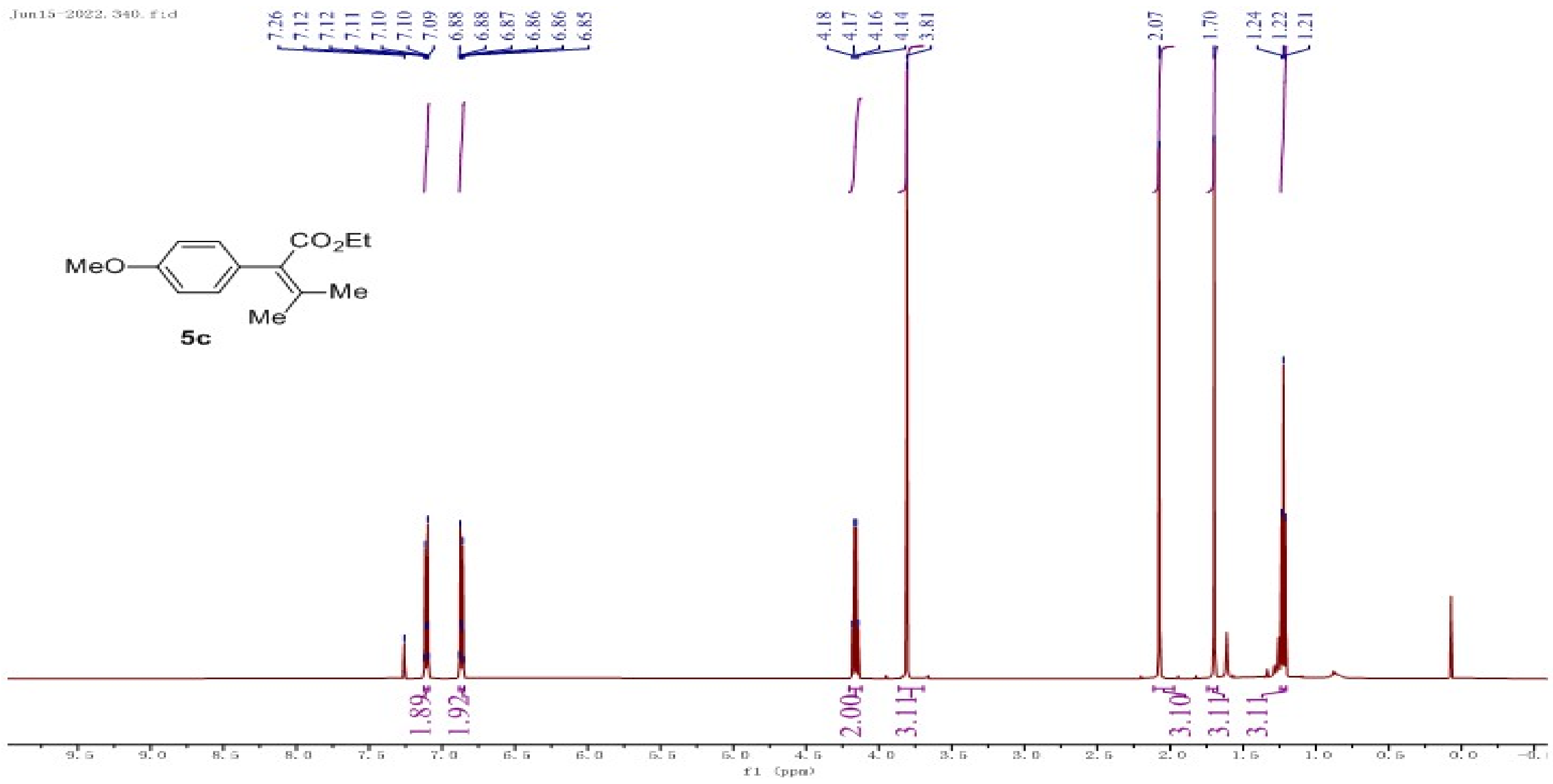
Jun15-2022.340.fid
7.26 7.12 7.12 7.11 7.10 7.10 7.09 6.88 6.88 6.87 6.86 6.86 6.85
4.18 4.17 4.16 4.14 3.81
2.07 1.70 1.24 1.22 1.21
CO2Et
MeO
Me
Me
5c
1.89 1.92 2.00 3.11 3.10 3.11 3.11
9.5 9.0 8.5 8.0 7.5 7.0 6.5 6.0 5.5 5.0 4.5 4.0 3.5 3.0 2.5 2.0 1.5 1.0 0.5 0.0
f1 (ppm)

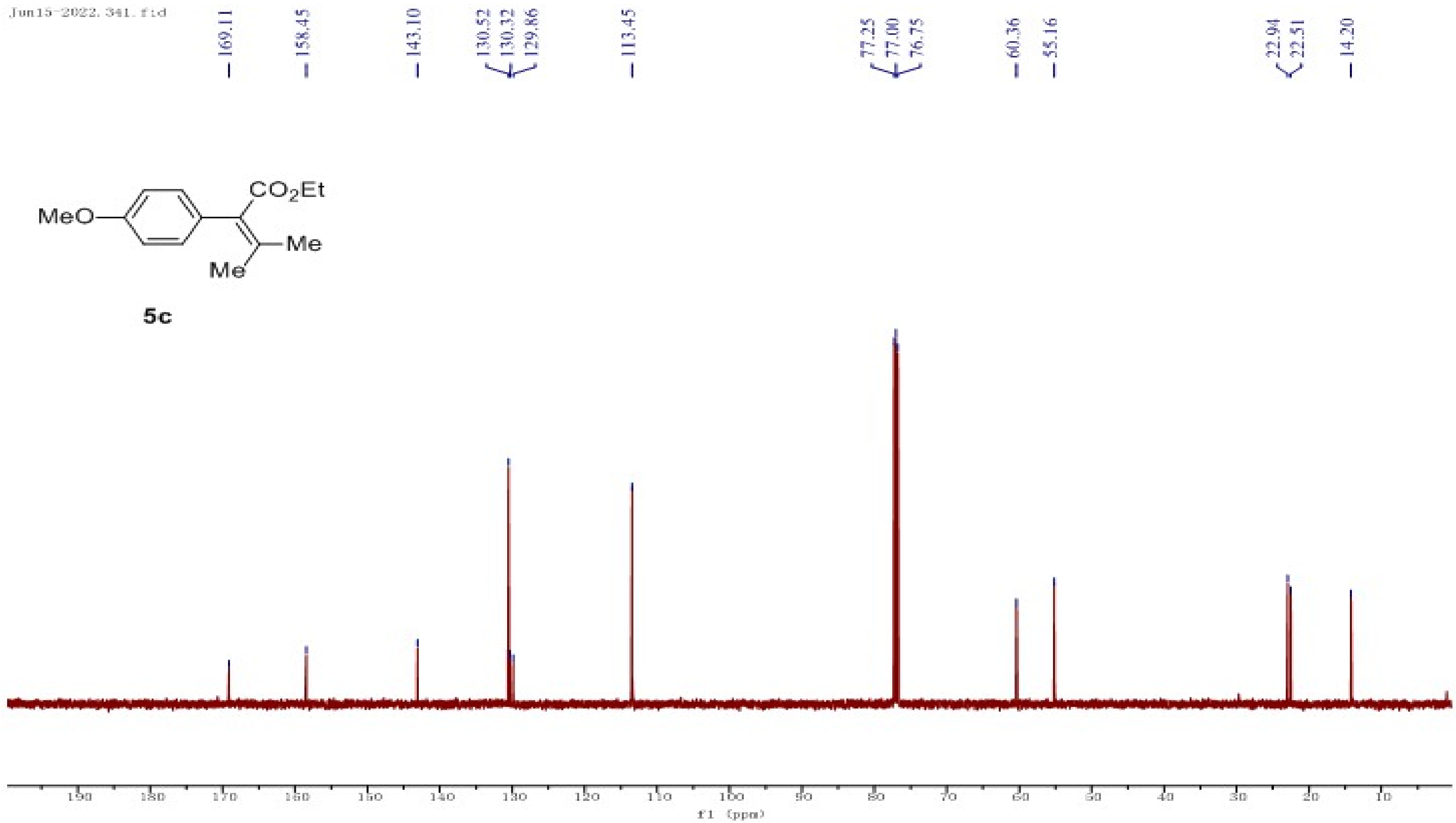
Jun15-2022.341.fid
169.11
158.45
143.10
130.52
130.32
129.86
113.45
77.25
77.00
76.75
60.36
55.16
22.94
22.51
14.20
MeO
CO2Et
Me
Me
5c
190
180
170
160
150
140
130
120
110
100
90
80
70
60
50
40
30
20
10
f1 (ppm)

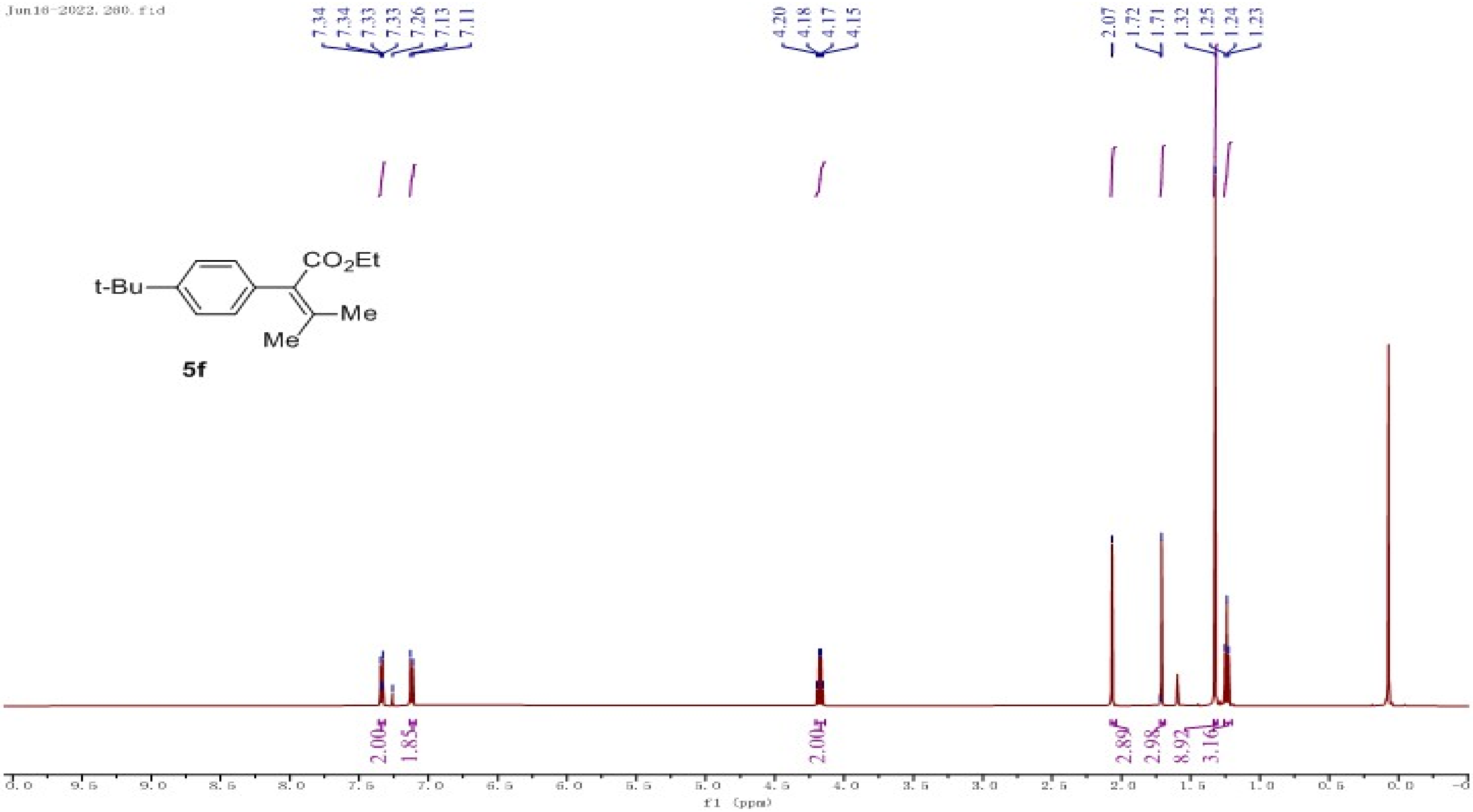
Jun16-2022.260.fid
7.34
7.34
7.33
7.33
7.26
7.13
7.11
4.20
4.18
4.17
4.15
2.07
1.72
1.71
1.32
1.25
1.24
1.23
t-Bu
CO2Et
Me
Me
5f
2.00
1.85
2.00
2.89
2.98
8.92
3.16
f1 (ppm)

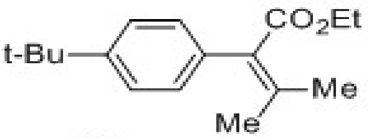
t-Bu
CO_2Et
Me
Me

5f

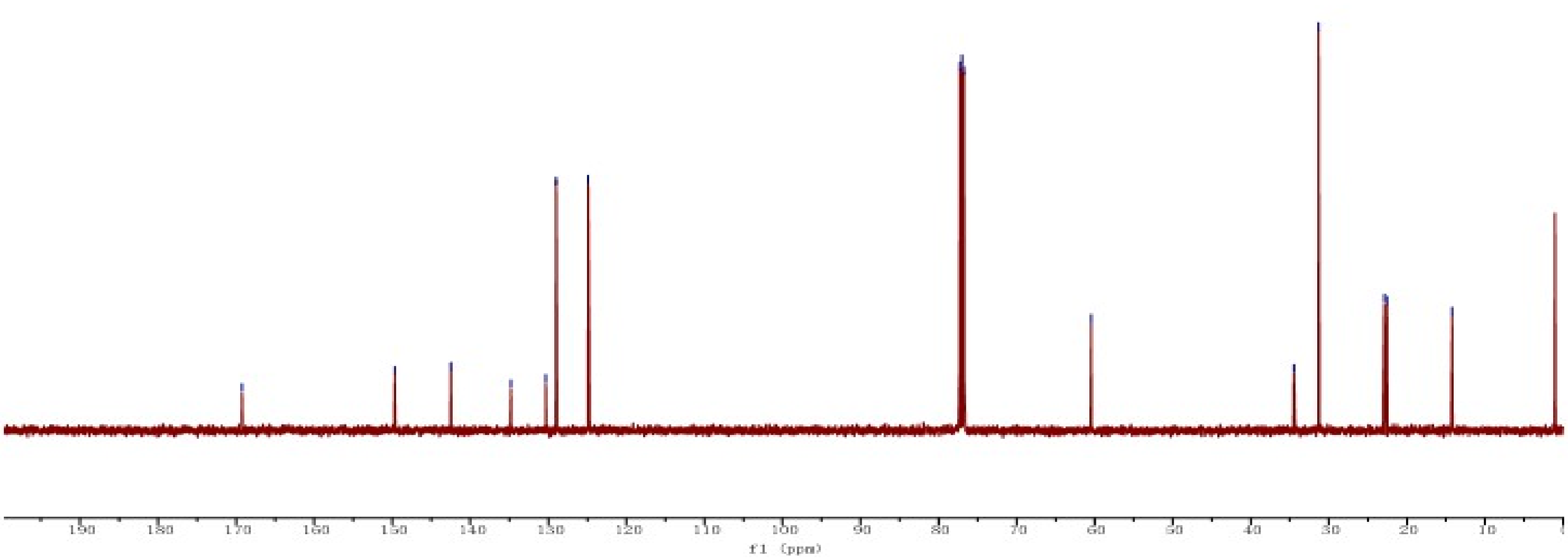
Jun16-2022.261.fid
169.22
149.67
142.47
134.77
130.32
128.98
124.92
77.25
77.00
76.75
60.40
34.47
31.33
22.87
22.55
14.21
190 180 170 160 150 140 130 120 110 100 90 80 70 60 50 40 30 20 10
f1 (ppm)

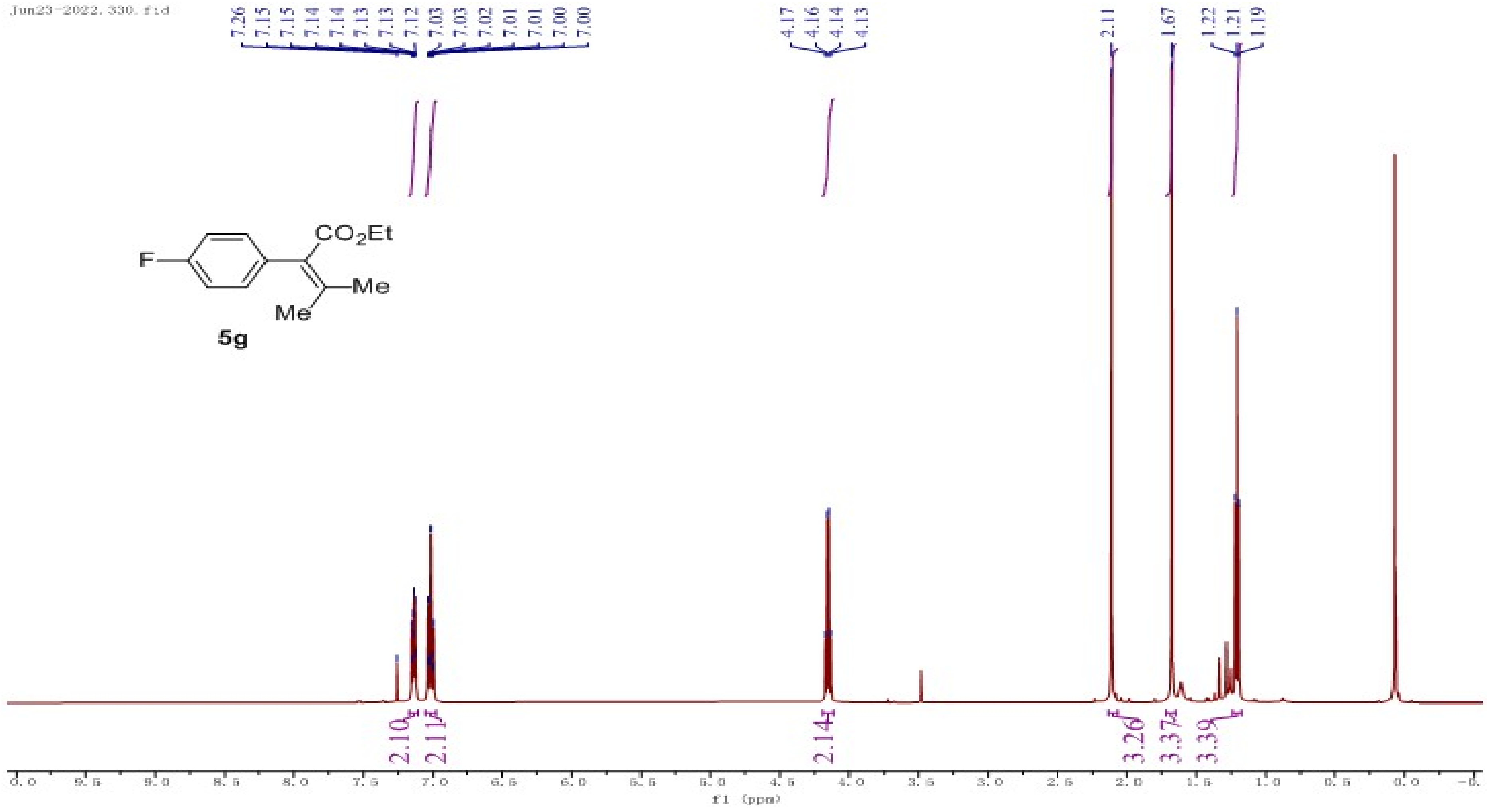
Jun23-2022.330.fid
7.26
7.15
7.15
7.14
7.14
7.13
7.13
7.12
7.03
7.03
7.02
7.01
7.01
7.00
7.00
4.17
4.16
4.14
4.13
2.11
1.67
1.22
1.21
1.19
F
CO2Et
Me
Me
5g
2.10
2.11
2.14
3.26
3.37
3.39
f1 (ppm)

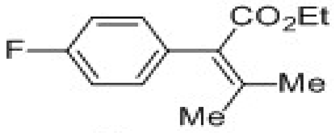
F
CO2Et
Me
Me

5g

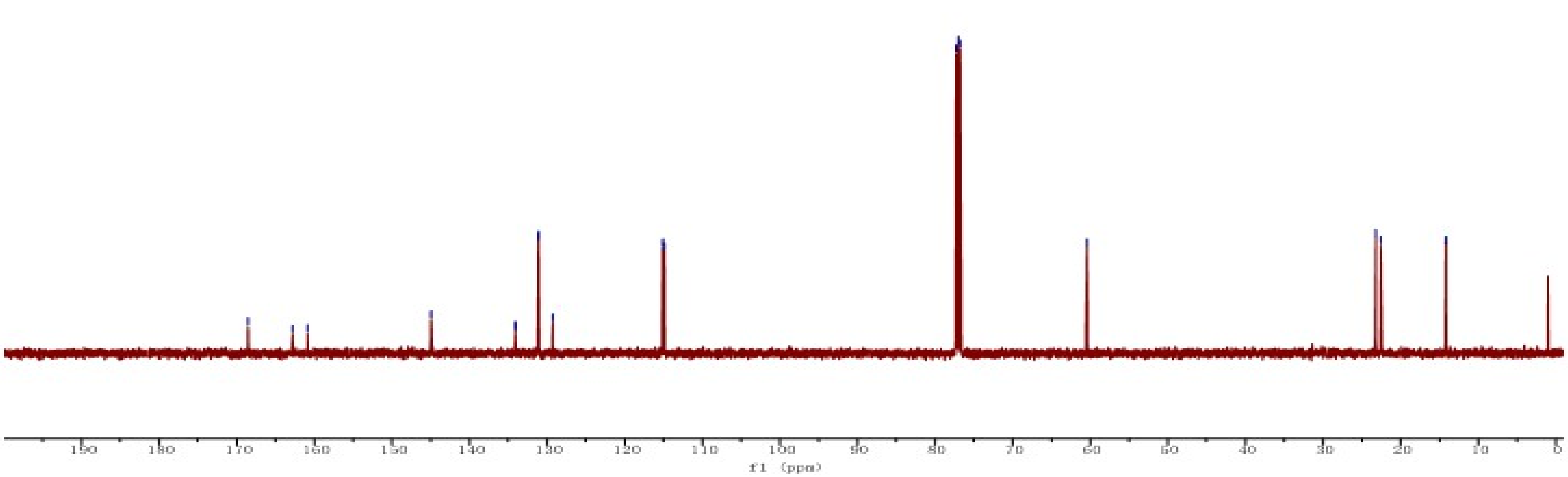
Jun23-2022.331.fid
168.49
162.78
160.83
144.94
134.05
134.03
131.12
131.06
129.25
115.05
114.88
77.26
77.00
76.75
60.43
23.20
22.52
14.16
190
180
170
160
150
140
130
120
110
100
90
80
70
60
50
40
30
20
10
0
f1 (ppm)

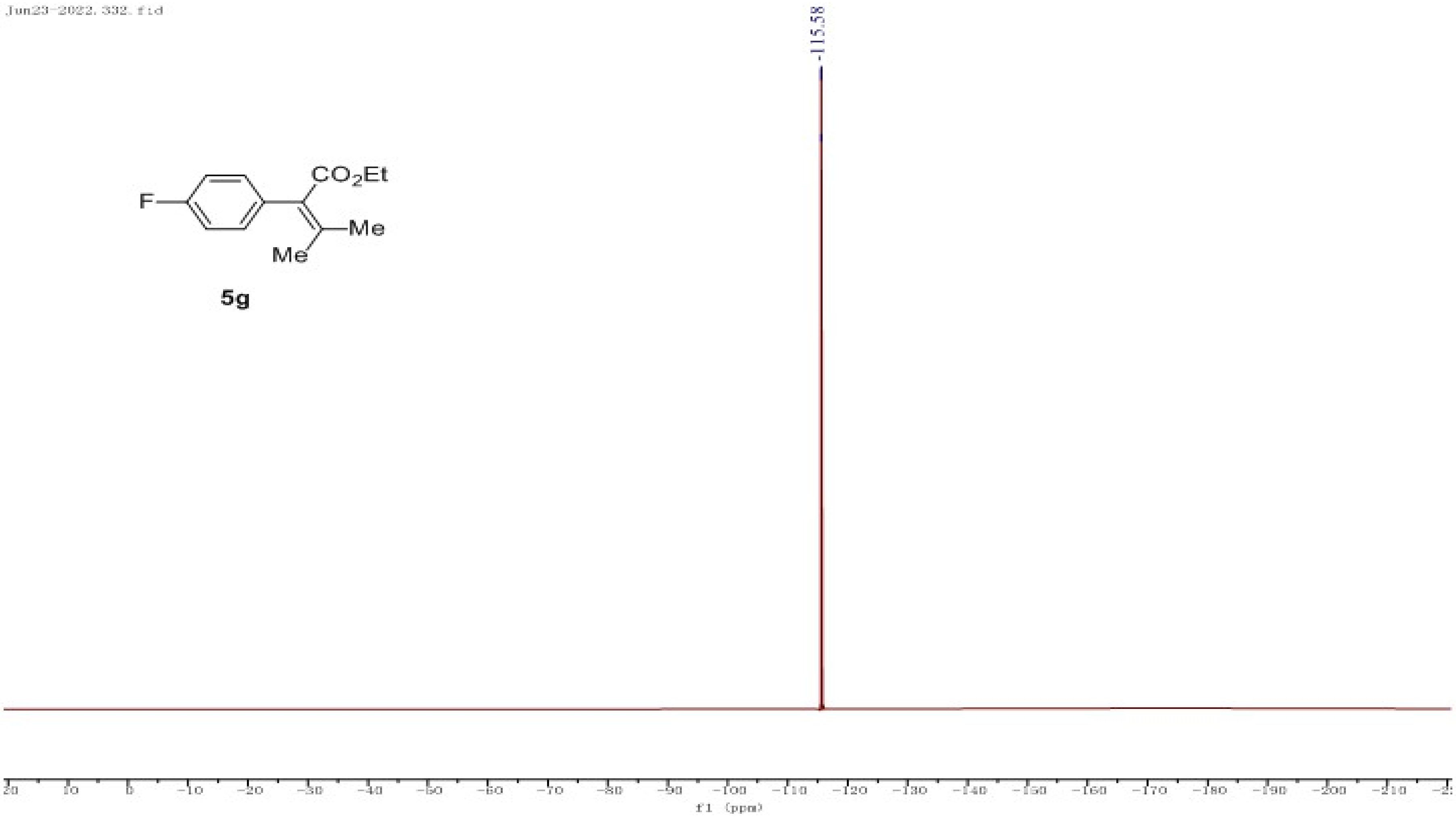
Jun23-2022.332.fid
-115.58
F
CO2Et
Me
Me
5g
f1 (ppm)

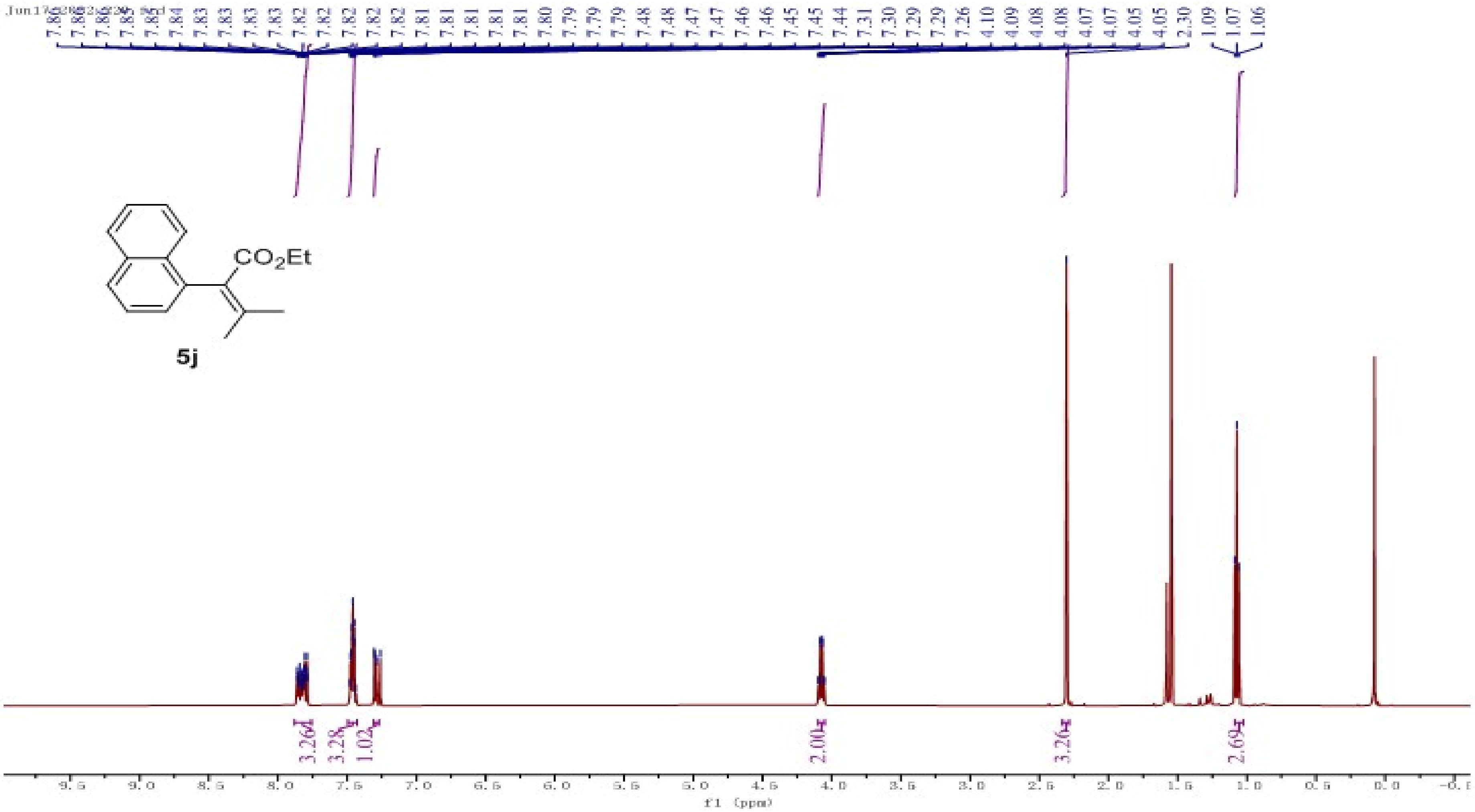

CO2Et
5j
f1 (ppm)

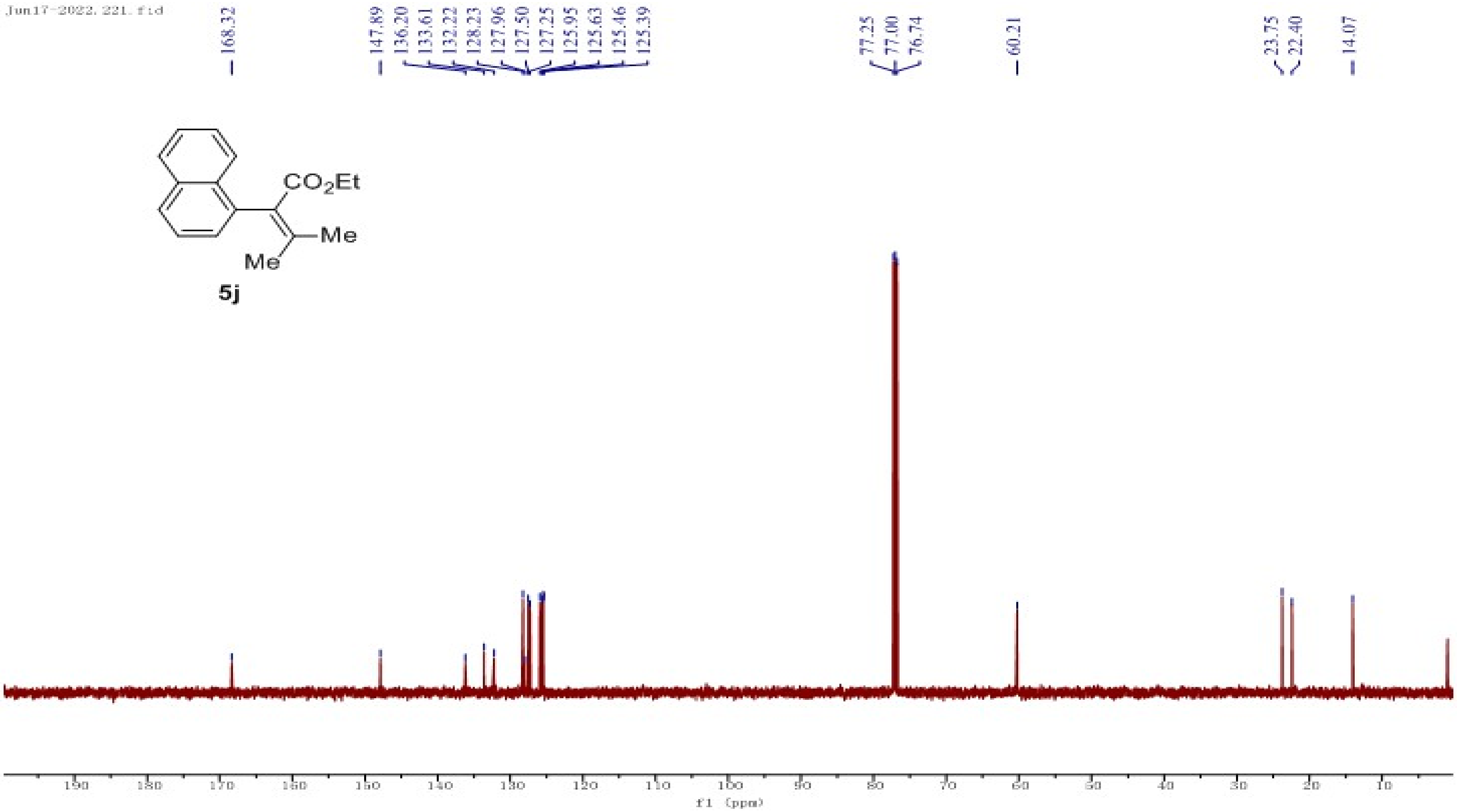

Jun17-2022.221.fid
168.32
147.89
136.20
133.61
132.22
128.23
127.96
127.50
127.25
125.95
125.63
125.46
125.39
77.25
77.00
76.74
60.21
23.75
22.40
14.07
CO2Et
Me
Me
5j
190
180
170
160
150
140
130
120
110
100
90
80
70
60
50
40
30
20
10
f1 (ppm)

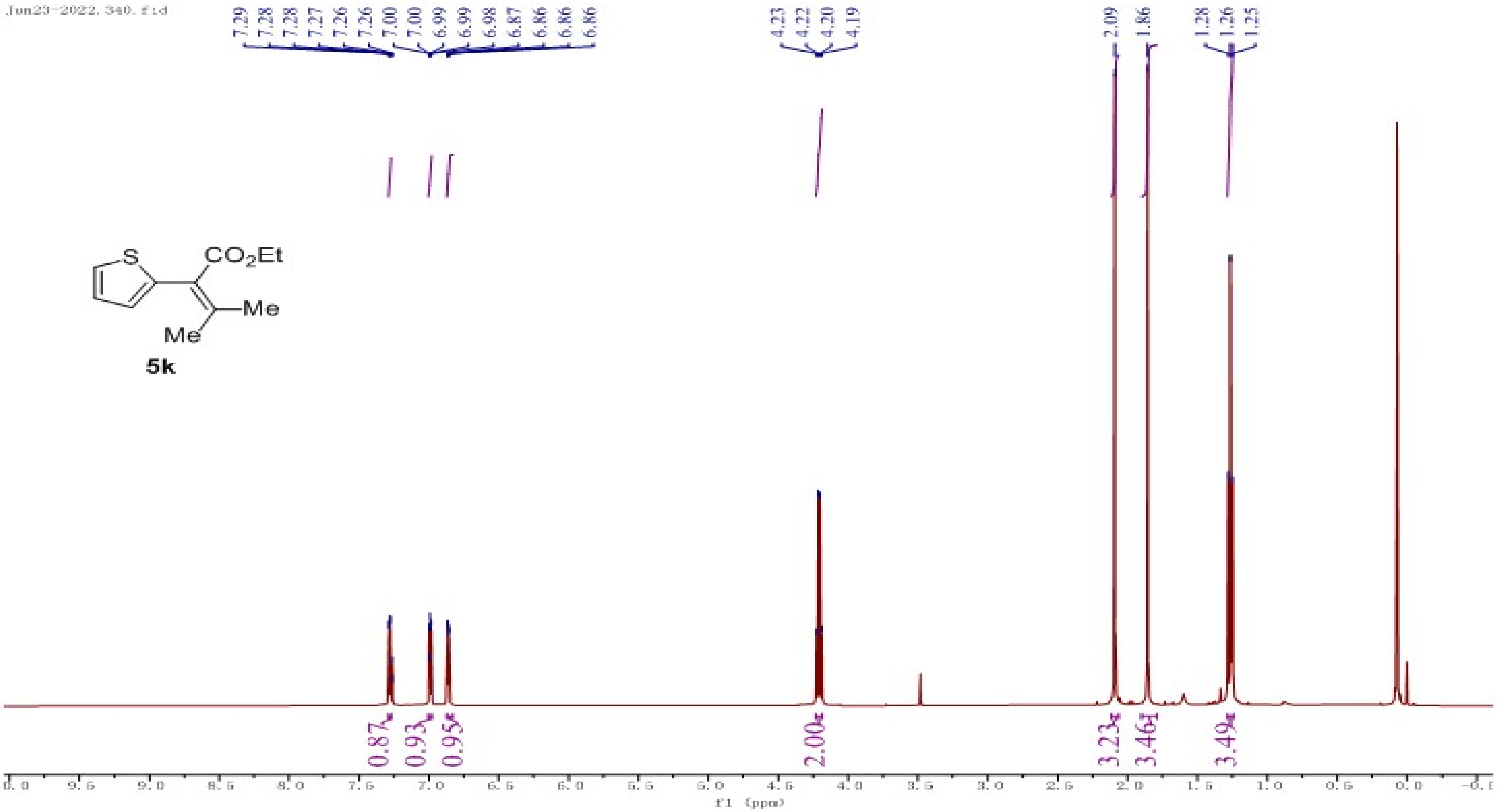

Jun23-2022.340.fid
7.29
7.28
7.28
7.27
7.26
7.26
7.00
7.00
6.99
6.99
6.98
6.87
6.86
6.86
6.86
4.23
4.22
4.20
4.19
2.09
1.86
1.28
1.26
1.25
CO2Et
S
Me
Me
0.87
0.93
0.95
2.00
3.23
3.46
3.49
f1 (ppm)

5k

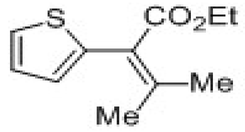
S
CO2Et
Me
Me

5k

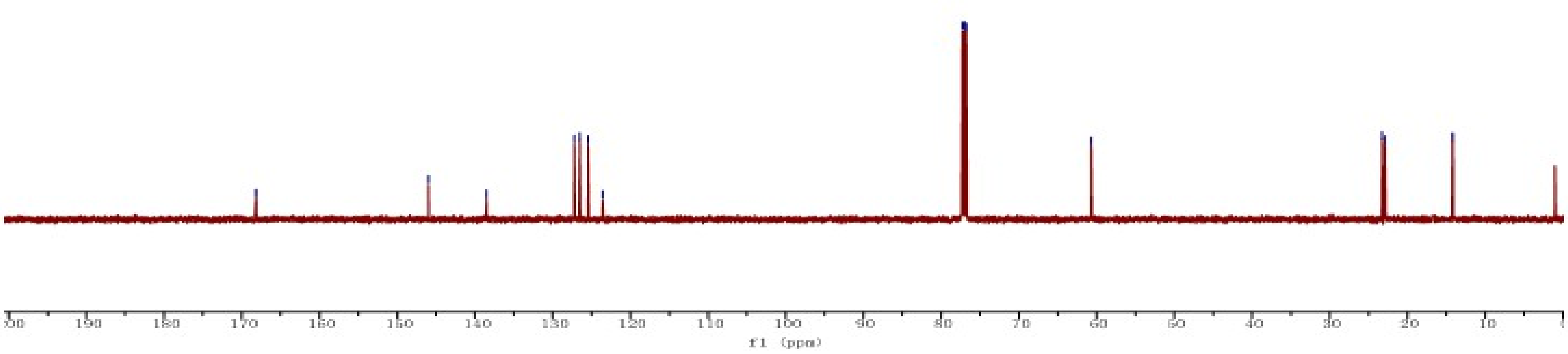
Jun23-2022.341.fid
168.20
146.00
138.51
127.24
126.47
125.48
123.57
77.25
77.00
76.74
60.74
23.26
22.89
14.14
f1 (ppm)

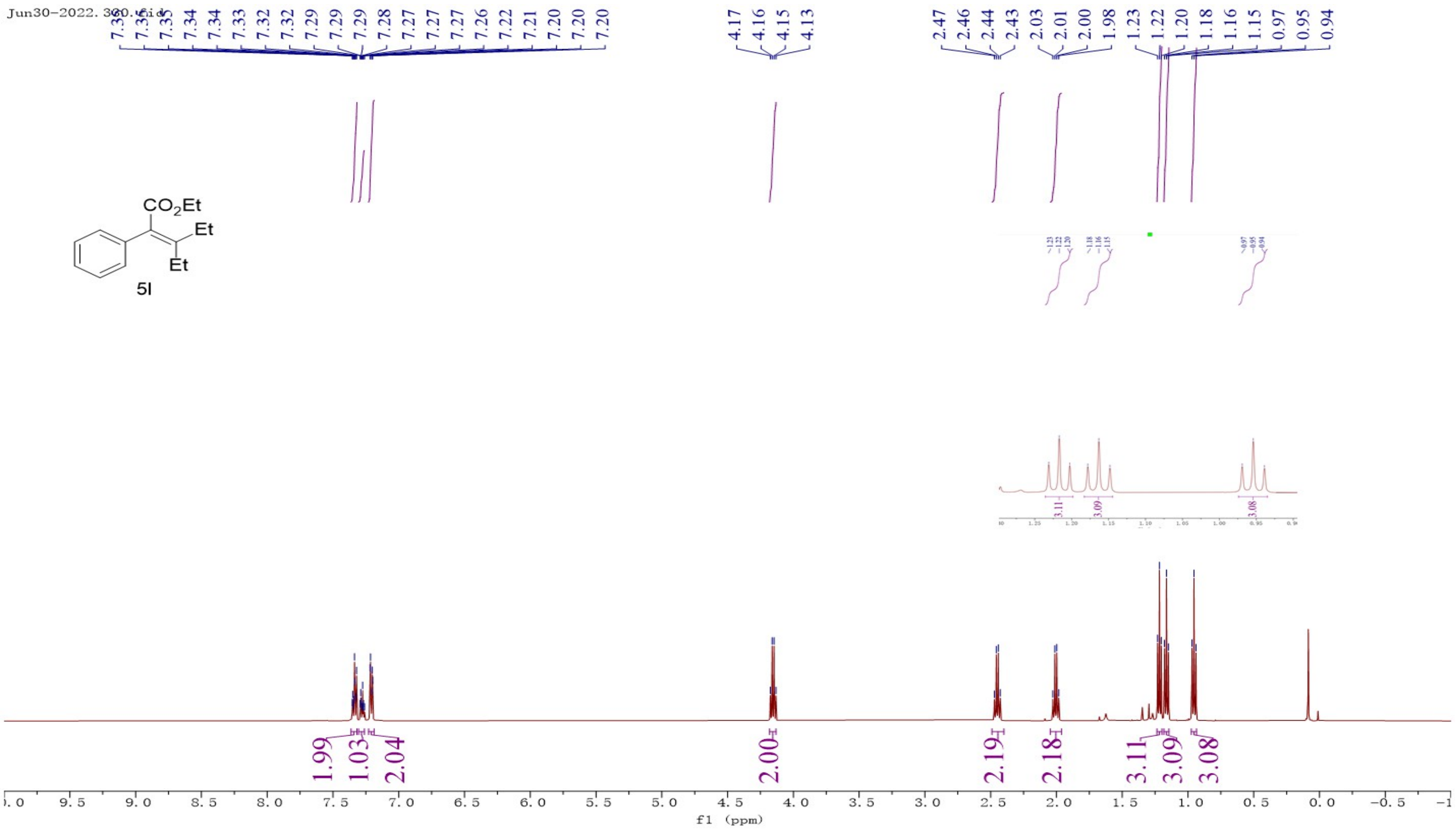
CO2Et
Et
Et
5l
f1 (ppm)

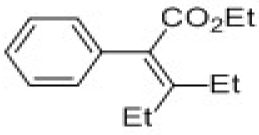

5l

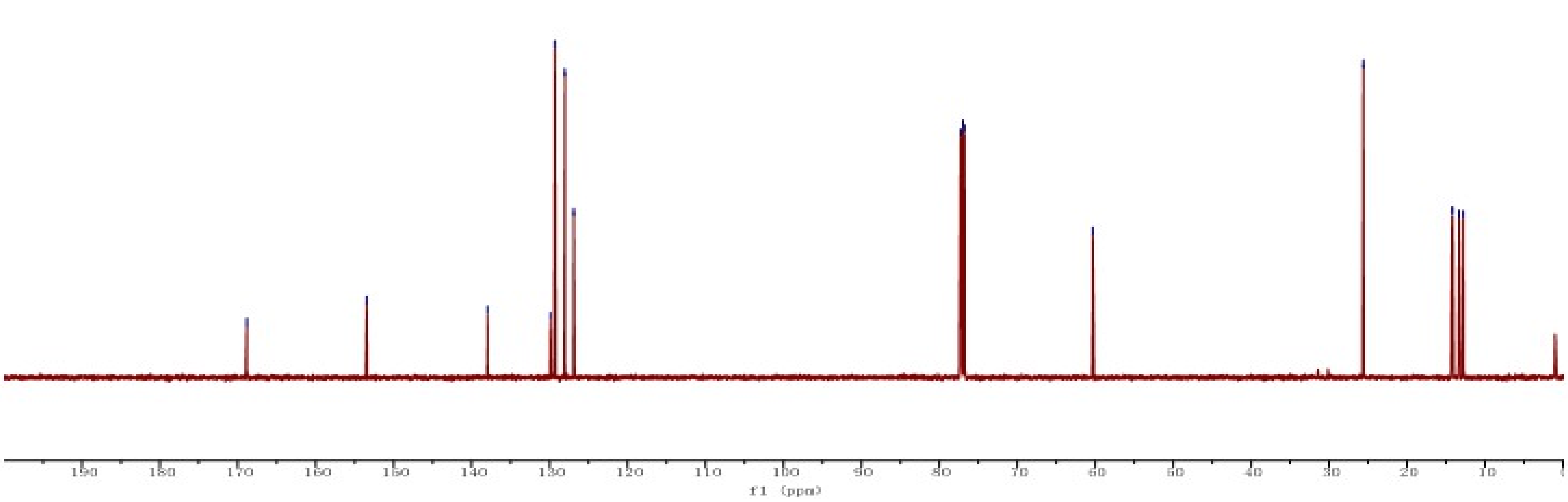
Jun30-2022.331.fid
168.83
153.46
137.93
129.88
129.29
128.02
126.90
77.26
77.00
76.75
60.33
25.64
14.13
13.32
12.73
f1 (ppm)

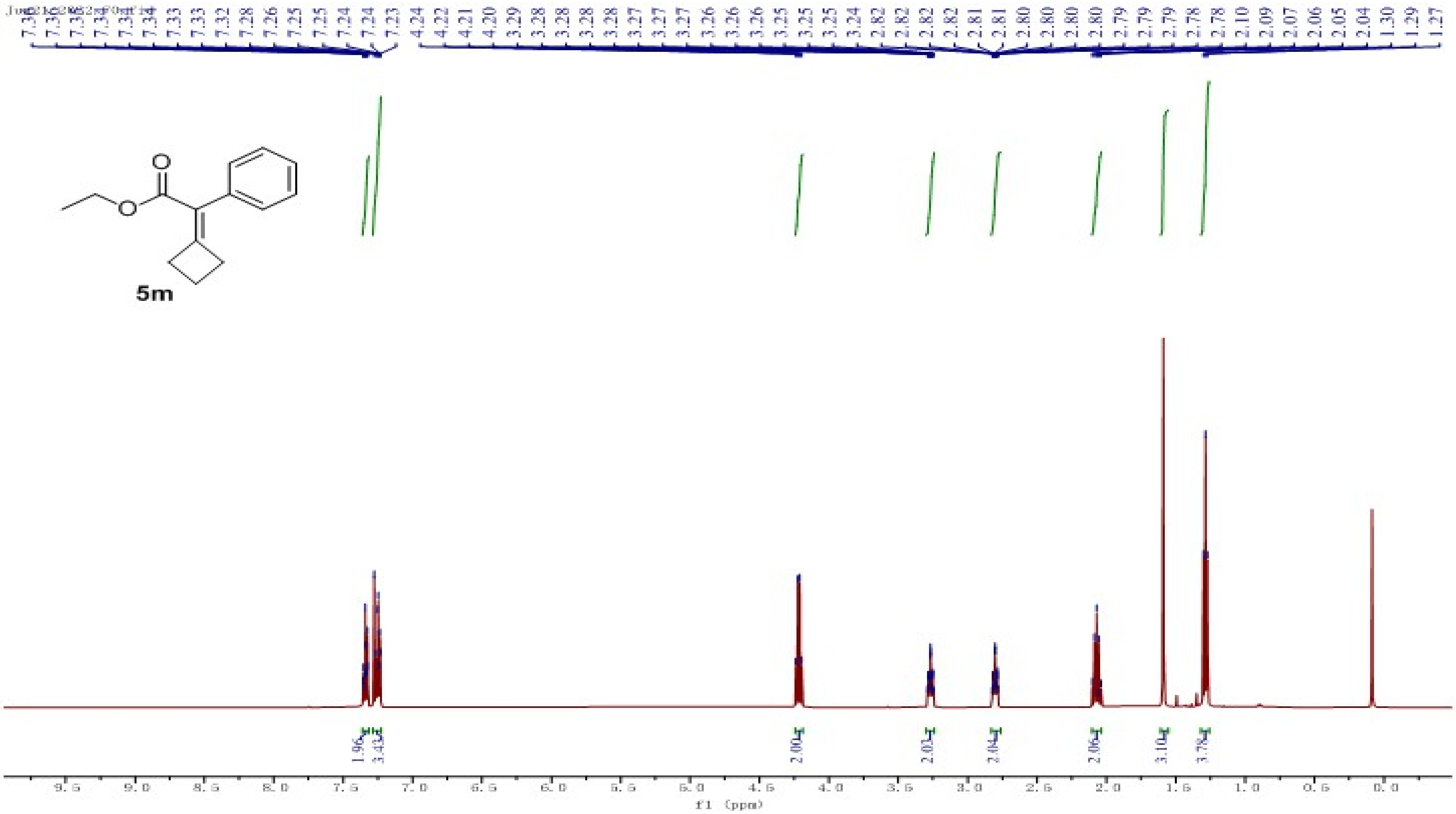

5m

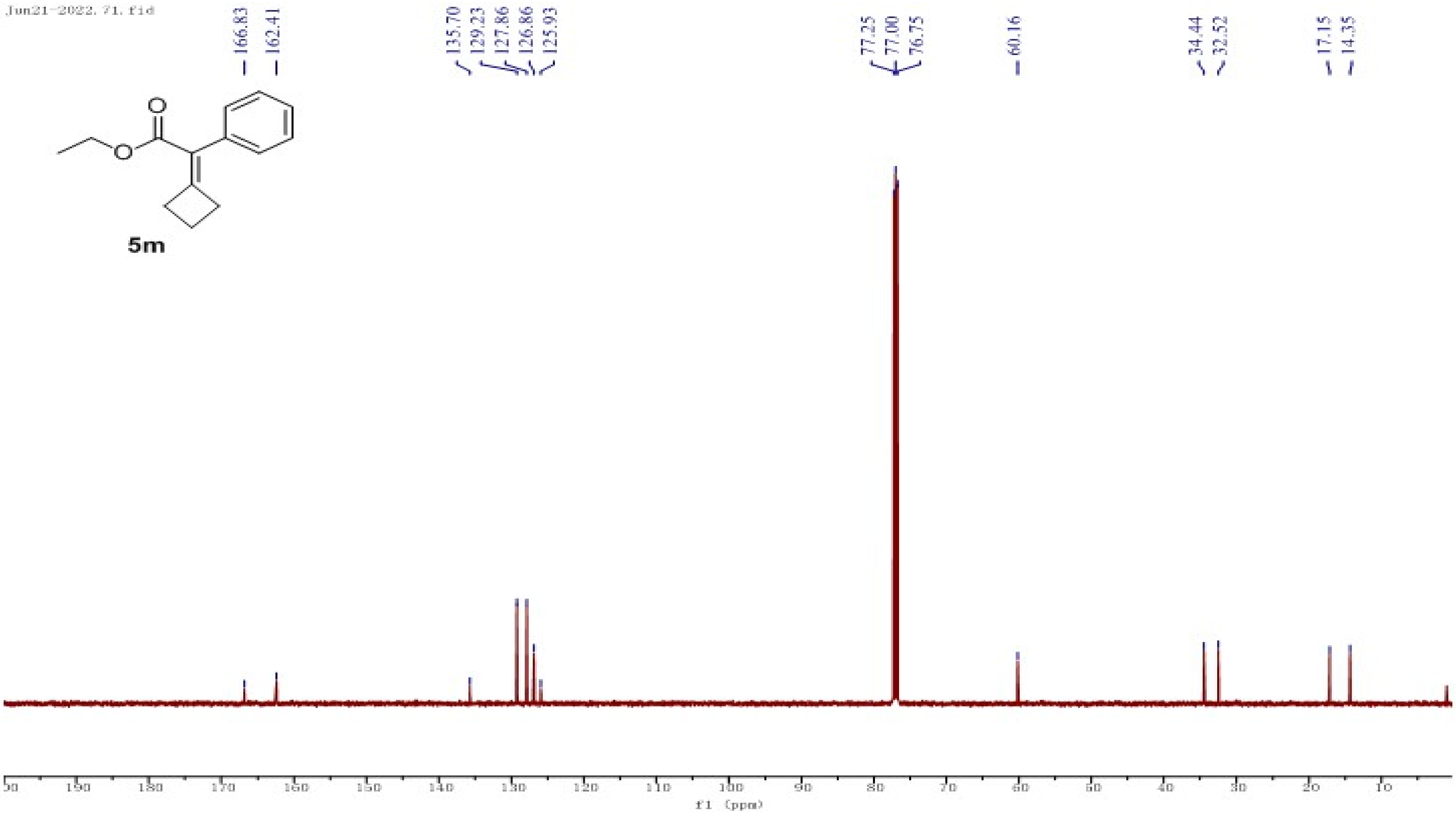
Jun21-2022.71.fid
166.83
162.41
135.70
129.23
127.86
126.86
125.93
77.25
77.00
76.75
60.16
34.44
32.52
17.15
14.35
f1 (ppm)

5m

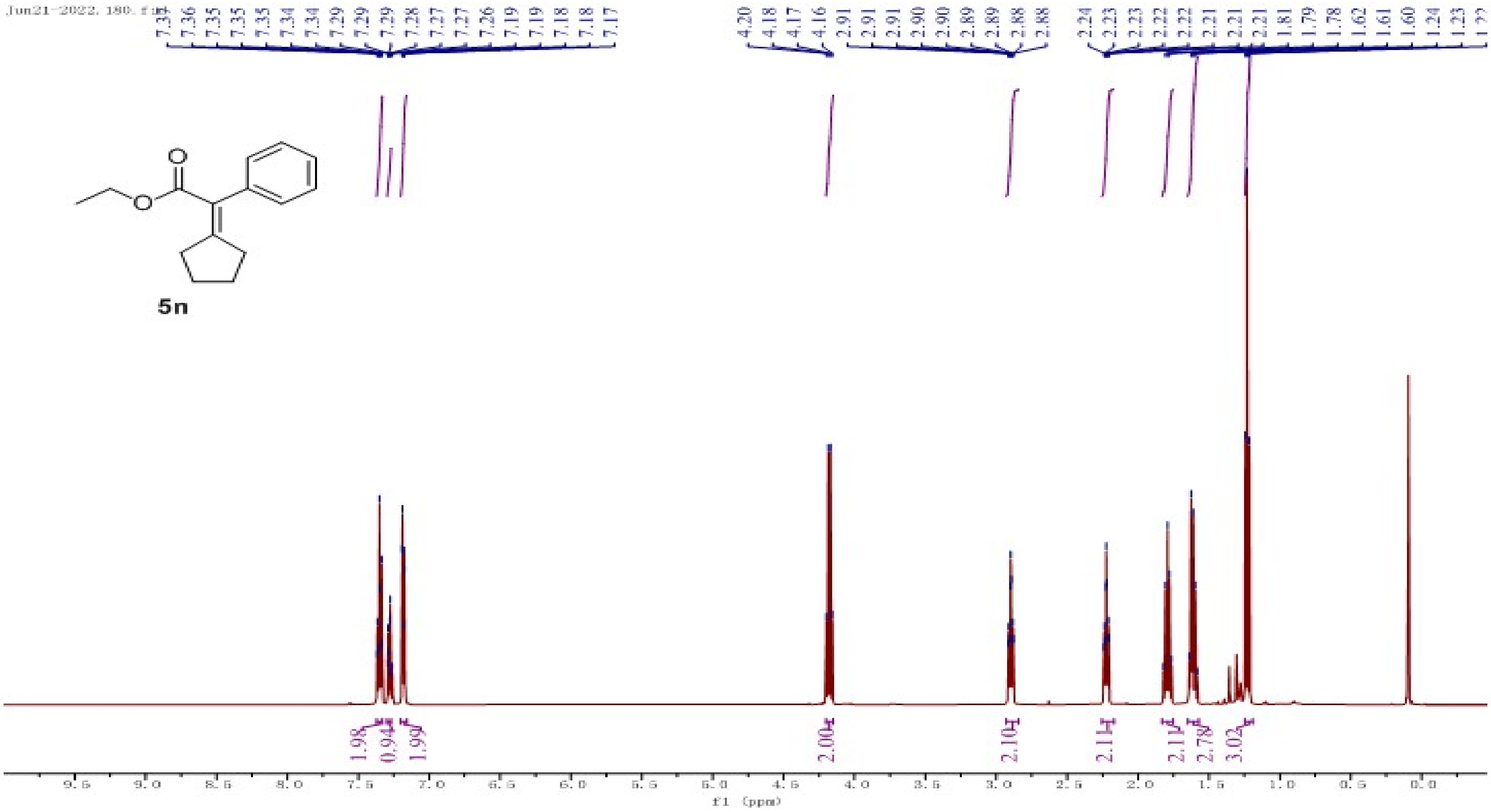
Jun21-2022.180.fid
7.37 7.36 7.35 7.35 7.35 7.34 7.34 7.29 7.29 7.29 7.28 7.27 7.27 7.26 7.19 7.19 7.18 7.18 7.17
4.20 4.18 4.17 4.16 2.91 2.91 2.91 2.90 2.90 2.89 2.89 2.88 2.88
2.24 2.23 2.23 2.22 2.22 2.21 2.21 2.21 1.81 1.79 1.78 1.62 1.61 1.60 1.24 1.23 1.22
5n
1.98 0.94 1.99 2.00 2.10 2.11 2.11 2.78 3.02
9.5 9.0 8.5 8.0 7.5 7.0 6.5 6.0 5.5 5.0 4.5 4.0 3.5 3.0 2.5 2.0 1.5 1.0 0.5 0.0
f1 (ppm)

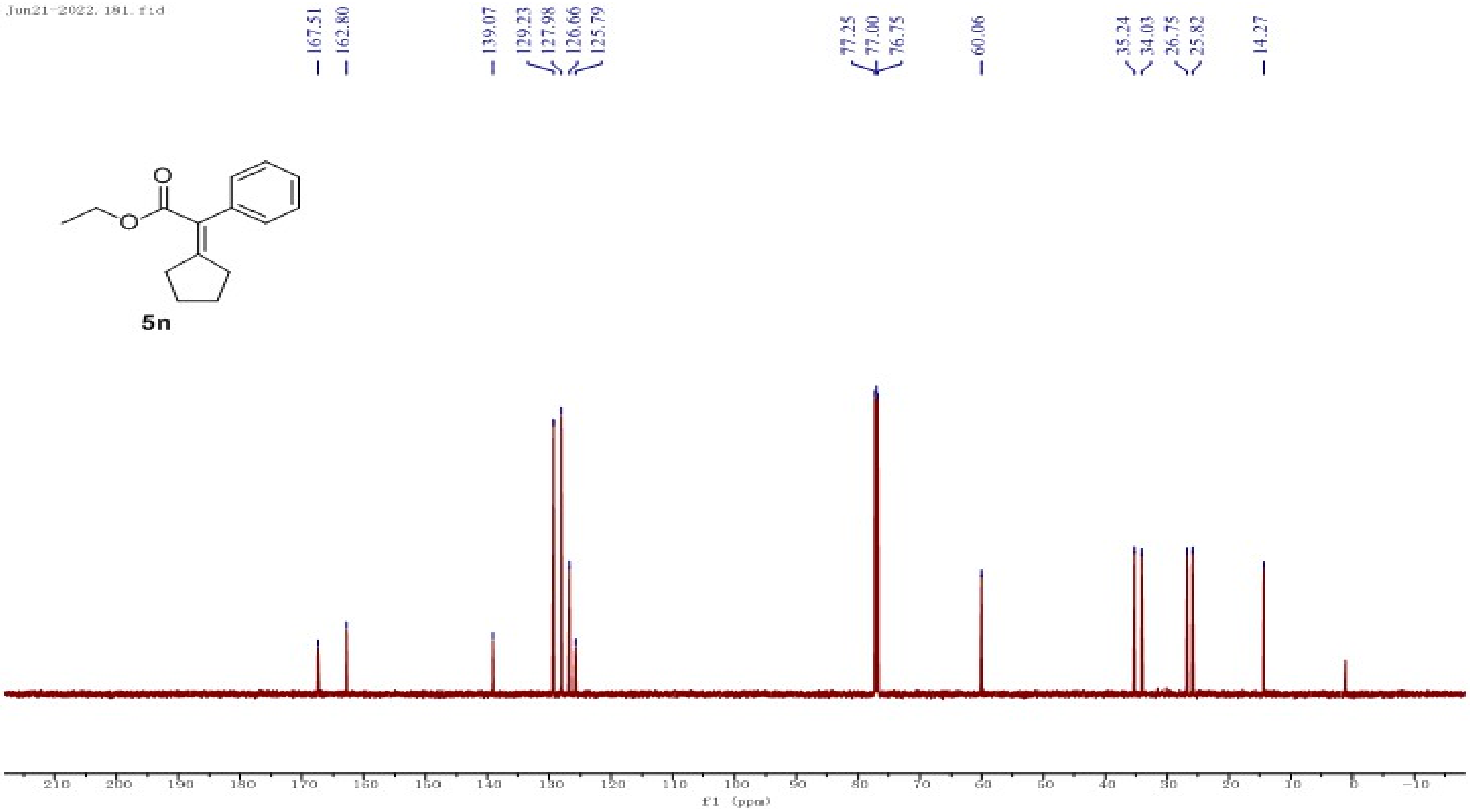
Jun21-2022.181.fid
167.51
162.80
139.07
129.23
127.98
126.66
125.79
77.25
77.00
76.75
60.06
35.24
34.03
26.75
25.82
14.27
O
O
5n
210
200
190
180
170
160
150
140
130
120
110
100
90
80
70
60
50
40
30
20
10
0
-10
f1 (ppm)

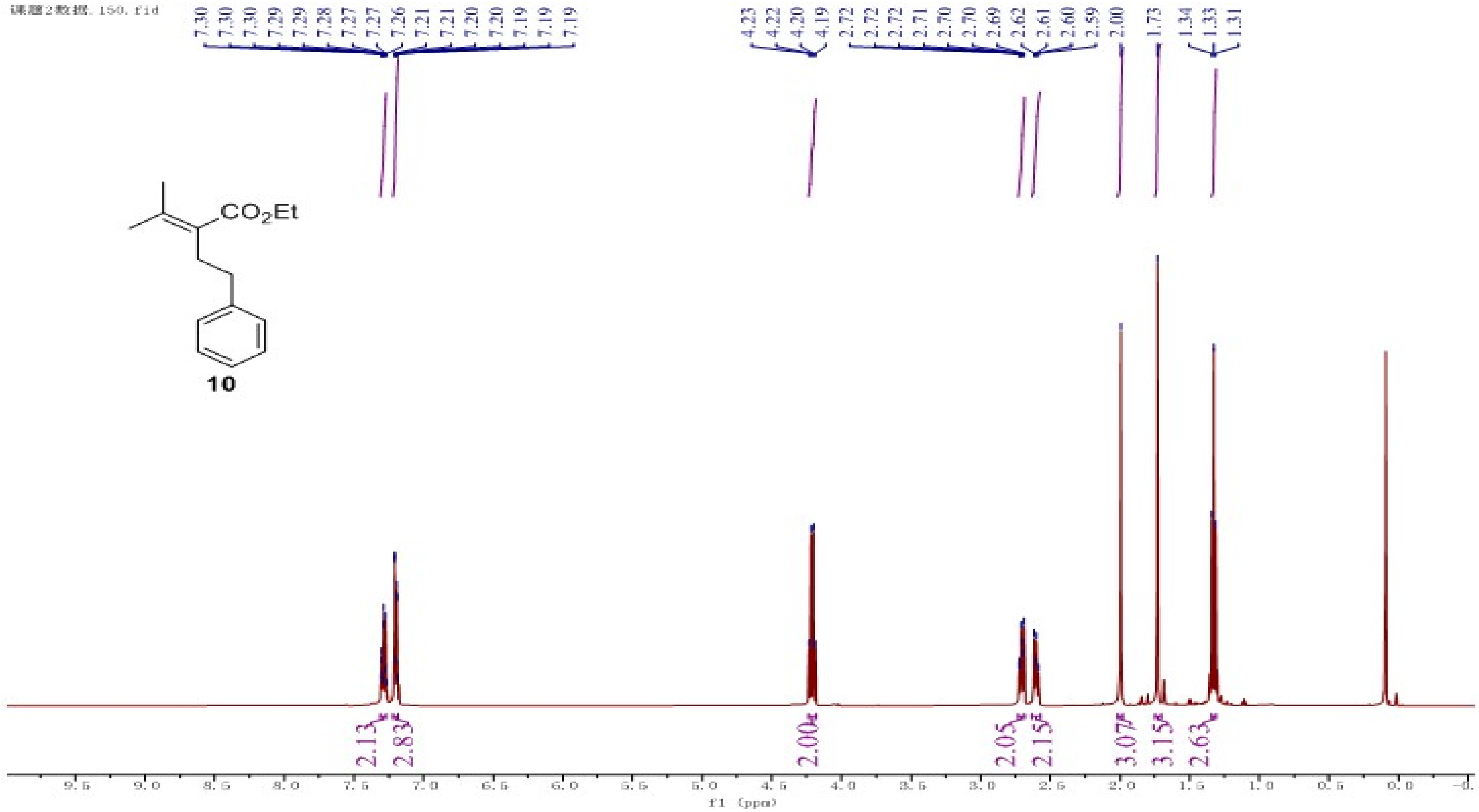
课题2数据.150.fid
CO2Et
10
f1 (ppm)

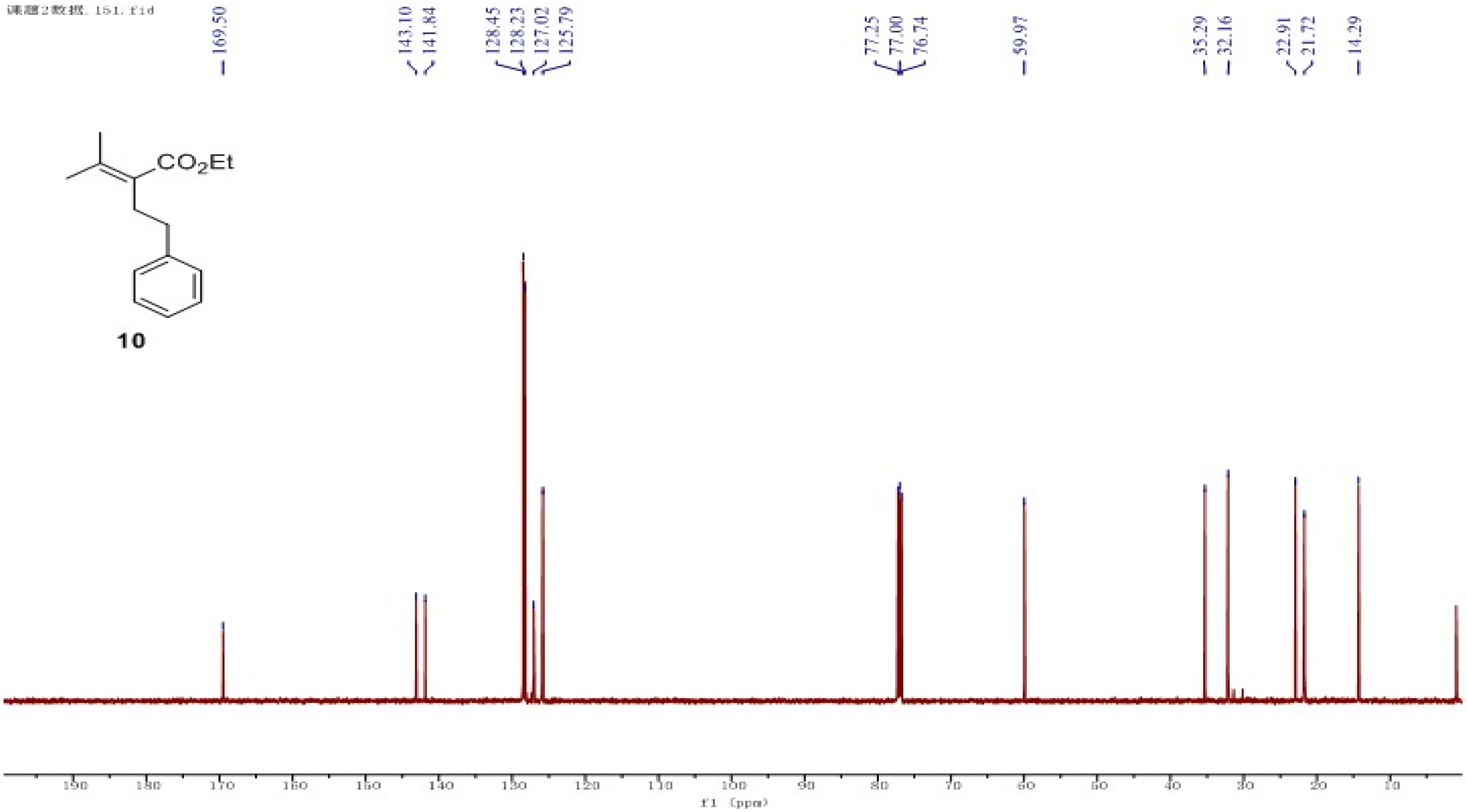

课题2数据.151.fid
169.50
143.10
141.84
128.45
128.23
127.02
125.79
77.25
77.00
76.74
59.97
35.29
32.16
22.91
21.72
14.29
CO2Et
10
190
180
170
160
150
140
130
120
110
100
90
80
70
60
50
40
30
20
10
f1 (ppm)
